# Cortical bone distribution of the proximal phalanges in great apes: implications for reconstructing manual behaviours

**DOI:** 10.1111/joa.13918

**Published:** 2023-06-26

**Authors:** Samar M. Syeda, Zewdi J. Tsegai, Marine Cazenave, Matthew M. Skinner, Tracy L. Kivell

**Affiliations:** ^1^ Skeletal Biology Research Centre, School of Anthropology and Conservation University of Kent Canterbury UK; ^2^ Department of Organismal Biology and Anatomy University of Chicago Chicago Illinois USA; ^3^ Division of Anthropology American Museum of Natural History New York New York USA; ^4^ Department of Anatomy, Faculty of Health Sciences University of Pretoria Pretoria South Africa; ^5^ Department of Human Origins Max Planck Institute for Evolutionary Anthropology Leipzig Germany

**Keywords:** cortical bone, functional morphology, internal bone structure, manual behaviour, phalangeal morphology, primates

## Abstract

Primate fingers are typically in direct contact with the environment during both locomotion and manipulation, and aspects of external phalangeal morphology are known to reflect differences in hand use. Since bone is a living tissue that can adapt in response to loading through life, the internal bone architecture of the manual phalanges should also reflect differences in manual behaviours. Here, we use the R package Morphomap to analyse high‐resolution microCT scans of hominid proximal phalanges of digits 2–5 to determine whether cortical bone structure reflects variation in manual behaviours between bipedal (*Homo*), knuckle‐walking (*Gorilla*, *Pan*) and suspensory (*Pongo*) taxa. We test the hypothesis that relative cortical bone distribution patterns and cross‐sectional geometric properties will differ both among extant great apes and across the four digits due to locomotor and postural differences. Results indicate that cortical bone structure reflects the varied hand postures employed by each taxon. The phalangeal cortices of *Pongo* are significantly thinner and have weaker cross‐sectional properties relative to the African apes, yet thick cortical bone under their flexor sheath ridges corresponds with predicted loading during flexed finger grips. Knuckle‐walking African apes have even thicker cortical bone under the flexor sheath ridges, as well as in the region proximal to the trochlea, but *Pan* also has thicker diaphyseal cortices than *Gorilla*. Humans display a distinct pattern of distodorsal thickening, as well as relatively thin cortices, which may reflect the lack of phalangeal curvature combined with frequent use of flexed fingered hand grips during manipulation. Within each taxon, digits 2–5 have a similar cortical distribution in *Pongo*, *Gorilla* and, unexpectedly, *Homo*, which suggest similar loading of all fingers during habitual locomotion or hand use. In *Pan*, however, cortical thickness differs between the fingers, potentially reflecting differential loading during knuckle‐walking. Inter‐ and intra‐generic variation in phalangeal cortical bone structure reflects differences in manual behaviours, offering a comparative framework for reconstructing hand use in fossil hominins.

## Graphical abstract

Cortical bone of the proximal phalanges reflects differences in locomotor and manipulative behaviours of extant great apes.

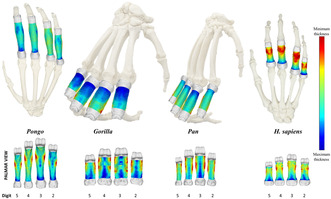

## 1 INTRODUCTION

As the primate hand, and particularly the fingers, interacts directly with the external environment, they have the potential to provide functional information about both locomotion and/or manipulation. Studies exploring phalangeal external morphology (Inouye, 1994; Matarazzo, 2008; Patel & Maiolino, 2016; Rein, 2011; Rein & McCarty, 2012; Susman, 1979), phalangeal curvature (Jungers et al., 1997; Richmond, 2007; Stern et al., 1995) and internal bone architecture of the wrist (Bird et al., 2021, 2022; Tocheri et al., 2007), metacarpals (Dunmore et al., 2019; Stephens et al., 2018; Tsegai et al., 2013; Zeininger et al., 2011) and phalanges (Matarazzo, 2015; Stephens et al., 2018) have demonstrated a functional signal between the external and/or internal morphology of the hand and manual behaviours (Kivell, 2015). The functional link between internal bone structure and locomotor behaviour has been established in several skeletal elements (Arias‐Martorell et al., 2021; Cotter et al., 2009; Saers et al., 2016; Scherf et al., 2013; Tsegai, Skinner, et al., 2017), however, the internal architecture of the manual phalanges remains relatively understudied, despite the phalanges of digits 2–5 being involved in grasping during both locomotion and manipulation (Bardo et al., 2017; Byrne & Byrne, 2001; Marzke, 1997; Matarazzo, 2013; Neufuss et al., 2017). Here, we investigate variation in cortical bone structure of the proximal phalanges of digits 2–5 (PP2–PP5) in humans and other extant hominids.

Much of the work to date exploring fossil and extant primate phalangeal morphology has focused on quantifying variation in shaft curvature, as it is considered to be functionally informative about hand use during locomotion and particularly differences in arboreality (Deane & Begun, 2008; Jungers et al., 1997; Matarazzo, 2008; Richmond, 1998; Rein, 2011; Stern et al., 1995; Stern & Susman, 1983; Susman et al., 1984; but see Wallace et al., 2020). During grasping, longitudinally curved phalanges are thought to be more effective than straight phalanges because the curvature helps to reduce bending moments by aligning the bone more closely with the joint reaction force (Oxnard, 1973; Preuschoft, 1973). Finite element (FE) modelling techniques have validated these functional hypotheses regarding phalangeal curvature by testing differences in strain distribution in curved versus mathematically straightened phalanges, revealing curved phalanges experience overall lower strain (Nguyen et al., 2014; Richmond, 2007). Furthermore, the degree of phalangeal curvature changes throughout ontogeny depending on mechanical loading (Richmond, 1998, 2007). For example, juvenile chimpanzees and gorillas have a higher degree of phalangeal curvature than adults (Richmond, 1998; Sarringhaus, 2013), reflecting a decrease in arboreality throughout ontogeny (Doran, 1997). This research suggests a strong functional link between locomotor behaviour and the external morphology of phalanges (but see Wallace et al., 2020).

In contrast to research on phalangeal external shape, the functional relationship between the internal bone morphology of phalanges and locomotor behaviour has yet to be thoroughly explored. Internal bone architecture consists of cortical and trabecular bone, both of which are subject to epigenetic changes that result from loading experienced by the bone during an individual's lifetime; a process known as bone functional adaptation (Currey, 2003; Pearson & Lieberman, 2004; Ruff et al., 2006). Cortical bone adapts to the functional demands placed upon it through adjustments to its mineralization to adapt its stiffness and changes in overall shape to resist loads or by increasing its thickness (Currey, 2003; Ruff et al., 2006). Overall, both cortical and trabecular bone adapt in response to their mechanical environment by removing bone in skeletal areas where stress is low and adding bone where stress is high (Pearson & Lieberman, 2004; Ruff et al., 2006).

Cortical bone is usually studied through analysis of cross‐sectional geometric (CSG) properties that offer robust estimations of strength and rigidity of a bone (Ruff et al., 2006; Ruff & Runestad, 1992). Understanding how CSG patterns correlate with loading regimes of an individual is complex and drawing functional interpretations can be challenging, but CSG patterns provide an indirect method to understand potential loading patterns when direct biomechanical data are not available or not possible to measure. Recently, studies of cortical thickness distribution of long bones have also revealed that the cortex varies throughout the shaft across different skeletal elements in ways that relate to locomotor behaviour (Cazenave et al., 2019; Jashashvili et al., 2015; Puymerail, 2013; Tsegai, Stephens, et al., 2017; Wei et al., 2021). Combining the analysis of CSG with cortical bone distribution and thickness can allow inference of bone adaptation in relation to habitual loading (Jashashvili et al., 2015).

Within the hand, only cortical structure of the metacarpals has been studied in extant hominids (Dunmore et al., 2020; Marchi, 2005; Patel et al., 2020), which found cross‐sectional properties can distinguish habitual locomotor behaviours of extant great apes. Several studies have also explored the functional morphology of trabecular bone in the carpals and metacarpals (Bird et al., 2021, 2022; Dunmore et al., 2019; Schilling et al., 2014; Tsegai et al., 2013). However, to date, there have only been three studies published to our knowledge that have explored the internal bone structure of proximal phalanges of the fingers (Doden, 1993; Matarazzo, 2015; Stephens et al., 2018). Doden (1993) studied the internal cortical structure of the phalanges in gibbons and humans, noting a functional link between the shape and density of cortical bone and manual behaviours. Matarazzo (2015) analysed the trabecular architecture at the proximal and distal epiphysis of the phalanges of digit 3 in extant non‐human hominoids and macaques, with patterns of trabecular orientation differing between the locomotor modes of the taxa. However, other variables of trabecular bone (e.g., bone volume fraction, degree of anisotropy, isotropy index) in the phalanges failed to distinguish between locomotor behaviours (Matarazzo, 2015). Stephens et al. (2018) documented variation in the structure of trabecular bone in post‐Neolithic and foraging human hands, revealing greater trabecular bone volume fraction in foragers that is consistent with higher intensity loading than that experienced by post‐Neolithic individuals. Therefore, the analysis of the internal bone structure of manual phalanges of extant great apes holds potential for reconstructing the behaviour of fossil hominin species. However, there has yet to be a detailed analysis of variation in cortical thickness in hominid phalanges, which is important to consider in light of differences in trabecular structure (Matarazzo, 2015; Stephens et al., 2018) and phalangeal curvature (Jungers et al., 1997; Matarazzo, 2008; Rein, 2011; Richmond, 1998; Stern et al., 1995; Wennemann et al., 2022).

Here, we conduct a detailed examination of cortical structure of the proximal phalanges of digits 2–5 in extant hominids. We assume phalangeal cortical bone morphology in non‐human hominids will primarily reflect locomotor loading. This is due to the high mechanical loads on the fingers from dynamic loading and body mass that occur during locomotion (Preuschoft, 2019). Although all non‐human hominids show enhanced manual dexterity and tool use abilities in the wild (e.g., Byrne & Byrne, 2001; Lesnik et al., 2015; Marzke et al., 2015; Van Schaik et al., 1996) and captivity (e.g., Bardo et al., 2016, 2017; Pouydebat et al., 2005), we assume that loading during manipulation will be lower than that of locomotion. In contrast, we assume human phalangeal cortical structure will reflect loading during manipulation given the rarity with which individuals in our sample likely used their hands for locomotion.

### 1.1 Predictions

This study examines the cortical structure of the proximal manual phalanges of digits 2–5 to determine whether variation in manual behaviours associated with locomotion and manipulation correlates with cortical bone properties in *Pongo*, *Gorilla*, *Pan* and *Homo sapiens*, and how potential differences in cortical thickness vary with differences in phalangeal curvature. We quantify both variation in cortical thickness throughout the phalangeal shaft and cross‐sectional geometric properties at sections along the shaft (35%, 50% and 65% of bone length). We test three main predictions regarding variation in cortical bone structure based on observations of great ape, including humans, manual behaviour, bone functional adaptation and studies on phalangeal external morphology and biomechanics.

Our first prediction is that relative cortical bone distribution patterns will significantly differ among extant great apes due to locomotor and postural differences. Second, we predict that across the four digits of each species, there will be variation in cortical bone thickness distribution, mean cortical bone thickness and CSG properties. Finally, we predict that mean cortical bone thickness and cross‐sectional properties will significantly differ across the great apes. We discuss these specific predictions for each taxon below.

**FIGURE 1:**
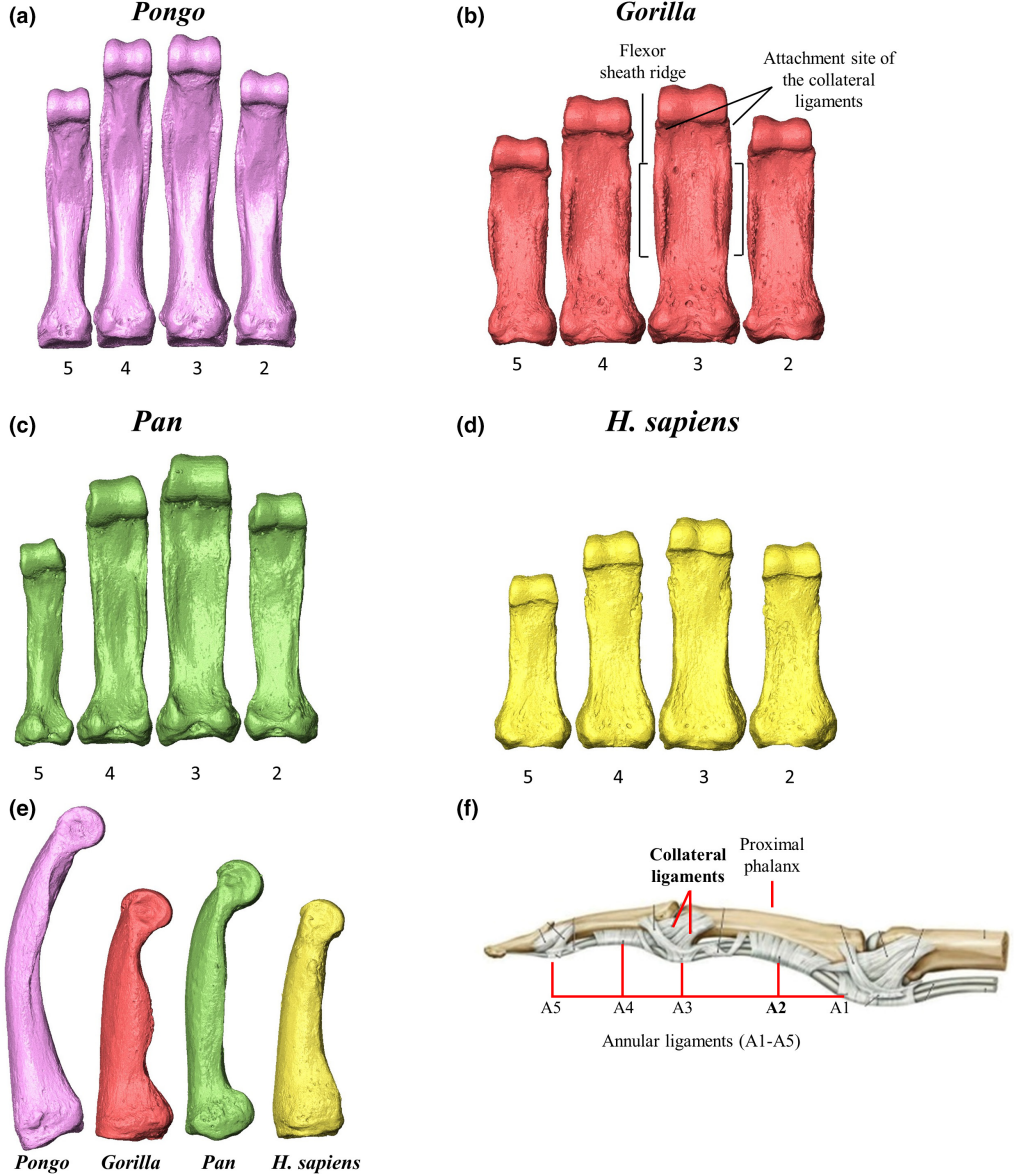
Representative 3D surfaces of proximal phalanges of (a) *Pongo pygmaeus*, (b) *Gorilla gorilla*, (c) *Pan troglodytes*, (d) *Homo sapiens*. Digits 2–5 are represented from right to left. The proximal phalanges have been scaled to relative size. (e) Medial surface of the third proximal phalanx of each taxa. Variation in curvature and flexor sheath ridge morphology is evident. (f) Depiction of ligaments of the finger. The second annular pulley (A2) and collateral ligament of the PIP joint are highlighted in subset F (modified from Gilroy & MacPherson, 2016) and the flexor sheath ridges and attachment sites of the collateral ligaments are shown in subset B.

*Pongo* is highly arboreal, with torso‐orthograde suspension dominating their complex postural and locomotor behaviours (Cant, 1987; Thorpe et al., 2009; Thorpe & Crompton, 2006). During suspension, the hand is positioned like a hook around the substrate, which may mitigate bending stress during suspension, because joint reaction forces load the articular ends of the phalanges dorsally in compression, while the forces from the digital flexor muscles, along with the joint reaction and gravitational forces, pull the phalanges palmarly (Carlson & Patel, 2006; Richmond, 2007; Schmitt et al., 2016). In *Pongo* phalanges, the high degree of longitudinal curvature (Figure 1), combined with flexor sheath ridges (FSRs) located opposite the maximum arc of curvature, are thought to be advantageous for frequent flexed finger grasping (Susman, 1979). Thus, we predict *Pongo* will exhibit a pattern of maximum thickness on the disto‐palmar surface of the phalangeal shaft, as the proximal phalanges are most often being loaded in flexed finger grasping during locomotion and are experiencing tensile and compressive forces from the joint reaction forces and substrate reaction forces (Matarazzo, 2015; Nguyen et al., 2014; Preuschoft, 1973; Tsegai et al., 2013). We predict that this cortical distribution pattern, as well as mean cortical bone thickness and CSG properties, will be similar across the four digits, as all four digits are thought to be used in a similar manner during manual behaviours (Rose, 1988 but see Mcclure et al., 2012). Across the great apes, we expect cortical properties, associated with strength and rigidity against bending and torsional loads, of *Pongo* to be less than that of the African apes as the external phalangeal morphology helps mitigate stress from arboreal locomotion.

*Gorilla* engage primarily in knuckle‐walking (Doran, 1996, 1997; Inouye, 1994; Tuttle & Watts, 1985), during which the dorsal surfaces of the intermediate phalanges are in contact with the substrate and the proximal phalanges, metacarpals and body mass of the animal are elevated above the hand (Preuschoft, 1973; Tuttle, 1967; Wunderlich & Jungers, 2009). Zoo‐housed *Gorilla* most often use a palm‐back (pronated) position and experience relatively even pressure across digits 2–5 (Matarazzo, 2013; Tuttle, 1969), while wild *Gorilla* have been observed to have more variable hand postures (Thompson et al., 2018). The radio‐ulnarly wide, stout and flat phalanges are thought to reflect these frequent knuckle‐walking hand postures. The proximal phalanges also have prominent FSRs, indicating forceful grasping during arboreal locomotion and/or food processing (Neufuss et al., 2019; Remis, 1998; Susman, 1979; Tuttle & Watts, 1985). We predict that the cortical thickness pattern of *Gorilla* will be similar palmarly and dorsally due to loading of a flexed proximal interphalangeal (PIP) and hyper‐extended metacarpophalangeal (McP) joint (Tsegai et al., 2013). Across digits 2–5, we expect no differences in cortical thickness and cross‐sectional properties, due to the similar pressure experienced by digits 2–5 during knuckle‐walking (Matarazzo, 2013). Relative to *Pongo* and *H. sapiens*, the phalanges of *Gorilla* are predicted to have thicker cortices and stronger CSG properties, as the phalanges are incurring ground reaction forces from locomotion and joint reaction forces resulting from the contraction of the finger flexor and extensor musculature, along with the gravitational forces supporting the body mass (Jenkins & Fleagle, 1975; Tsegai et al., 2013). However, it is important to acknowledge that wild mountain gorilla (*Gorilla beringei*) knuckle‐walking hand postures in their natural habitat are much more variable than those of zoo‐housed gorilla and they commonly use non‐knuckle walking hand postures (Thompson et al., 2018). These variable hand postures could result in different degrees of flexion/extension of the finger joints and more variable loading of the proximal phalanges (Thompson et al., 2018).

*Pan* (*Pan troglodytes* and *Pan paniscus*) also engages primarily in terrestrial knuckle‐walking but is more variable in its positional behaviour than *Gorilla*, both within and across populations (Doran, 1996; Doran & Hunt, 1996; Hunt, 2020; Sarringhaus et al., 2014). Zoo studies show that *P. troglodytes* use more variable hand postures than *Gorilla* (Inouye, 1994; Tuttle, 1969). In zoo‐housed *Pan*, digits 3 and 4 typically experience the highest loads during knuckle‐walking, while in some bouts of knuckle‐walking digit 5 does not touch down or experiences significantly less loading than the radial three digits (Matarazzo, 2013; Wunderlich & Jungers, 2009). Arboreal behaviours are more common in *Pan*, compared to *Gorilla*, but the frequency can vary substantially among sexes, communities and (sub)species (Doran, 1996; Doran & Hunt, 1996; Hunt, 2020; Ramos, 2014; Remis, 1998; Sarringhaus et al., 2014). *Pan* proximal phalanges show a greater degree of dorsal curvature than *Gorilla* (Figure 1), which may reflect an increased degree of arboreality in their locomotor repertoire (Susman, 1979; but see Wallace et al., 2020). However, the frequency of habitual knuckle‐walking is greater than arboreal behaviours (Doran & Hunt, 1996; Hunt, 2020) and, as such, knuckle‐walking signals will likely be reflected in the internal structure of manual phalanges. Thus, we predict *Pan* and *Gorilla* will share a similar pattern of cortical bone distribution due to their similar locomotor repertoires, along with cortical thickness and CSG properties of strength and rigidity against loads that are greater than those of *Pongo* and *H. sapiens*. Within *Pan*, we expect relative differences in cortical thickness and properties across the digits due to the more variable hand postures employed during their locomotor repertoire (Doran & Hunt, 1996; Matarazzo, 2013; Wunderlich & Jungers, 2009).

Humans are unique among great apes in using their hands mainly for manipulation, rather than locomotion. Forceful precision grips, power squeeze grips and precise in‐hand manipulation are important in stone tool making and use and are thought to distinguish modern human manipulatory abilities from other hominids (Marzke, 1997; Williams‐Hatala, 2016). Across modern human adults, power grips are employed most frequently during daily activities (Dollar, 2014; Feix et al., 2015). Power grips require the fingers to be in flexion, with experimental studies quantifying the biomechanics of power grips revealing that joint forces increase disto‐proximally and digit 2 experiences the greatest loads followed by digits 3, 4 and 5 (De Monsabert et al., 2012; Sancho‐Bru et al., 2014; Vigouroux et al., 2011). Human proximal phalanges are gracile and lack dorsopalmar curvature and strong muscle markings (Patel & Maiolino, 2016; Susman, 1979), likely reflecting lower loads incurred during manipulation compared with those of locomotion. We predict the pattern in *H. sapiens* will be of maximum thickness in the dorsal aspect of the shaft, as the straight proximal phalanges are typically in a flexed position during manipulation (Marzke, 1997; Rolian et al., 2011) and are experiencing bending stresses (Doden, 1993; Nguyen et al., 2014; Richmond, 2007), which are concentrated on the dorsal surface in straight phalanges. We also predict humans to show greater variability across the digits due to the frequent loading of digits 2 and 3 during daily manipulative activities (De Monsabert et al., 2012; Sancho‐Bru et al., 2014). Finally, cortical thickness and CSG properties, associated with strength and rigidity against bending and torsional loads, of *H. sapiens* are predicted to be lower than that of the other great apes as humans most frequently use their hands for manipulation (Marzke, 2013; Tocheri et al., 2008).

## 2 METHODS

### 2.1 Study sample

The study sample consists of manual proximal phalanges from digit 2 (*n* = 80 elements), digit 3 (*n* = 86 elements), digit 4 (*n* = 83 elements) and digit 5 (*n* = 70 elements) of *H. sapiens* (*n* = 34 individuals), *Pan* (*n* = 24 individuals, including *P. troglodytes* and *P. paniscus*), *Gorilla gorilla* (*n* = 25 individuals) and *Pongo* (*n* = 9 individuals, including *Pongo abelii* and *Pongo pygmaeus*) (Table 1). Details of the study sample are shown in Table S1 and representative morphology of each taxon is depicted in Figure 1. All non‐human apes were wild individuals with no obvious signs of pathologies within their hand skeletons or upper limbs. Our human sample originates from diverse post‐industrial populations including 20th century Syracuse, Italy (*n* = 2 individuals), 18th–19th century Inden, Germany (*n* = 5), 16th century males of the Mary Rose shipwreck (*n* = 7). It also includes pre‐industrial populations including 6th–11th century Nubian Egyptians (*n* = 4), 19th century Tierra del Fuego (*n* = 3), an indigenous Inuit from Greenland and two Aboriginal Australians. We also included in our *H. sapiens* sample several fossil *H. sapiens* including Qafzeh 8 and 9 (*n* = 2 individuals, 80–130 Ka, Qafzeh, Israel; Niewoehner, 2001), Ohalo II H2 (*n* = 1, 19 Ka, Sea of Galilee, Israel; Hershkovitz et al., 1995), Barma Grande (*n* = 1, 15–17 Ka, Ventimiglia, Italy; Churchill & Formicola, 1997), Arene Candide (*n* = 1, 12–11 Ka, Liguria, Italy; Sparacello et al., 2021) and Dolní Věstonice (*n* = 4, 31 Ka, Dolní Věstonice, Czech Republic; Fewlass et al., 2019).

**TABLE 1:** Summary of study sample included in the study.

Taxon	*N*	PP2	PP3	PP4	PP5
*Homo sapiens*	33	22	26	27	21
*Pan paniscus*	7	7	7	7	6
*Pan troglodytes*	17	16	17	17	12
*Gorilla gorilla*	25	23	23	20	21
*Pongo abelii*	2	2	2	2	2
*Pongo pygmaeus*	7	7	7	7	6

### 2.2 MicroCT scanning

All phalanges were scanned with high‐resolution micro‐computed tomography (microCT) using a BIR ACTIS 225/300, Diondo D3 or Skyscan 1172 scanner housed at the Department of Human Evolution, Max Planck Institute for Evolutionary Anthropology (Leipzig, Germany), a Nikon 225/XTH scanner at the Cambridge Biotomography Centre, University of Cambridge (Cambridge, UK) or with a Diondo D1 scanner at the Imaging Centre for Life Sciences University of Kent (Canterbury, UK). The scan parameters included acceleration voltages of 100–160 kV and 100–140 μA using a 0.2–0.5 mm copper or brass filter. Scan resolution ranged between 0.018 mm and 0.044 mm depending on the size of the bone. Images were reconstructed as 16‐bit TIFF stacks.

### 2.3 Data processing

**FIGURE 2:**
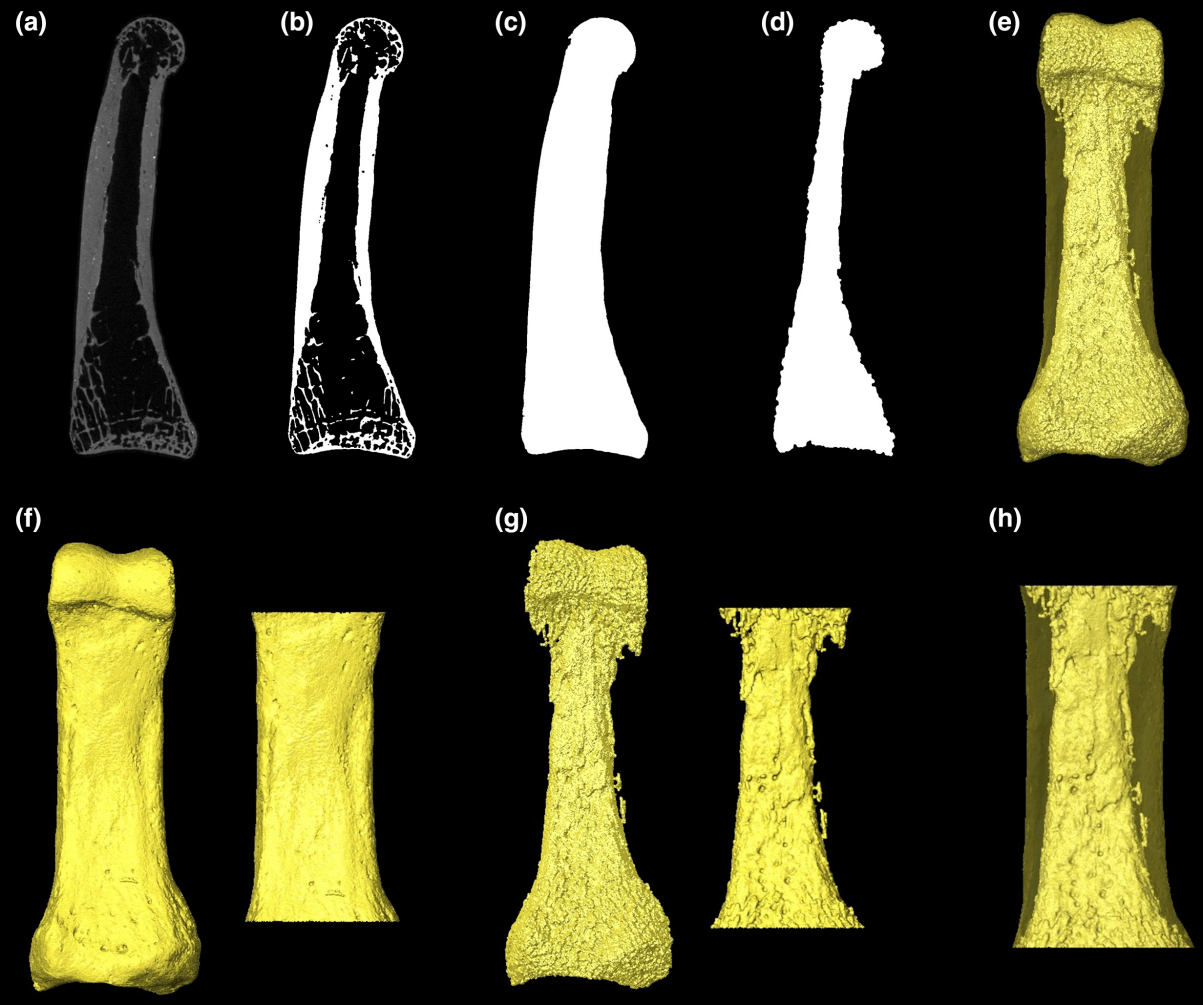
Steps taken to create surfaces for cortical thickness analysis. In Medtool 4.5 morphological filters were applied in the following steps: (a) Original microCT data of a *Homo sapiens* fourth proximal phalanx, (b) microCT data after MIA segmentation, (c) creation of outer layer of the cortex, (d) creation of inner layer of the cortex, (e) creation of an external (cortical) 3D surface from step c and an internal 3D surface from step d. Following surface creation, using Avizo Lite 9.0.0 the external and internal surfaces were cut (f and g) to define the shaft of the phalanx and (h) create cut surfaces for cortical bone thickness analysis in morphomap.

Non‐bone inclusions or remaining soft tissues were removed from the scans and each phalanx was rotated into a standard orientation using Avizo Lite 9.0.0 (Visualization Sciences Group, SAS). Scans were subsequently segmented using the medical image analysis (MIA) clustering method (Dunmore et al., 2018). Once segmented, the outer and inner layers of the cortex were defined using Medtool v 4.5 (www.dr‐pahr.at/medtool), following Tsegai et al. (2013) and Gross et al. (2014). This involves use of a ray‐casting method to isolate the external and internal edges of the cortex in 3D and morphological filters to fill the bone, resulting in a mask of the inner and outer regions of the cortex. Smooth external and internal surfaces of these voxel data were created using a custom script in Paraview v 4.4 and Meshlab v 2020.03 (Figure 2). Six *Pan* and five *Gorilla* phalanges were excluded from the study sample (i.e., not included in sample sizes listed earlier) because their cortices were so thickened distally (i.e., almost completely filling the medullary cavity) that it did not allow for the creation of a distal internal surfaces because the rays could not detect a non‐bone voxel.

### 2.4 Cortical bone analysis

This study quantifies cortical bone distribution patterns and CSG parameters using the R package morphomap (Profico et al., 2021). In brief, morphomap allows the user to divide a 3D mesh of a long bone surface into a certain number of cross‐sections and place a desired number of landmarks on the periosteal and endosteal outlines of the bone. The landmark data allow for the quantification and mapping of cortical bone thickness, while the associated periosteal and endosteal outlines of each slice are used to measure CSG properties.

#### 2.4.1 Morphomap parameters

Morphomap is designed to produce cross‐sections across a certain percentage of the bone defined by the user (Profico et al., 2021). Since this study quantifies cortical thickness of the phalangeal shaft across species of varying morphology, there was not a standardized percentage of phalangeal length that we could consistently define as the shaft across all individuals/taxa. Variation in the shape and size of the proximal phalanx base and the trochlea meant that these features extended onto the diaphysis to differing degrees (Figure 1). Thus, to compare homologous structures, we defined a region of interest (ROI) of the shaft as between the distal most extent of the base and the proximal end of the trochlea individually for each specimen.

**FIGURE 3:**
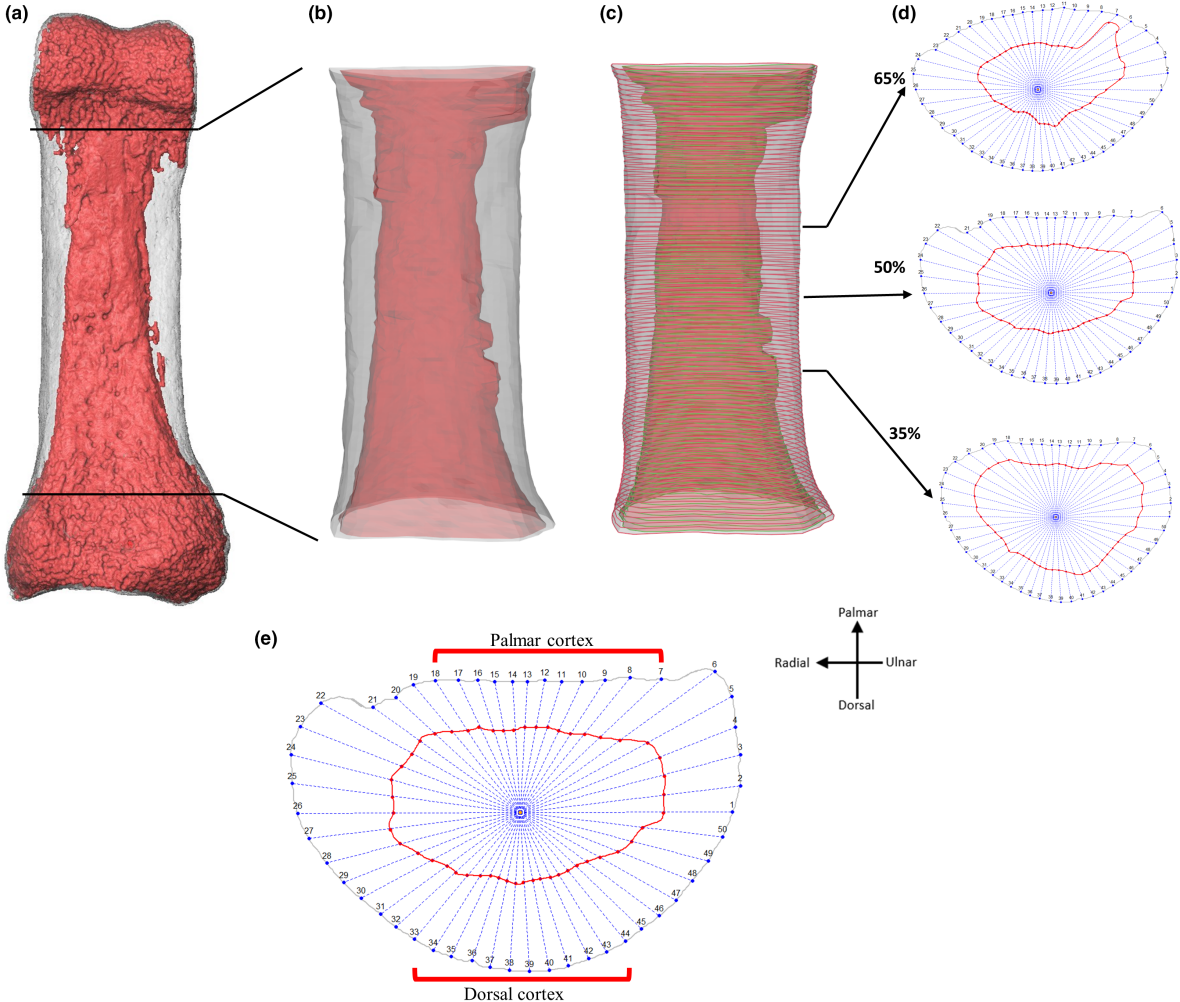
Data acquisition in *morphomap*. (a) External (grey) and internal (red) 3D surface models of proximal phalanx of digit 4 in an *Homo sapiens* individual. (b) Cut external and internal 3D surfaces defining the shaft (as defined in text) for cortical thickness quantification in morphomap. (c) Cortical bone parameters are measured in 1% cross‐sectional increments along the shaft and arrows indicate cross‐section locations (35%, 50%, 65%) where CSG parameters were analysed. (d) Cross‐sections at 35%, 50% and 65% of the bone length. At each cross‐section, 50 semi‐landmarks were placed on the external and internal surfaces equiangularly and were used to calculate cortical thickness. (e) Landmarks used to divide the cortex into palmar and dorsal cortex.

The ROI was defined based on the external morphological features outlined earlier, both in palmar and lateral views, to ensure the greatest extent of the trochlea or base was not included in the ROI. The external and internal surfaces were cropped using Avizo Lite 9.0.0 (Visualization Sciences Group, SAS), however, as morphomap required a slight buffer on either end of the cropped ROI, this crop was at 2% above and below the defined shaft, so cortical thickness could be mapped across the entire ROI (Figures 2f–h and 3a–c). Within morphomap, the cut external and internal ROIs were used to extract 97 sections at increments of 1% between 2% and 98% of the ROI length (i.e., the defined shaft length). At each cross‐section, 50 paired equiangular semi‐landmarks, centred around the cortical area of each cross‐section, were placed on the outlines of the external and internal surfaces to accurately capture the complex morphology of the phalangeal shaft. The combination of cross‐sections and the landmarks placed on them allow a set of lines to be drawn from the centroid of each slice outwards to the landmarks placed on the internal and external outlines of the 3D surfaces (Profico et al., 2021). Using these lines, cortical thickness is calculated as the length of the line between the internal and external surface outlines.

Along with measuring cortical thickness along the entire shaft, we also measured cortical thickness of landmark‐defined palmar and dorsal surfaces of the shaft, which was assessed as a ratio of palmar/dorsal mean thickness. This allowed comparison of cortical thickness across genera without the influence of variation in size or shape of the FSRs, which are not represented by the dorsal and palmar landmarks. This morphology was defined by selecting an equal number of landmarks on the palmar and dorsal surfaces of the shaft, but excluding the medial or lateral aspects of the bone, where the FSRs are located (Figure 3e). To visualize the pattern of cortical bone distribution, morphometric maps of cortical thickness for each individual were created using R package morphomap.

#### 2.4.2 Cross‐sectional geometry

Cross‐sectional geometric properties were calculated at each slice across the shaft with the R package morphomap. Different CSG properties quantify different aspects of the diaphysis and the most commonly used properties to understand the dynamic loads incurred by locomotion are cortical area (CA; measure of axial strength), polar moment of area (J; measure of bending and torsional rigidity) and polar section modulus (Z_pol_; measure of maximum bending strength) (Lieberman et al., 2004; Marchi, 2005; Patel et al., 2020; Ruff & Runestad, 1992; Schaffler et al., 1985; Trinkaus & Ruff, 2012). We studied these cross‐sectional properties at three positions along the shaft (35%, 50% and 65% of the shaft length) of each phalanx to quantify variation in cortical robusticity within the phalangeal shaft. The specific cross‐sections were chosen to account for variation in the proximodistal extension of the base and trochlear morphology across our sample and to ensure each cross‐section sampled only the diaphysis.

### 2.5 Phalangeal curvature

The degree of phalangeal curvature was measured using the included angle (IA) method. The IA (𝜃) method assumes the curvature of a phalanx in the dorsopalmar direction is represented by an arc length on the perimeter of a circle (Stern et al., 1995). Low values of 𝜃 are characteristic of straighter phalanges, commonly associated with quadrupedalism and bipedalism, and higher values of 𝜃 are characteristic of increasingly curved phalanges, commonly associated with arboreality (Jungers et al., 1997; Stern et al., 1995). The IA method was chosen as it has been the most prevalent approach to calculate phalangeal curvature and does well to distinguish the locomotor behaviours of species (Jungers et al., 1997; Matarazzo, 2008; Rein, 2011; Stern et al., 1995). However, it is important to note that the IA method is susceptible to measurement errors (Deane & Begun, 2008; Patel & Maiolino, 2016), therefore three repeated measurements were taken to correct for intra‐observer measurement error.

### 2.6 Statistical analyses

As larger bones and individuals will potentially have higher absolute values of cortical bone and larger cross‐sections, we scaled the data by the length of the bone. Phalangeal length was measured digitally on surface models in Avizo 9.0., from the most proximal extent of the base to the most distal extent of the trochlea in dorsal view. All statistical analyses were conducted on the scaled data, as well as on raw data for intra‐generic comparisons.

#### 2.6.1 Cortical thickness distribution pattern

Cortical thickness values were calculated from a measurement between each pair of corresponding landmarks at the inner and outer cortical surfaces on each slice of the defined shaft, resulting in 4850 measurements per phalanx. To explore differences in the distribution of cortical bone thickness between taxa, each of the 4850 measurements were treated as a variable in a principal component analysis (PCA). To test if cortical thickness distribution patterns of each taxon were significantly different from each other, an omnibus permutational multivariate analysis of variance was run on the first three PC scores using the R package Vegan. If this test was statistically significant (*p* < 0.05), it was followed by a pairwise one‐way permutational multivariate analysis of variance with a Bonferroni correction to test which groups were significantly different from one another. Permutational multivariate analysis of variance tests were conducted because Shapiro–Wilk tests revealed that not all data were normally distributed.

#### 2.6.2 Mean cortical thickness

Inter‐ and intra‐generic differences in mean cortical thickness were assessed using Kruskal–Wallis tests, as Shapiro–Wilk tests revealed the data were not normally distributed, followed by a post hoc Dunn test. Inter‐generic testing was conducting on each digit separately.

#### 2.6.3 Cross‐sectional geometric properties

Intra‐generic differences in cross‐sectional properties (CA, Z_pol_ and J) at the three diaphyseal positions (35%, 50%, 65%) across the digits of each taxon were compared using a Kruskal–Wallis test, followed by a post hoc Dunn test separately, along with intra‐generic differences in diaphysis position within each digit. Inter‐generic differences in cross‐sectional geometric properties were assessed for each property at each position for each digit using a Kruskal–Wallis test, followed by a post hoc Dunn test.

#### 2.6.4 Relationship between curvature and cortical thickness

Regression analyses were used to test the relationship between phalangeal curvature (IA values) and mean cortical thickness for each taxon. For each taxon, all four digits were pooled together to increase the sample size and to produce a more reliable fit of the regression model.

All statistical tests were performed using the R package RVAideMemoire (v 0.9‐79 Hervé, 2022), Stats (R Core Team, 2020) and FSA (v 0.9.3 Ogle et al., 2022). Statistical tests were carried out in R version 4.1.3 and all tests were considered statistically significant with a *p* < 0.05.

## 3 RESULTS AND DISCUSSION

**FIGURE 4:**
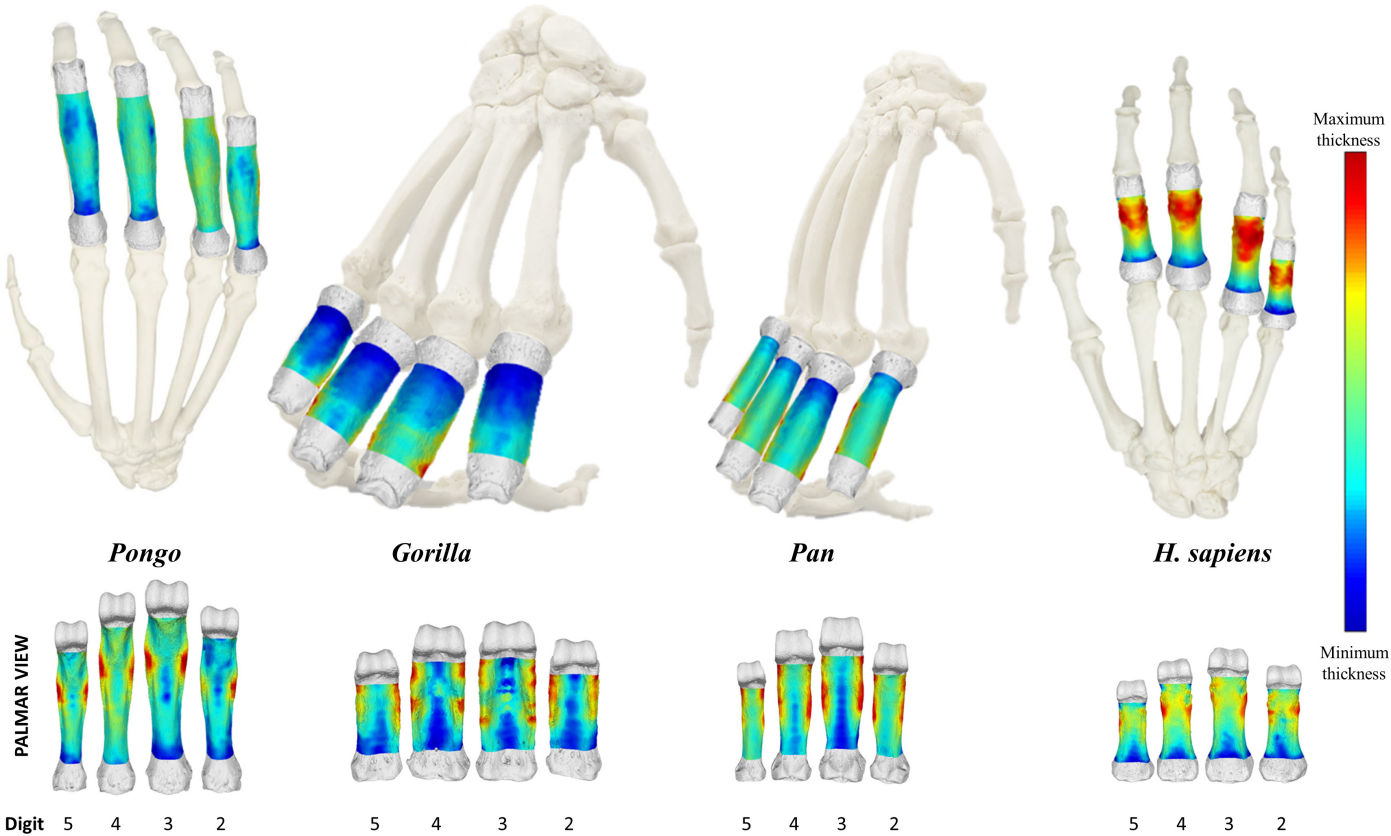
Representative 3D maps of cortical bone distribution of proximal phalanges of digits 2–5 of *Pongo pygmaeus, Gorilla gorilla*, *Pan troglodytes*, *Homo sapiens* in dorsal (top) and palmar (bottom) view. Thickness maps of each bone are independent of each other. Proximal phalanges are not scaled.

**FIGURE 5:**
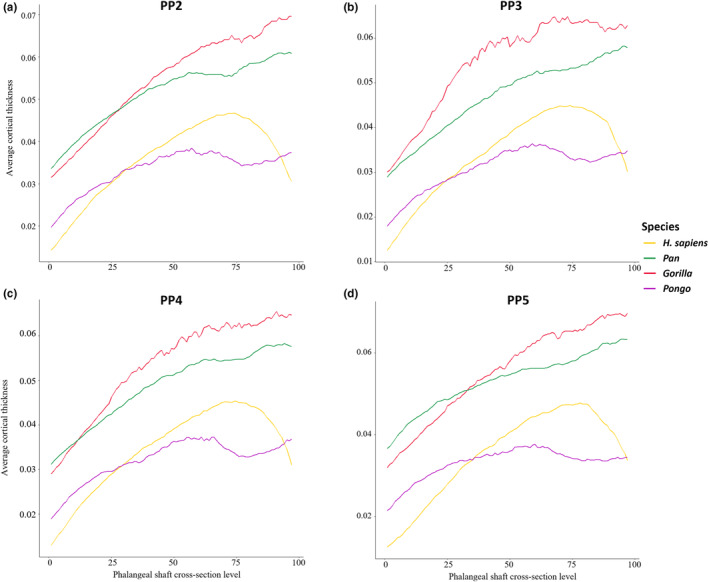
Average scaled cortical bone thickness plotted from the proximal end (0) to the distal end (100) of the phalangeal shaft of *Homo sapiens*, *Pan*, *Gorilla* and *Pongo*. (a) PP2; (b) PP3; (c) PP4; (d) PP5.

This study explored the relationship between expected loading during various locomotor and hand‐use behaviours and the cortical structure of non‐pollical proximal phalanges in extant hominids. The distribution of cortical bone, as well as its overall thickness and CSG properties differed among genera, and across the digits within genera, in line with some of our predictions. These results support a relationship between cortical morphology of the manual phalanges and loading of the hand among great apes. Figure 4 depicts cortical thickness distribution morphometric maps of the proximal phalanges (digits 2–5) in a representative individual for each taxon, while morphometric maps for all individuals within our sample are presented in Figure S1. Figure 5 depicts average cortical thickness plotted across the shaft for each taxon and Table 2 shows mean values of cortical thickness. Table S2 shows mean values of all cross‐sectional properties across the three cross‐sections. Variation in cortical bone distribution patterns were assessed via PCA. This is followed by a description of cortical distribution patterns, as well as variation in cortical thickness and cross‐sectional properties for each study taxon.

**TABLE 2:** Summary statistics of raw (mm) and standardized (dimensionless) cortical thickness measurements of the phalangeal shaft.

	*Homo sapiens*	*Pan*	*Gorilla*	*Pongo*
	Mean (SD)	Mean (SD)	Mean (SD)	Mean (SD)
Raw				
PP2	1.477 (0.290)	2.520 (0.438)	2.862 (0.550)	2.078 (0.328)
PP3	1.561 (0.261)	2.679 (0.481)	3.220 (0.563)	2.187 (0.341)
PP4	1.507 (0.264)	2.605 (0.452)	2.924 (0.512)	2.212 (0.360)
PP5	1.199 (0.262)	2.257 (0.361)	2.556 (0.504)	1.981 (0.298)
Standardized^a^				
PP2	0.036 (0.007)	0.051 (0.007)	0.054 (0.006)	0.033 (0.005)
PP3	0.034 (0.006)	0.048 (0.008)	0.055 (0.006)	0.031 (0.004)
PP4	0.035 (0.006)	0.049 (0.007)	0.053 (0.006)	0.032 (0.005)
PP5	0.035 (0.007)	0.053 (0.008)	0.055 (0.008)	0.033 (0.004)

^a^ Standardized by bone length.

### 3.1 Cortical bone thickness distribution

**FIGURE 6:**
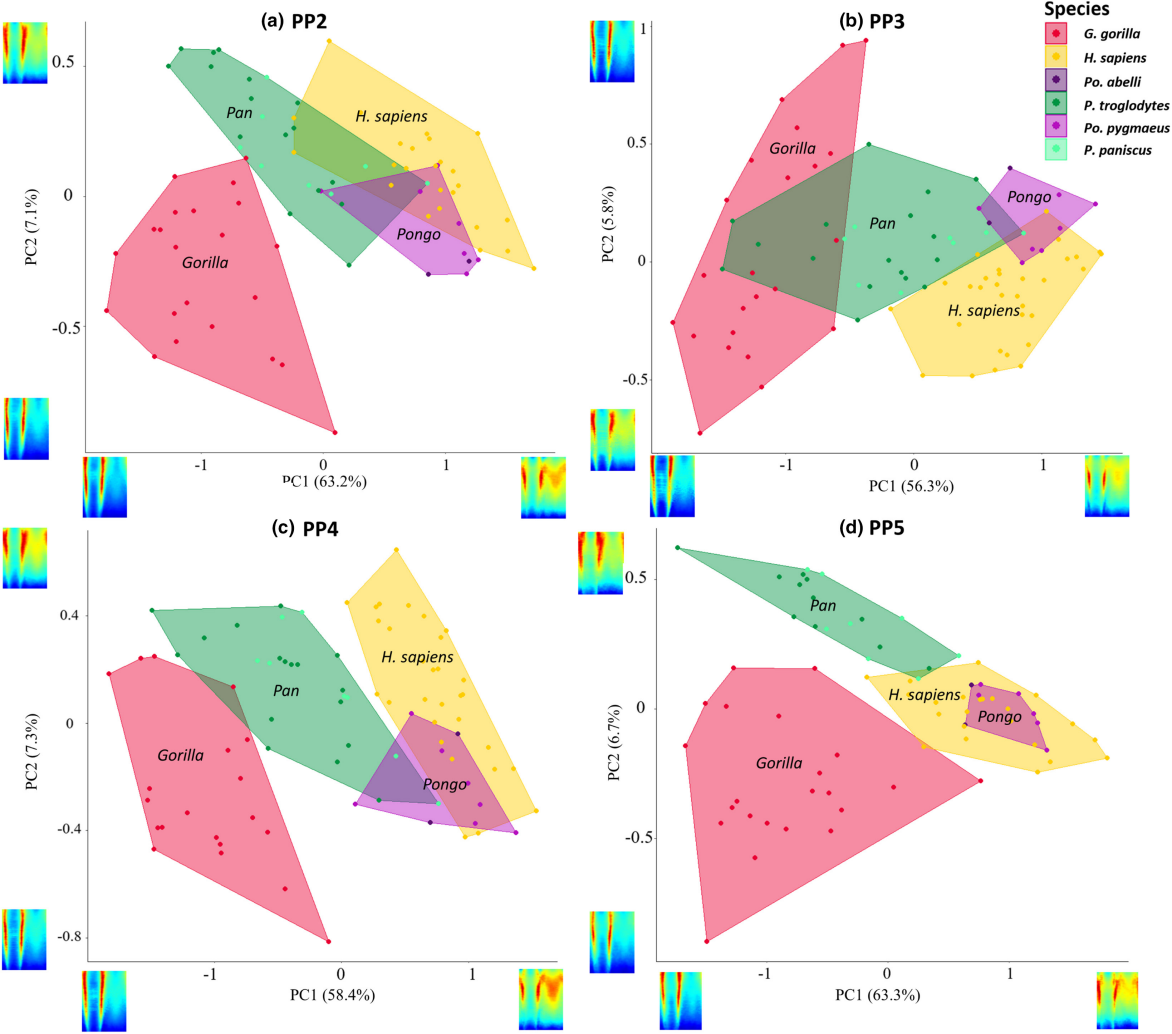
PC1 and PC2 for cortical bone distribution of proximal phalanges of (a) PP2, (b) PP3, (c) PP4 and (d) PP5 of *Homo sapiens*, *Pan* sp., *Gorilla* and *Pongo* sp.

Principal component analysis of scaled cortical thickness values from each phalanx (digits 2–5) was used to assess whether cortical thickness distribution patterns differ among taxa and whether this corresponds with their respective differences in hand use (Figures 6 and S2). PCA was conducted for each digit, however, due to comparable separation among the study taxa across all four digits, as well as similar PC1 and PC2 loadings, we describe the general pattern common to the proximal phalanges of each taxa, but highlight instances where particular digits differed from the general pattern.

PC1 explains 56% to 63% of the total variance in each of the four digits. *Gorilla* is separated from the other taxa by having low PC1 scores, representing more developed FSRs, and *H. sapiens* is characterized by high PC1 scores, reflecting a thicker distodorsal cortex in PP2–PP4. *Pan* and *Pongo* are intermediate and variably overlap with other taxa. The overlap of *Pan* and *Pongo* in PP2–PP4 may be due to the greater frequency of arboreal locomotion in *Pan* relative to *Gorilla* (Doran & Hunt, 1996; Doran,  1996; Tuttle & Watts, 1985) (Figures 6 and S2).

For PP3, low PC1 values separating *Gorilla* from other taxa are related to thickened FSRs with a low‐to‐intermediately thick dorsal region of the shaft, compared to high PC1 values in *Pongo* and *H. sapiens* reflecting distodorsal thickness and thick cortices on the FSR. The greater overlap between *Gorilla* and *Pan* in PP3 relative to the other digits is due to a few individuals of *Gorilla* displaying an intermediately thick shaft similar to *Pan*.

For PP5, low values of PC1 characterize *Gorilla* and *Pan* with thick FSRs and high values reflect distodorsal and FSR thickness in *Pongo* and *H. sapiens*. The complete overlap of *Pongo* with *H. sapiens* in PP5 is due to a distal thickening of the region under the trochlea in PP5 of both species.

PC2 explains <8% of the variance in the PCAs of all four digits and represents the region of overall maximum cortical thickness. Low values along PC2 are driven by a proximal to distal cortical bone distribution on the palmar surface and high values represent a cortical bone concentration on either the mid‐shaft to distal region of the palmar or dorsal surface of the shaft. *Gorilla* and *Pan* are the only taxa to be separated along PC2, reflecting a palmar proximo‐distal concentration of cortical bone in *Gorilla* and a mid‐shaft to distal concentration in *Gorilla* and *Pan*.

A 3D plot of PC1, PC2 and PC3 (<6%) provides clear separation among taxa, especially for PP5, with only slight overlap in *Pan* and *Pongo* in PP2 and PP4 and between *Pan*, *Pongo* and *H. sapiens* in PP3 (Figure S2).

### 3.2 Mean cortical thickness

Table 2 shows mean values of cortical thickness. Scaled mean cortical thickness values across the shaft reveal the African apes have significantly thicker cortex than *H. sapiens* and *Pongo* (Table 2; Figure S3).

### 3.3 Cross‐sectional geometry

Descriptive statistics of the scaled cross‐sectional geometric properties at 35%, 50% and 65% of the shaft are presented in Table S2 and depicted in Figures 8, 9, 10. Only *Gorilla* has significantly larger values of CA, Z_pol_ and J across all digits and cross‐sectional levels compared to the other taxa (Table S3). CSG properties differ across the digits in all taxa except *Pongo* (Table S4).

### 3.4 Pongo

3.4.1

**FIGURE 7:**
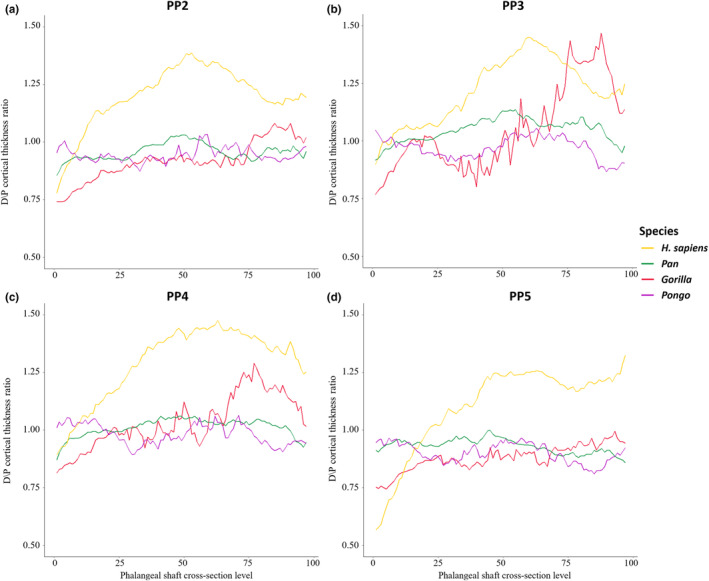
Ratio of dorsal/palmar cortical bone thickness plotted from the proximal end to the distal end of the phalangeal shaft of *Homo sapiens*, *Pan*, *Gorilla* and *Pongo*. (a) PP2; (b) PP3; (c) PP4; (d) PP5. Values greater than 1 represent more dorsal cortex relative to the palmar cortex in the shaft.

As the hand of *Pongo* is used primarily for grasping, we predicted that *Pongo* would have thicker regions of cortical bone distopalmarly on the shaft, especially close to the FSRs, and that this pattern would be consistent across the hand. In support of this prediction, we find cortical bone in *Pongo* to be thickest at the FSRs in all phalanges (Figures 4 and S1), corresponding with expected loading during grips in which the PIP joint is flexed. The point of maximum thickness within the shaft is at the distal end of the FSR, with cortical thickness reducing just distal to the FSRs and then increasing again proximal to the trochlea (Figure 5). The ratio of cortical thickness of the dorsal and palmar shaft (i.e., removing the influence of the FSRs) demonstrates that the palmar aspect of the shaft is always thicker than the dorsal (Table 3; Figure 7). A biomechanical function of FSRs is to reduce strain on the shaft, such that the taller the ridge, the more strain it experiences and consequently the amount of strain distributed to the palmar shaft is reduced (Nguyen et al., 2014). However, the FSRs in *Pongo* are not particularly prominent (i.e., do not extend far above the palmar surface of the shaft) relative to other taxa, such as *Gorilla* (Syeda et al., 2021). This suggests that the strain resulting from grasping arboreal substrates during suspension is dissipated across the FSRs, without requiring modelling of the cortical structure along the remainder of the shaft.

**TABLE 3:** Paired samples *t* tests on scaled palmar versus dorsal cortical thickness across species.

		*Homo sapiens*	*Pan*	*Gorilla*	*Pongo*
PP2	Palmar mean	0.031	0.048	0.048	0.033
	Dorsal mean	0.038	0.046	0.044	0.031
	*t* ratio	−3.489	1.057	2.363	0.904
	*p*	0.001**	NS	0.023*	NS
PP3	Palmar mean	0.029	0.042	0.043	0.030
	Dorsal mean	0.037	0.044	0.045	0.029
	*t* ratio	−4.447	−1.178	−0.945	0.516
	*p*	<0.001***	NS	NS	NS
PP4	Palmar mean	0.029	0.045	0.043	0.031
	Dorsal mean	0.038	0.045	0.044	0.030
	*t* ratio	−5.682	−0.335	−0.926	0.326
	*p*	<0.001***	NS	NS	NS
PP5	Palmar mean	0.031	0.052	0.052	0.033
	Dorsal mean	0.035	0.048	0.046	0.030
	*t* ratio	−2.149	1.583	2.940	1.791
	*p*	0.037*	NS	0.005**	NS

The bold value significance (*p* > 0.05). ^*^ p < 0.05, ^**^ p <0.01, ^***^ p < 0.001.

Abbreviation: NS, not significant.

Comparison of these patterns across the hand shows that, as we predicted, cortical bone distribution is similar across the digits in *Pongo*, with the exception of PP2, where cortical bone is thicker on the radial aspect of the palmar shaft (*Pongo* PP2 in Figure S1). This radial asymmetry could reflect grasping of very thin substrates, during which the second digit is greatly extended relative to the ulnar digits (Napier, 1960). Despite this differing pattern of cortical bone distribution in PP2, there are no significant differences in mean cortical thickness or CSG properties across the *Pongo* digits (Figure S4). The absence of significant differences in mean cortical thickness or CSG properties between the digits is consistent with relatively equal loading of all fingers during arboreal locomotion in *Pongo* (Rose, 1988; Susman, 1974; Thorpe et al., 2009; Thorpe & Crompton, 2006).

**FIGURE 8:**
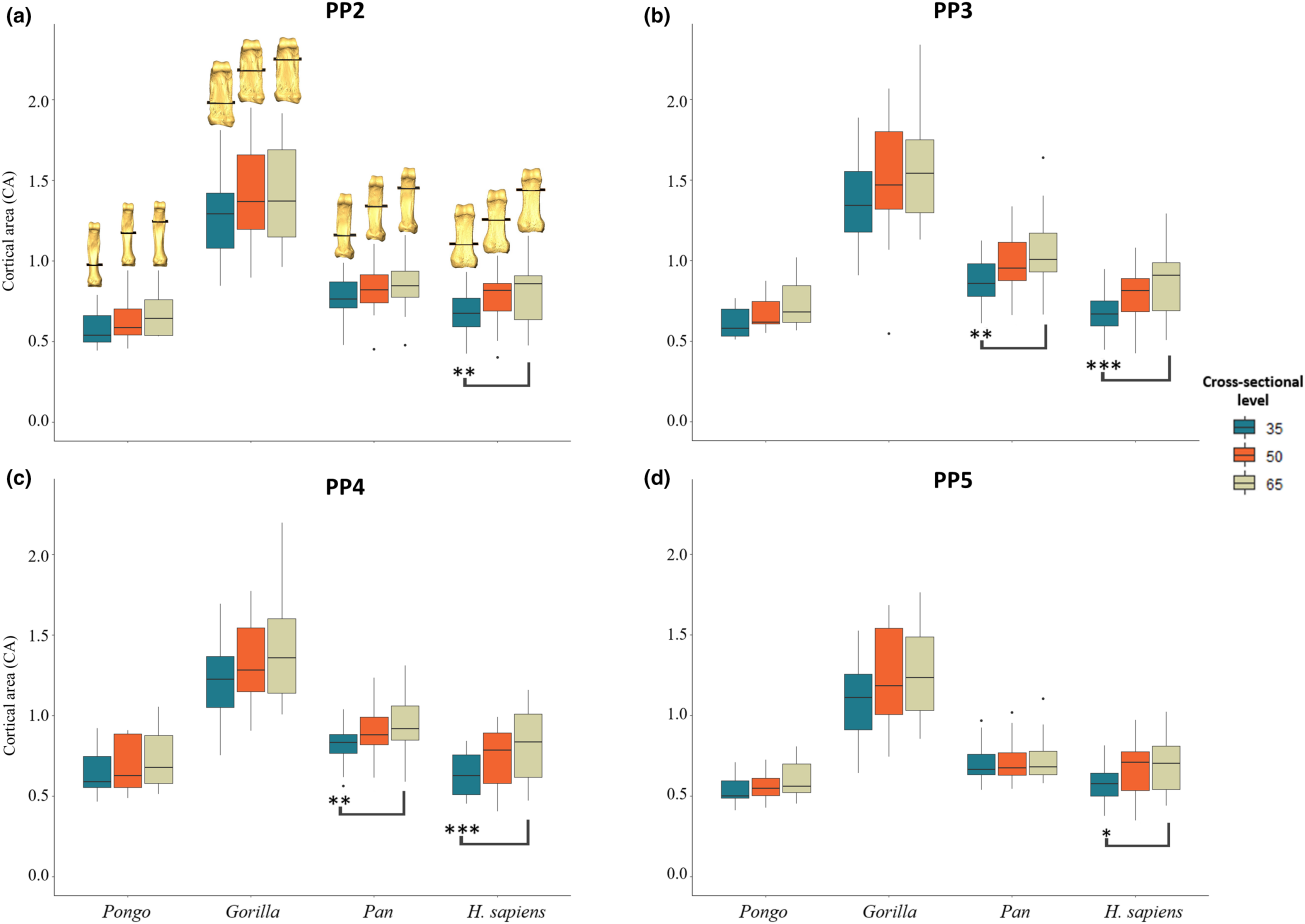
Boxplots representing cortical area for (a) PP2, (b) PP3, (c) PP4, and (d) PP5 of *Homo sapiens*, *Pan* sp., *Gorilla* and *Pongo* sp. at 35%, 50% and 65% of the bone length. Section locations are represented on 3D surfaces of PP2 of an individual from each taxon.

**FIGURE 9:**
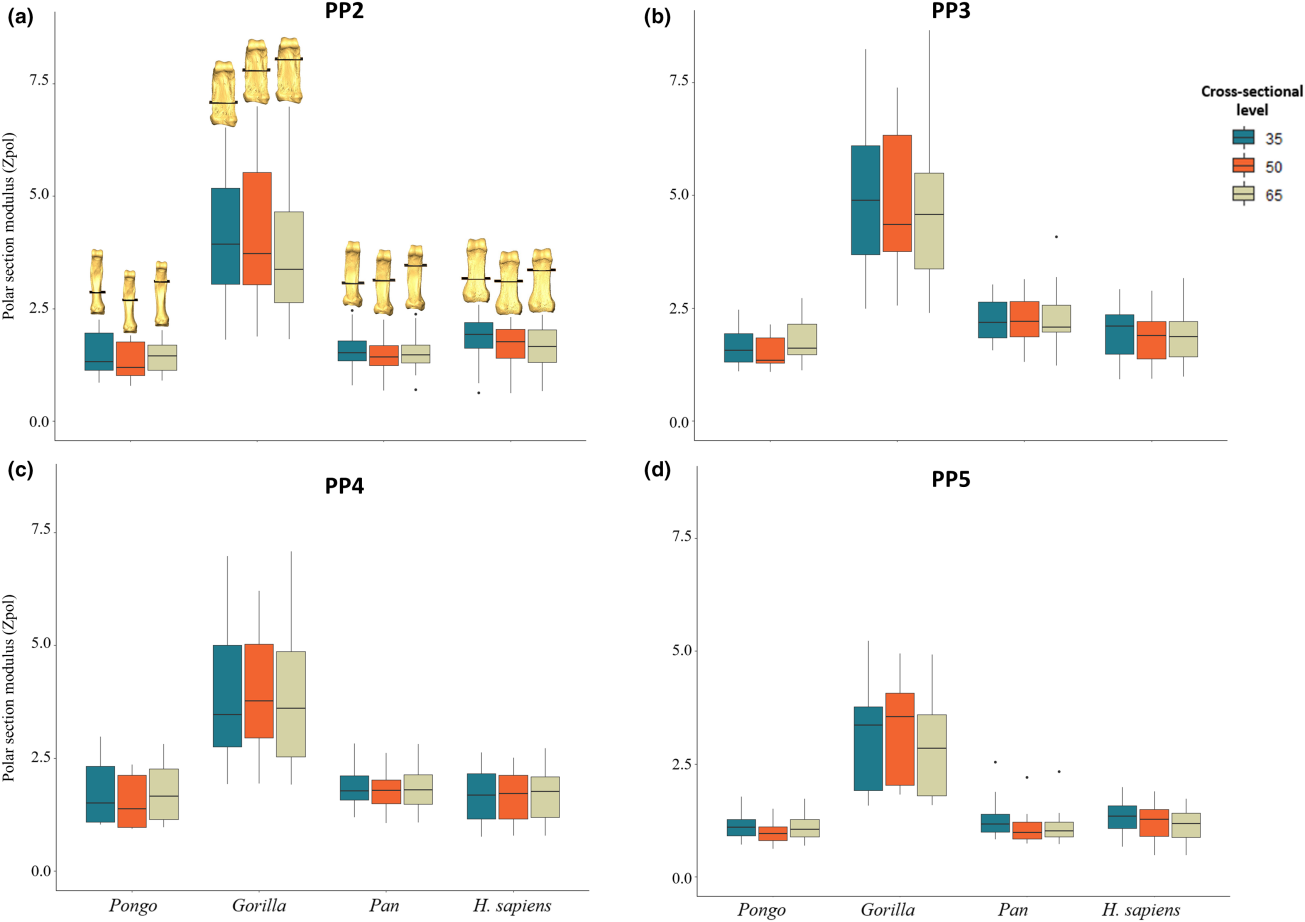
Boxplots representing polar section modulus (Z_pol_) for (a) PP2, (b) PP3, (c) PP4, and (d) PP5 of *Homo sapiens*, *Pan* sp., *Gorilla* and *Pongo* sp. at 35%, 50% and 65% of the bone length. Section locations are represented on 3D surfaces of PP2 of an individual from each taxon.

**FIGURE 10:**
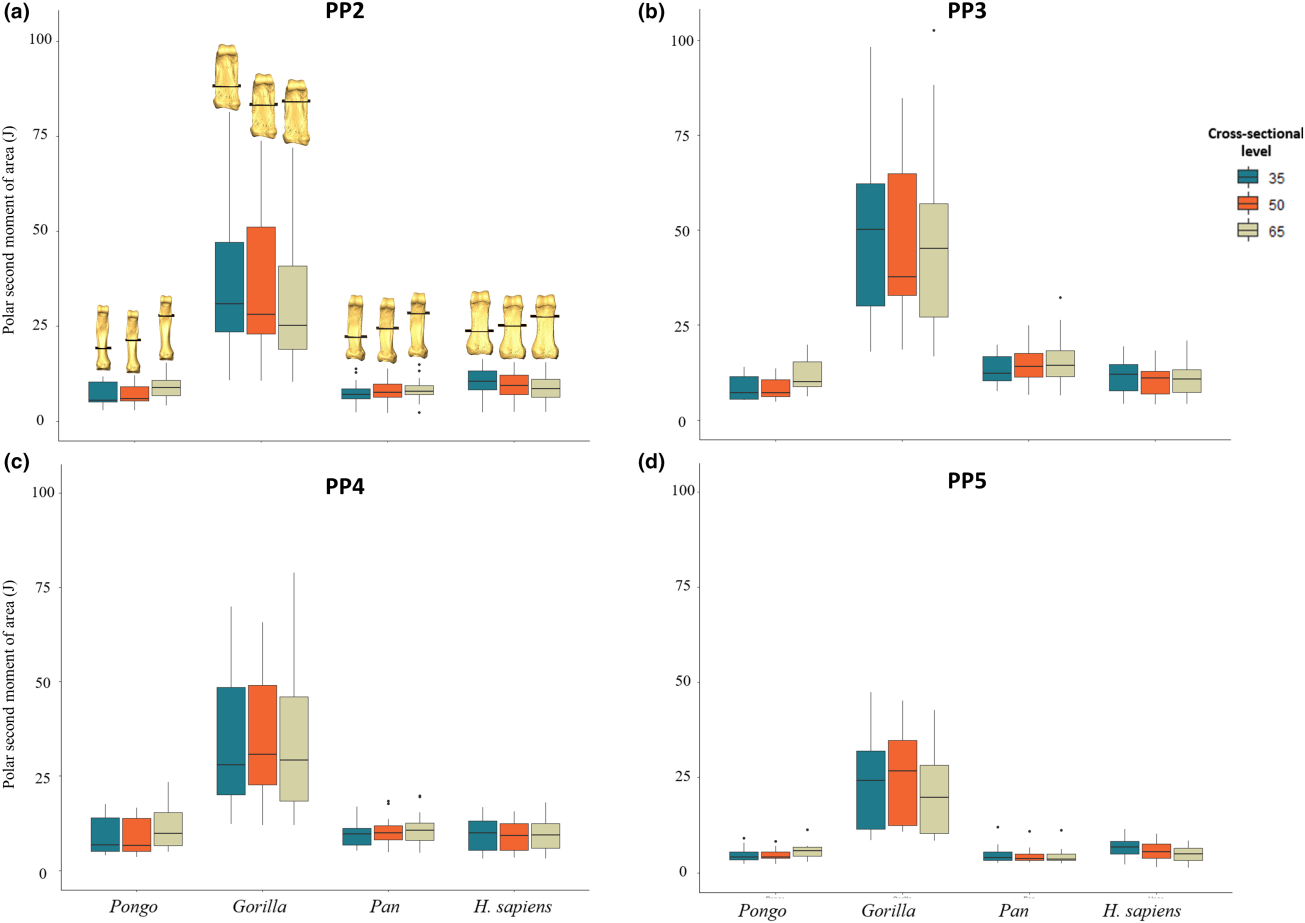
Boxplots representing polar second moment of area (J) for (a) PP2, (b) PP3, (c) PP4, and (d) PP5 of *Homo sapiens*, *Pan* sp., *Gorilla* and *Pongo* sp. at 35%, 50% and 65% of the bone length. Section locations are represented on 3D surfaces of PP2 of an individual from each taxon.

Regarding CSG properties, we predicted that *Pongo* phalanges would have thinner cortices and be less resistant to bending and torsion than those of the African apes. *Pongo* has the thinnest mean relative cortical thickness when scaled by bone length (Table 2; Figure S3), which is significantly thinner than that of African apes, partially supporting our third prediction (Figure S3). Cross‐sectional properties of *Pongo* are only significantly lower than those of *Gorilla*. However, while not significantly different from *Pan* and *H. sapiens*, relative mean values of CSG properties are lowest in *Pongo* among our sample. (Figures 8, 9, 10; Table S2). This thin cortical structure and low cross‐sectional properties of the *Pongo* proximal phalanges may relate to aspects of their external morphology. Among the great apes, *Pongo* phalanges have the greatest degree of curvature and their FSRs are located opposite the point of the maximum arc of this curvature, thus preventing the long tendons of the digital flexor muscles from being pulled into an extreme palmar position (Susman, 1979). This acts to reduce joint reaction forces and also aligns the bone more closely with this joint reaction force, ultimately leading to optimized distribution of load across the phalanx (Nguyen et al., 2014; Richmond, 2007; Susman, 1979). Thus, in *Pongo* a thicker cortex may not be needed due to the functional adaptations of the external shape to minimize strain experienced by the phalanx (Pearson & Lieberman, 2004; Ruff et al., 2006).

### 3.5 Gorilla

In support of our predictions, morphometric maps of cortical bone thickness distribution reveal the regions of thickest cortex in *Gorilla* PP2–PP4 are located in patches along the FSRs, as well as proximal to the trochlea (Figures 4 and S1). The shaft shows low‐to‐intermediate cortical thickness, with the FSRs being thicker than the remaining aspects of the shaft. Quantitative comparisons of *Gorilla* mean cortical thickness values across the shaft show a distal increase in cortical thickness in all digits (Figure 5). The distinctive regions of thicker palmar cortical bone are located at the attachment points of the soft tissues involved in stabilizing the fingers in flexed positions during knuckle‐walking. On the FSR, these locations of thicker cortical bone correspond with the attachment points of the ligaments and pulleys (Figure 4) that provide biomechanical advantage by keeping the flexor tendons close to the bone and in line with the joint axis. This decreases the moment arm and allows for optimal joint function and force transmission during finger flexion (Ayhan & Ayhan, 2020; Doyle, 2001). During knuckle‐walking, the stress in the flexor tendon is concentrated distally on the second annular pulley (A2), at the location where the tendon is maximally bent during knuckle‐walking (Leijnse et al., 2021). When the phalangeal joints are in flexion during knuckle‐walking, the flexor tendons are pulled palmarly and the digital pulleys are then stretched, which leads to increased strain in the phalanx in the same regions as we find thicker cortical bone (Ayhan & Ayhan, 2020; Leijnse et al., 2021; Ruff et al., 2006). The region of thick cortical bone proximal to the trochlea coincides with the attachment site of the collateral ligaments of the PIP joint. The collateral ligaments arise from the radial and ulnar sides of the distal end of the proximal phalanx and run obliquely to the palmar radial and ulnar surfaces of the intermediate phalanx (Figure 1f), providing lateral stability to the phalangeal joints during flexion and extension (Ayhan & Ayhan, 2020). This stability is essential for the intermediate phalanx to accommodate high loads during knuckle‐walking.

Contrary to our predictions, the pattern of cortical bone thickness distribution in PP5 is distinct from that of the more radial digits, in that the region of maximum thickness is consistently located between the proximal end of the FSR and the region just proximal to the trochlea (Figure S1). This variation in thickness may be due to lower pressure being placed on the fifth digit during knuckle‐walking compared to the other rays (Matarazzo, 2013), such that the pressure is being evenly dissipated from the proximal end of the FSRs to the distal end of the bone. The attachment points of the pulleys and ligaments may not be experiencing enough strain to elicit a biomechanical remodelling response at those regions. There is some asymmetry in the cortical thickness distribution patterns of PP2 and PP5, such that the thickest portion of the shaft in PP2 is on the palmar ulnar surface and in PP5 is on the palmar radial surface (Figure S1). This may reflect the location of pressures experienced during knuckle‐walking, which are highest on the third digit (Matarazzo, 2013; Preuschoft, 1973; Samuel et al., 2018).

Furthermore, there is variation in the patterning of palmar and dorsal cortical thickness in the proximal phalanges of *Gorilla*. There is no significant difference in thickness between the palmar and dorsal cortex of PP3 and PP4, but in PP2 (*p* = 0.023) and PP5 (*p* = 0.005) the cortex is significantly thicker palmarly compared to dorsally (Table 3). This could be due to the smaller FSRs of PP2 and PP5 compared to PP3 and PP4, in which the strain on the palmar shaft is reduced due to the tall FSRs (Nguyen et al., 2014; Susman, 1979). While there are nuanced differences in each of the digits in regard to cortical bone distribution pattern and relative palmar and dorsal cortical thickness, we predicted no overall differences in mean cortical thickness and cross‐sectional properties across the *Gorilla* digits. However, PP5 has significantly lower CSG than PP3 (Tables S2 and S4). These results could be due to more neutral position of the *Gorilla* hand during the majority of knuckle‐walking hand postures, along with similar lengths of the metacarpus and proximal phalanges, which allows them to consistently touchdown with their fifth digit despite placing significantly less pressure on it relative to the other digits (Matarazzo, 2013; Susman, 1979; Susman & Stern, 1979; Thompson et al., 2018). However, it is important to acknowledge the studies that quantified pressure distribution during locomotion in extant non‐human great apes (e.g., Matarazzo, 2013; Samuel et al., 2018; Wunderlich & Jungers, 2009) have, for logistical reasons, focused on animals in captivity in an enclosed space and likely do not fully reflect manual behaviours in the wild.

### 3.6 Pan

Our expectations for *Pan* were generally supported. The pattern of cortical bone distribution in *Pan* is similar to *Gorilla* in having thicker cortical bone at the FSRs and in the region proximal to the trochlea. However, unlike *Gorilla*, the shaft is relatively intermediate in its thickness compared to the thin proximal region of the bone (Figures 4 and S1). This difference in cortical bone thickness patterning among the knuckle‐walking apes could be a reflection of *Pan* participating in arboreal behaviours to a greater extent than *Gorilla* (Doran, 1996, 1997; Hunt, 2020; MacKinnon, 1976; Sarringhaus et al., 2014; Susman, 1984). While the magnitude of loads during knuckle‐walking and arboreal locomotion have been shown to be similar (Synek et al., 2020), loads of knuckle‐walking may be reflected in the internal morphology more so than the overall forces of infrequent arboreal behaviours. External morphological features may play a role in these differences in internal bone structure. Within the African apes, the higher degree of curvature of the *Pan* phalanges, relative to that of *Gorilla*, should be an advantage for load distribution during arboreal behaviours (Deane & Begun, 2008; Hunt, 1991; Oxnard, 1973; Richmond, 2007; Stern et al., 1995), but the less prominent FSRs would not act to reduce strain experienced by the remainder of the shaft to the same extent as in *Gorilla* (Nguyen et al., 2014). As such, CSG properties, mean cortical bone thickness and distribution patterns may reflect the greater degree of arboreal behaviours in *Pan*.

Our prediction that there will be variation in cortical thickness pattern and properties across the *Pan* digits was not fully supported. Unexpectedly, PP5 has significantly thicker cortex (*p* = 0.044; Figure S4) than PP3, but when compared to PP5, the radial three digits are significantly stronger in resisting axial, bending and torsional loads, along with PP3 being stronger than PP2 (Tables S2 and S4). Overall, these results may reflect low loading of the fifth digit during knuckle‐walking, as it is loaded significantly less than the other digits and sometimes does not make contact with the substrate (Matarazzo, 2013; Wunderlich & Jungers, 2009). While surprising, the relatively thinner cortex in PP3 may be reflecting the impact of external morphology (taller FSRs, high degree of curvature), which are most prominent in the third digit within the *Pan* hand, on cortical remodelling. The similarity in cortical properties among the radial digits could be explained by the variability of hand postures used by *Pan* (Inouye, 1994; Matarazzo, 2013; Samuel et al., 2018; Tuttle, 1967, 1969; Wunderlich & Jungers, 2009), such that the varying hand positions during locomotion result in differing sequences of digital placement, affecting which digit receives the greatest pressures (Wunderlich & Jungers, 2009). The variation in knuckle‐walking hand postures and greater degree of arboreality in the *Pan* locomotor repertoire, may also explain the intermediate thickness of the shaft with no significant difference in palmar and dorsal cortical thickness (Figure 4; Table 3). PP5 is also distinct from the other digits in displaying a radial concentration in its thickness pattern (Figure S1), potentially reflecting peak pressures during locomotion being located around the centre of the hand and lower pressures under the fifth digit (Matarazzo, 2013; Preuschoft, 1973; Samuel et al., 2018).

### 3.7 H. sapiens

Our predictions that *H. sapiens* would display the thickest cortex in the distodorsal region of the shaft and that they would be characterized by thick cortical bone where FSRs are present, are generally supported (Figures 4 and S1). Although the distal dorsal and palmar aspects of the phalangeal shaft are thick as predicted, cortical thickness is concentrated on the mid‐shaft to distodorsal region of the diaphysis. Cortical thickness of the dorsal surface is significantly greater than the palmar surface (Figure 7; Table 3) and decreases past the distodorsal region of maximum cortical thickness (Figure 5). This could reflect the lack of phalangeal curvature in *H. sapiens* and the frequent use of flexed hand postures during modern human manipulation. Hand grips used during manipulation result in bending forces being placed on the phalanges, with the dorsal surface on the bone experiencing higher tensile forces and the palmar surface experiencing compression, and the lack of curvature characteristic of *H. sapiens* phalanges results in higher bending forces experienced by the bone overall (Oxnard, 1973; Preuschoft, 1973; Richmond, 2007).

Across the digits, we predicted PP2 and PP3 would display the thickest cortices and greatest cross‐sectional strength, as experimental studies have revealed that the thumb and radial digits experience the highest loads during manipulation (Key, 2016; Rolian et al., 2011; Williams‐Hatala et al., 2018). Furthermore, experimental studies testing force distribution of power grips used in modern human daily activities have revealed that, within digits 2–5, digit 2 experiences the greatest loads and the three ulnar digits experience relatively equal loads when grasping larger objects (De Monsabert et al., 2012; Sancho‐Bru et al., 2014; Vigouroux et al., 2011). In contrast, loading of the digits is variable when grasping objects with a smaller diameter (<6.4 cm), as positioning of the fingers can be adjusted to maximize endurance without losing hold of the object (Sancho‐Bru et al., 2014). Mean cortical thickness and cross‐sectional properties are greatest in PP3, followed by PP2, PP4 and PP5, but there were no significant differences in cortical thickness across the digits (Table 2; Figure S4). Only PP5 was significantly lower in its measure of axial strength (CA), bending strength (Z_pol_) and bending and torsional rigidity (J) (Tables S2 and S4; Figures 8, 9, 10). As our sample includes a diverse range of pre‐ and post‐industrial populations, our results could simply reflect the varied hand postures employed during the daily activities of individuals from these populations, and not necessarily correspond with those employed during stone tool production (see Key et al., 2019).

### 3.8 Phalangeal curvature and cortical thickness

The regression analyses showed no relationship between the degree of curvature (IA) and phalangeal cortical thickness in *Pongo*, *Gorilla* and *H. sapiens* (Figure S8). There was a significant (*p* = 0.001), but weak (*R* ^2^ = 0.106) positive correlation between curvature and cortical thickness in *Pan* proximal phalanges (Table S5). Our results suggest a weak relationship between phalangeal curvature and cortical thickness, despite a curved phalanx having been shown to dissipate load differently than a straight phalanx (Oxnard, 1973; Preuschoft, 1973). These results may also reflect the lack of precision offered by the IA method, which assumes a consistent degree of curvature throughout the phalanx (see Deane & Begun, 2008; Wennemann et al., 2022).

### 3.9 Behavioural signals in the cortex of the proximal phalanges

Great apes use their hands in distinct ways and adopt variable hand postures to accomplish a wide range of locomotor and/or manipulative tasks. Aspects of their external hand bone morphology aid them in successfully participating in these manual behaviours, with associated modelling of internal cortical and trabecular bone morphology (Bird et al., 2022; Dunmore et al., 2019; Kivell, 2015; Marchi, 2005; Matarazzo, 2008; Nguyen et al., 2014; Tsegai et al., 2013). Here, we demonstrate that cortical bone in the proximal phalanges reflects differences in hand use behaviours and external morphology.

While cortical bone properties and distribution patterns differed across the great apes, the functional role of FSRs is clear across all taxa. Within the non‐human great apes, the location of maximum cortical thickness always includes the FSRs and in human individuals, where FSRs are present, they are maximally thick as well (Figures 4 and S1). These results, coupled with the pattern in *Gorilla* where phalanges with less prominent FSRs (PP2 and PP5) have thicker palmar cortex than dorsal cortex, while phalanges with more prominent FSRs (PP3 and PP4) show no differences, further suggests that prominent FSRs reduce strain experienced by the palmar shaft (Nguyen et al., 2014). This is also apparent in the cortical thickness distribution pattern of *Pongo* phalanges, where though FSRs are the thickest region of the shaft, the shaft is also intermediately thick because *Pongo* FSRs are not very prominent. While *Pongo* FSRs are small, they are optimally located to resist forces during flexion and are coupled with high phalangeal curvature (Patel & Maiolino, 2016; Susman, 1979; Syeda et al., 2021), such that the external morphology of *Pongo* phalanges and cortical bone distribution pattern may be optimal for the manual loads they experience during flexed finger grasping. We draw this conclusion based on the fact that *Pongo* phalanges have thin cortices and weak cross‐sectional properties relative to the other great apes, suggesting that a mechanical modelling response for a thicker cortex might not be needed (Pearson & Lieberman, 2004).

*Gorilla* and *Pan* have a similar locomotor repertoire (Doran, 1996; Matarazzo, 2013; Samuel et al., 2018; Wunderlich & Jungers, 2009), which is reflected in the cortical bone morphology of their proximal phalanges. Specifically, a shared pattern of thick cortex at the FSRs and in the distal region under the trochlea in *Gorilla* and *Pan* is indicative of the loading pattern incurred during knuckle‐walking (Matarazzo, 2013; Samuel et al., 2018; Wunderlich & Jungers, 2009). Even though loads experienced by the metacarpals, and possibly the proximal phalanges, during knuckle‐walking and arboreal behaviours are similar (Synek et al., 2020), the frequency of knuckle walking is greater (e.g., Doran, 1996, 1997; Hunt, 1991). We assume, therefore, that the cortical patterns we found primarily reflect knuckle‐walking, and this is supported by variation in external and internal morphology between African apes and *Pongo*. However, it is important to acknowledge that infrequent behaviours can also result in bone (re‐) modelling (Barak et al., 2011; Burr, 1990; Pontzer et al., 2006). For example, the digital flexor muscles are minimally active during knuckle‐walking but highly active during arboreal climbing and suspension (Leijnse et al., 2021; Susman & Stern, 1979; Thompson et al., 2019; Tuttle et al., 1972), and thus arboreal behaviours are likely contribute to some of the patterns we observe in *Gorilla* and *Pan* proximal phalanges. As for differences, the variation in hand morphology and postures employed by the two species during locomotion likely leads to differences in the pattern of loading across the non‐pollical digits, and this is also reflected in our results (Inouye, 1994; Tuttle, 1969).

The distinct dorsal thickening of human phalanges is expected for phalanges that are relatively straight and are consistently loaded in a flexed position. We predicted that cortical structure of PP2 and PP3 would reflect their more frequent use during daily manipulative behaviours but instead found a consistent pattern across the digits. This could reflect use of a diverse set of precision and power grips by modern humans (Dollar, 2014; Feix et al., 2015; Sancho‐Bru et al., 2014). Furthermore, it is important to acknowledge that studies of recent modern human (often industrialized, Western populations) daily hand use are likely not representative of daily hand use in our geographically and temporally diverse sample. However, PP5 was significantly weaker and had a thinner cortex than the remaining three digits across our sample, which could reflect a general pattern of more limited recruitment of the fifth digit during habitual manual activities (but see Key et al., 2019; Marzke, 1997).

Evaluating bone strength using cross‐sectional properties plotted across the shaft showed a distinct pattern in non‐human great apes (Figures S5–S7). Specifically, the proximal phalangeal shaft exhibits a CA that is generally greatest on the distal end of the bone, while the rigidity and resistance to torsion are greatest on the proximal end (Figures 8 and S5–S7; Tables S2). This pattern may reflect the disto‐proximal transfer of load across the digit, such that the proximal aspect of the bone needs to be structurally adapted to resist greater loads (Matarazzo, 2015).

While our results support the conclusion that phalangeal cortical bone structure reflects differences in manual behaviours in extant great apes, these interpretations rely on predictions of loading patterns and force transfer that are dependent on the function of muscles, ligaments and other soft tissue structures, about which we know very little. Furthermore, we chose to scale our cortical bone measures by the length of the proximal phalanx, but there are fundamental differences in hand proportions across the great apes (Patel & Maiolino, 2016) that do not show a direct relationship to body mass, and thus a different scaling factor might produce different relative patterns. We tested this potential difference by scaling our data by a geometric mean of phalangeal length, mid‐shaft breadth, breadth of the base and breadth of the trochlea, which reflect proximal phalanx size, but found a similar pattern to scaling with phalangeal bone length. Detailed behavioural and kinematic studies on various manual behaviours used by great apes, ideally in natural environments, together with musculoskeletal modelling and cadaveric validation are required (e.g., Leijnse et al., 2021; Lu et al., 2018; Synek et al., 2020). In addition, further investigation of ontogenetic changes in both external morphology (e.g., phalangeal curvature, entheseal morphology) and internal bone structure would also provide insight into the functional interplay between bone shape and bone modelling.

## 4 CONCLUSIONS

While, among great apes, cortical bone thickness patterns generally reflect the predicted loading regimes of different locomotor and manual behaviours, more nuanced information about loading during varying hand postures is evident from patterns of cortical bone distribution and cross‐sectional properties. Cortical bone and its cross‐sectional parameters reflected not just hand postural differences, but also the differences within the hand of each great ape species. More research is needed on phalangeal external and internal forms, however, this study has demonstrated that cortical bone of proximal phalanges of digits 2–5 holds functional signals of hand use and thus, the cortex of proximal phalanges has the potential to aid in reconstruction of manual behaviours of fossil hominids, including hominins.

## Author contributions

SMS conceived and designed the experiments, acquire data, analysed and interpreted the data, prepared figures and tables, authored the first draft and reviewed subsequential drafts of the paper, and approved of the final draft. ZJT and MC provided tools for data analysis, provided critical revision of the manuscript, and approved of the final manuscript. TLK and MMS conceived and designed the experiments, contributed data, assisted with the interpretation of the data, provided critical revision of the manuscript, and approved the final manuscript.

## Supporting information


**Supporting information S1.** Supplementary material

## Data Availability

Copies of all scans are curated by the relevant curatorial institutions that are responsible for the original specimens and access can be requested through each institution. The authors confirm that the data supporting the findings of this study are available from the corresponding author upon reasonable request.
