# Supplementary material for: Cortical bone distribution of the proximal phalanges in great apes: implications for reconstructing manual behaviours
Source: J Anat. 2023 Jun 26;243(5):707–28. doi: 10.1111/joa.13918 (PMC10557399; doi:10.1111/joa.13918)
Supplement: Supplementary file 1 — Supporting information S1. Supplementary material [file JOA-243-707-s001.pdf]

SUPPLEMENTARY MATERIALS

Cortical bone distribution of the proximal phalanges in great apes and implications for reconstructing manual behaviours

Samar M. Syeda[1], Zewdi J. Tsegai[1,2], Marine Cazenave[3,1,4], Matthew M. Skinner[1,5], Tracy L. Kivell[1,5]

Supplementary Information 1: Siding phalanges

For the majority of our sample (65%), we include associated PP2-PP5 from a single hand (right or left, depending on which was most complete) of one individual. However, in instances when this was not possible due to preservation or methodological issues, proximal phalanges from the other hand in the same individual or individuals with an incomplete set of proximal phalanges were also included in the sample. For incomplete sets of phalanges, we assigned phalanges to a digit for the non-human great apes following Susman (1979) and Patel & Maiolino (2016). For humans, whose PP2 and PP4 are not as morphologically distinct as those of non-human great apes, we used minor variations in basal morphology to identify them: PP2 has an enlarged radial tubercle to accommodate the first dorsal interossei muscle, while the PP4 base is more symmetrical with a palmarly projecting radial tubercle (Susman, 1979; Case & Heilman, 2006).

Supplementary tables and figures: Below are supplementary tables and figures that expand on the results presented in the manuscript.

Supplementary Table 1: Detailed specimen information.

Taxon	Specimen	Sex	Side	Subsistence	Collection
Homo sapiens	NHMW-Nubian_J2	M	R	Pre-industrial	Natural History Museum, Vienna
Homo sapiens	NHMW-Nubian_K63	M	L	Pre-industrial	Natural History Museum, Vienna
Homo sapiens	NHMW-Nubian_K5.2	M	R	Pre-industrial	Natural History Museum, Vienna
Homo sapiens	NHMW-Nubian_J7	F	R	Pre-industrial	Natural History Museum, Vienna
Homo sapiens	Qafzeh_9	M	R	Pre-industrial	The Sackler School of Medicine at Tel Aviv University
Homo sapiens	Qafzeh_8	UK	R/L	Pre-industrial	The Sackler School of Medicine at Tel Aviv University
Homo sapiens	OHALO_II_H2	M	R/L	Pre-industrial	The Sackler School of Medicine at Tel Aviv University
Homo sapiens	UNIFL_4865	M	R	Post-industrial	University of Florence
Homo sapiens	UNIFL_4887	F	R	Post-industrial	University of Florence
Homo sapiens	UNIFL_3124	UK	UK	Pre-industrial	University of Florence
Homo sapiens	UNIFL_3125	UK	UK	Pre-industrial	University of Florence
Homo sapiens	UNIFL_3127	M	L	Pre-industrial	University of Florence
Homo sapiens	GAUG-Inden_91	M	R	Post-industrial	Georg-August-University Goettingen, Anthropology Collection
Homo sapiens	GAUG-Inden_113	M	L	Post-industrial	Georg-August-University Goettingen, Anthropology Collection
Homo sapiens	GAUG-Inden_117	UK	L	Post-industrial	Georg-August-University Goettingen, Anthropology Collection
Homo sapiens	GAUG-Inden_119	M	R	Post-industrial	Georg-August-University Goettingen, Anthropology Collection
Homo sapiens	GAUG-Inden_243	M	L	Post-industrial	Georg-August-University Goettingen, Anthropology Collection
Homo sapiens	Barma_Grande_2	M	R	Pre-industrial	Museo Nazionale Preistorico dei Balzi Rossi
Homo sapiens	ARENE_CANDIDE_2	M	R/L	Pre-industrial	the Museo Archeologico del Finale
Homo sapiens	DCW_AM_3_0_2	UK	R/L	Pre-industrial	The Duckworth Collection, University of Cambridge
Homo sapiens	DCW_OC_1_0_141	UK	R/L	Pre-industrial	The Duckworth Collection, University of Cambridge
Homo sapiens	DCW_OC_1_0_26	UK	L	Pre-industrial	The Duckworth Collection, University of Cambridge
Homo sapiens	FCS8	M	R/L	Post-industrial	Mary Rose Trust
Homo sapiens	81-H1035	M	R	Post-industrial	Mary Rose Trust
Homo sapiens	81-H1040	M	R	Post-industrial	Mary Rose Trust
Homo sapiens	81-H1068-DD	M	R	Post-industrial	Mary Rose Trust
Homo sapiens	FCS16	M	R/L	Post-industrial	Mary Rose Trust
Homo sapiens	FCS17	M	R/L	Post-industrial	Mary Rose Trust
Homo sapiens	81-H172-H	M	R/L	Post-industrial	Mary Rose Trust
Homo sapiens	DV-13	M	L	Pre-industrial	Institute of Archaeology, Czech Republic

Homo sapiens	DV-14	M	R	Pre-industrial	Institute of Archaeology, Czech Republic
Homo sapiens	DV-15	M	R/L	Pre-industrial	Institute of Archaeology, Czech Republic
Homo sapiens	DV-16	M	R/L	Pre-industrial	Institute of Archaeology, Czech Republic
Pan paniscus	MRAC_27698	F	L	Wild	Royal Museum for Central Africa, Tervuren
Pan paniscus	MRAC_29042	F	R	Wild	Royal Museum for Central Africa, Tervuren
Pan paniscus	MRAC_29045	F	L	Wild	Royal Museum for Central Africa, Tervuren
Pan paniscus	MRAC_29052	M	R	Wild	Royal Museum for Central Africa, Tervuren
Pan paniscus	MRAC_27696	M	L	Wild	Royal Museum for Central Africa, Tervuren
Pan paniscus	MRAC_20881	M	L	Wild	Royal Museum for Central Africa, Tervuren
Pan paniscus	MRAC_29060	F	R	Wild	Royal Museum for Central Africa, Tervuren
Pan troglodytes	MPITC_11778	F	R	Wild	Max Planck Institute for Evolutionary Anthropology, Taï Chimp Collection
Pan troglodytes	MPITC_14996	F	L	Wild	Max Planck Institute for Evolutionary Anthropology, Taï Chimp Collection
Pan troglodytes	NH_CAM1_204	F	L	Wild	Powell-Cotton Museum
Pan troglodytes	NH_CAM2_301	F	R	Wild	Powell-Cotton Museum
Pan troglodytes	NH_MER_279	F	L	Wild	Powell-Cotton Museum
Pan troglodytes	NH_MER35_86	F	L	Wild	Powell-Cotton Museum
Pan troglodytes	NH_MER35_105	F	R	Wild	Powell-Cotton Museum
Pan troglodytes	MPITC_11903	M	L	Wild	Max Planck Institute for Evolutionary Anthropology, Taï Chimp Collection
Pan troglodytes	MPITC_11789	M	L	Wild	Max Planck Institute for Evolutionary Anthropology, Taï Chimp Collection
Pan troglodytes	MPITC_11781	M	L	Wild	Max Planck Institute for Evolutionary Anthropology, Taï Chimp Collection
Pan troglodytes	ZSM_AP-122	M	R	Wild	Bavarian State Collection of Zoology
Pan troglodytes	PC_ZVII_24	M	R	Wild	Powell-Cotton Museum
Pan troglodytes	NH_MER33_712	M	R	Wild	Powell-Cotton Museum
Pan troglodytes	NH_MER33_724	M	L	Wild	Powell-Cotton Museum
Pan troglodytes	NH_MER32_401	M	L	Wild	Powell-Cotton Museum
Pan troglodytes	NH_MER33_440	M	R	Wild	Powell-Cotton Museum
Pan troglodytes	NH_MER36_254	M	L	Wild	Powell-Cotton Museum
Gorilla gorilla	ZMB_11642	UK	R	Wild	Natural History Museum, Berlin
Gorilla gorilla	ZMB_83545	M	R	Wild	Natural History Museum, Berlin
Gorilla gorilla	PC_MER_95	F	R	Wild	Powell-Cotton Museum
Gorilla gorilla	PC_MER_135	M	L	Wild	Powell-Cotton Museum
Gorilla gorilla	PC_MER_264	M	L	Wild	Powell-Cotton Museum
Gorilla gorilla	PC_MER_300	F	R	Wild	Powell-Cotton Museum

Gorilla gorilla	PC_MER_372	M	R	Wild	Powell-Cotton Museum
Gorilla gorilla	PC_MER_962	M	R	Wild	Powell-Cotton Museum
Gorilla gorilla	PC_MER1_29	F	R	Wild	Powell-Cotton Museum
Gorilla gorilla	PC_MER_138	F	L	Wild	Powell-Cotton Museum
Gorilla gorilla	PC_MER_174	F	R	Wild	Powell-Cotton Museum
Gorilla gorilla	PC_MER_696	F	R	Wild	Powell-Cotton Museum
Gorilla gorilla	PC_MER_856	F	L	Wild	Powell-Cotton Museum
Gorilla gorilla	PC_ZII_64	M	R	Wild	Powell-Cotton Museum
Gorilla gorilla	PC_ZVI_32	M	R	Wild	Powell-Cotton Museum
Gorilla gorilla	NH_MER33_755	F	R	Wild	Powell-Cotton Museum
Gorilla gorilla	NH_MER33_461	M	R	Wild	Powell-Cotton Museum
Gorilla gorilla	NH_CAM1_106	M	R	Wild	Powell-Cotton Museum
Gorilla gorilla	NH_CAM1_105	M	R	Wild	Powell-Cotton Museum
Gorilla gorilla	NH_MER35_150	F	R	Wild	Powell-Cotton Museum
Gorilla gorilla	NH_CAM1_98	F	L	Wild	Powell-Cotton Museum
Gorilla gorilla	NH_MER35_136	F	L	Wild	Powell-Cotton Museum
Gorilla gorilla	NH_MER35_139	F	L	Wild	Powell-Cotton Museum
Gorilla gorilla	NH_FC_130	M	L	Wild	Powell-Cotton Museum
Gorilla gorilla	NH_FC_123	M	L	Wild	Powell-Cotton Museum
Pongo abelii	SMF_6785	M	R	Wild	Senckenberg Natural History Museum, Frankfurt
Pongo abelii	SMF_6779	F	L	Wild	Senckenberg Natural History Museum, Frankfurt
Pongo pygmaeus	ZSM_1907_0633b	F	R	Wild	Bavarian State Collection of Zoology
Pongo pygmaeus	ZSM_1907_0629b	M	R	Wild	Bavarian State Collection of Zoology
Pongo pygmaeus	ZSM_1907_0660	F	R	Wild	Bavarian State Collection of Zoology
Pongo pygmaeus	ZSM_AP-120	M	L	Wild	Bavarian State Collection of Zoology
Pongo pygmaeus	ZSM_1907_0483	F	R	Wild	Bavarian State Collection of Zoology
Pongo pygmaeus	ZSM_1909_0801	M	R	Wild	Bavarian State Collection of Zoology
Pongo pygmaeus	ZMB_87092	F	R	Wild	Natural History Museum, Berlin

Note: M = male; F = female; R = right; L = left; UK = unknown sex/side; R/L = to have all four digits represented, some phalanges were from the right side of the body and some from the left.

Supplementary Table 2: Mean values of standardised cross-sectional properties across species at 35%, 50%, and 65% of the phalanx

		H. sapiens	*Pan*	*Gorilla*	*Pongo*
**35% cross-section**					
PP2	CA (mm^2)	0.677	0.780	1.285	0.592
	Z_{pol} (mm^3)	1.815	1.559	4.214	1.462
	J (mm^4)	10.380	7.459	37.262	6.864
PP3	CA (mm^2)	0.679	0.876	1.366	0.612
	Z_{pol} (mm^3)	1.975	2.257	4.950	1.669
	J (mm^4)	11.606	13.102	48.787	8.446
PP4	CA (mm^2)	0.639	0.819	1.227	0.629
	Z_{pol} (mm^3)	1.672	1.857	4.003	1.742
	J (mm^4)	9.549	9.564	36.234	9.119
PP5	CSA (mm^2)	0.566	0.695	1.105	0.541
	Z_{pol} (mm^3)	1.314	1.265	2.986	1.152
	J (mm^4)	6.613	4.751	22.827	4.888
**50% cross-section**					
PP2	CA (mm^2)	0.767	0.832	1.412	0.636
	Z_{pol} (mm^3)	1.678	1.456	4.317	1.302
	J (mm^4)	9.092	7.894	37.632	6.901
PP3	CA (mm^2)	0.779	0.978	1.507	0.663
	Z_{pol} (mm^3)	1.838	2.196	5.018	1.496
	J (mm^4)	10.479	14.420	49.767	8.330
PP4	CA (mm^2)	0.750	0.893	1.315	0.672
	Z_{pol} (mm^3)	1.637	1.740	4.020	1.529
	J (mm^4)	9.076	10.237	36.357	8.800
PP5	CA (mm^2)	0.664	0.711	1.246	0.567
	Z_{pol} (mm^3)	1.207	1.067	3.178	1.000
	J (mm^4)	5.683	4.509	24.766	4.805
**65% cross-section**					
PP2	CA (mm^2)	0.817	0.860	1.417	0.677
	Z_{pol} (mm^3)	1.626	1.514	3.724	1.424
	J (mm^4)	8.625	8.158	31.730	8.846
PP3	CA (mm^2)	0.854	1.031	1.563	0.732
	Z_{pol} (mm^3)	1.826	2.277	4.635	1.732
	J (mm^4)	10.507	15.152	45.513	11.540
PP4	CA (mm^2)	0.823	0.940	1.389	0.736
	Z_{pol} (mm^3)	1.675	1.847	3.733	1.740
	J (mm^4)	9.373	10.891	32.763	11.794
PP5	CA (mm^2)	0.688	0.719	1.263	0.602
	Z_{pol} (mm^3)	1.121	1.096	2.821	1.106
	J (mm^4)	4.882	4.355	20.961	6.031

Abbreviations: CA = cortical area; Z_{pol} = polar section modulus; J = polar second moment of area.

Supplementary Table 3: Significance values for post hoc comparisons of cross-sectional properties across species at 35%, 50%, and 65% of the phalanx.

			35%			50%			65%		
			Pan	*Gorilla*	*Pongo*	*Pan*	*Gorilla*	*Pongo*	*Pan*	*Gorilla*	*Pongo*
PP2	CA	*H.sapiens*	NS	**<0.001**	NS	NS	**<0.001**	NS	NS	**<0.001**	NS
		Pan		**<0.001**	NS		**<0.001**	NS		**<0.001**	NS
		Gorilla			**<0.001**			**<0.001**			**<0.001**
	Z_{pol}	*H.sapiens*	NS	**<0.001**	NS	NS	**<0.001**	NS	NS	**<0.001**	NS
		Pan		**<0.001**	NS		**<0.001**	NS		**<0.001**	NS
		Gorilla			**<0.001**			**<0.001**			**<0.001**
	J	*H.sapiens*	NS	**<0.001**	NS	NS	**<0.001**	NS	NS	**<0.001**	NS
		Pan		**<0.001**	NS		**<0.001**	NS		**<0.001**	NS
		Gorilla			**<0.001**			**<0.001**			**<0.001**
PP3	CA	*H.sapiens*	**0.004**	**<0.001**	NS	**0.029**	**<0.001**	NS	NS	**<0.001**	NS
		Pan		**0.001**	**0.007**		**0.003**	**0.006**		**<0.001**	**0.032**
		Gorilla			**<0.001**			**<0.001**			**<0.001**
	Z_{pol}	*H.sapiens*	NS	**<0.001**	NS	NS	**<0.001**	NS	NS	**<0.001**	NS
		Pan		**<0.001**	NS		**<0.001**	NS		**<0.001**	NS
		Gorilla			**<0.001**			**<0.001**			**<0.001**
	J	*H.sapiens*	NS	**<0.001**	NS	NS	**<0.001**	NS	NS	**<0.001**	NS
		Pan		**<0.001**	NS		**<0.001**	NS		**<0.001**	NS
		Gorilla			**<0.001**			**<0.001**			**<0.001**
PP4	CA	*H.sapiens*	**0.003**	**<0.001**	NS	NS	**<0.001**	NS	NS	**<0.001**	NS
		Pan		**0.004**	**0.048**		**<0.001**	NS		**<0.001**	NS
		Gorilla			**<0.001**			**<0.001**			**<0.001**
	Z_{pol}	*H.sapiens*	NS	**<0.001**	NS	NS	**<0.001**	NS	NS	**<0.001**	NS
		Pan		**<0.001**	NS		**<0.001**	NS		**<0.001**	NS
		Gorilla			**<0.001**			**<0.001**			**<0.001**
	J	*H.sapiens*	NS	**<0.001**	NS	NS	**<0.001**	NS	NS	**<0.001**	NS
		Pan		**<0.001**	NS		**<0.001**	NS		**<0.001**	NS
		Gorilla			**<0.001**			**<0.001**			**<0.001**
PP5	CA	*H.sapiens*	NS	**<0.001**	NS	NS	**<0.001**	NS	NS	**<0.001**	NS
		Pan		**<0.001**	NS		**<0.001**	NS		**<0.001**	NS
		Gorilla			**<0.001**			**<0.001**			**<0.001**
	Z_{pol}	*H.sapiens*	NS	**<0.001**	NS	NS	**<0.001**	NS	NS	**<0.001**	NS
		Pan		**<0.001**	NS		**<0.001**	NS		**<0.001**	NS
		Gorilla			**<0.001**			**<0.001**			**<0.001**
	J	*H.sapiens*	NS	**<0.001**	NS	NS	**<0.001**	NS	NS	**<0.001**	NS
		Pan		**<0.001**	NS		**<0.001**	NS		**<0.001**	NS
		Gorilla			**<0.001**			**<0.001**			**<0.001**

Abbreviations: CA = cortical area; Z_{pol} = polar section modulus; J = polar second moment of area; NS = not significant (p>0.05).

Supplementary Table 4: Significance values for post hoc comparisons of cross-sectional properties within species, across the digits at 35%, 50% and 65% of the phalanx.

			35%			50%			65%		
			PP3	PP4	PP5	PP3	PP4	PP5	PP3	PP4	PP5
H. sapiens	CA	PP2	NS	NS	**0.017**	NS	NS	NS	NS	NS	NS
		PP3		NS	**0.008**		NS	NS		NS	**0.012**
		PP4			NS			NS			**0.076**
	Z_{pol}	PP2	NS	NS	**0.007**	NS	NS	**0.014**	NS	NS	**0.003**
		PP3		NS	**<0.001**		NS	**<0.001**		NS	**<0.001**
		PP4			NS			**0.027**			**<0.001**
	J	PP2	NS	NS	**0.006**	NS	NS	**0.011**	NS	NS	**0.004**
		PP3		NS	**<0.001**		NS	**<0.001**		NS	**<0.001**
		PP4			NS			**0.006**			**0.002**
Pan	CA	PP2	**0.047**	NS	NS	**0.006**	NS	**0.048**	**0.005**	NS	**0.049**
		PP3		NS	**<0.001**		NS	**<0.001**		NS	**<0.001**
		PP4			**0.010**			**<0.001**			**<0.001**
	Z_{pol}	PP2	**<0.001**	NS	NS	**<0.001**	NS	NS	**<0.001**	NS	**0.049**
		PP3		**0.004**	**<0.001**		NS	**<0.001**		NS	**<0.001**
		PP4			**<0.001**			**<0.001**			**<0.001**
	J	PP2	**<0.001**	NS	NS	**<0.001**	NS	NS	**<0.001**	NS	**0.011**
		PP3		NS	**<0.001**		NS	**<0.001**		NS	**<0.001**
		PP4			**<0.001**			**<0.001**			**<0.001**
Gorilla	CA	PP2	NS	NS	NS	NS	NS	NS	NS	NS	NS
		PP3		NS	**0.005**		NS	**0.027**		NS	**0.012**
		PP4			NS			NS			NS
	Z_{pol}	PP2	NS	NS	NS	NS	NS	NS	NS	NS	NS
		PP3		NS	**<0.001**		NS	**<0.001**		NS	**<0.001**
		PP4			NS			NS			NS
	J	PP2	NS	NS	NS	NS	NS	NS	NS	NS	NS
		PP3		NS	**<0.001**		NS	**<0.001**		NS	**<0.001**
		PP4			NS			NS			NS

Abbreviations: CA = cortical area; Z_{pol} = polar section modulus; J = polar second moment of area; NS = not significant (p>0.05).

Note: The proximal phalanges *Pongo* are not represented because they did not show significant differences.

Supplementary Table 5: Correlation statistics testing the relationship between phalangeal curvature and cortical thickness of the phalangeal shaft for the total sample and within species. All four digits were pooled.

	R^2	p
Total sample	0.030	**0.001**
H. sapiens	0.028	NS
Pan	0.106	**0.001**
Gorilla	0.005	NS
Pongo	0.003	NS

Abbreviations: NS = not significant (p>0.05).

Supplementary Figure 1: Cortical thickness distribution maps of each individual used in the study. Specimen IDs of individuals are under each map.

Supplementary Figure 2: 3D PCAs for cortical bone distribution of proximal phalanges of PP2, PP3, PP4, and PP5 of *H.sapiens*, *Pan* sp., *Gorilla,* and *Pongo* sp.

Supplementary Figure 3: Boxplots representing the mean cortical thickness across the shaft for (A) PP2, (B) PP3, (C) PP4, and (D) PP5 of *H. sapiens*, *Pan* sp., *Gorilla*, and *Pongo* sp. *** = $p<0.001$. The African apes are significantly thicker than *H. sapiens* and *Pongo*.

Supplementary Figure 4: Boxplots representing the mean cortical thickness across the shaft for (A) *H. sapiens*, (B) *Pan*, (C) *Gorilla*, and (D) *Pongo* of digits 2-5. * = $p<0.05$. *Pan* PP3 is significantly thicker than *Pan* PP5.

Supplementary Figure 5: Average CA plotted from the proximal end to the distal end of the phalangeal shaft of *H. sapiens*, *Pan*, *Gorilla*, and *Pongo.* (A) PP2; (B) PP3; (C) PP4; (D) PP5.

Supplementary Figure 6: Average Z_{pol} plotted from the proximal end to the distal end of the phalangeal shaft of *H. sapiens, Pan*, *Gorilla*, and *Pongo.* (A) PP2; (B) PP3; (C) PP4; (D) PP5.

Supplementary Figure 7: Average J plotted from the proximal end to the distal end of the phalangeal shaft of *H. sapiens*, *Pan*, *Gorilla*, and *Pongo.* (A) PP2; (B) PP3; (C) PP4; (D) PP5.

Supplementary Figure 8: Scatterplot of phalangeal curvature against phalangeal shaft cortical thickness of digits 2-5 in *H. sapiens*, *Pan* sp., *Gorilla*, and *Pongo* sp.

Pongo PP2

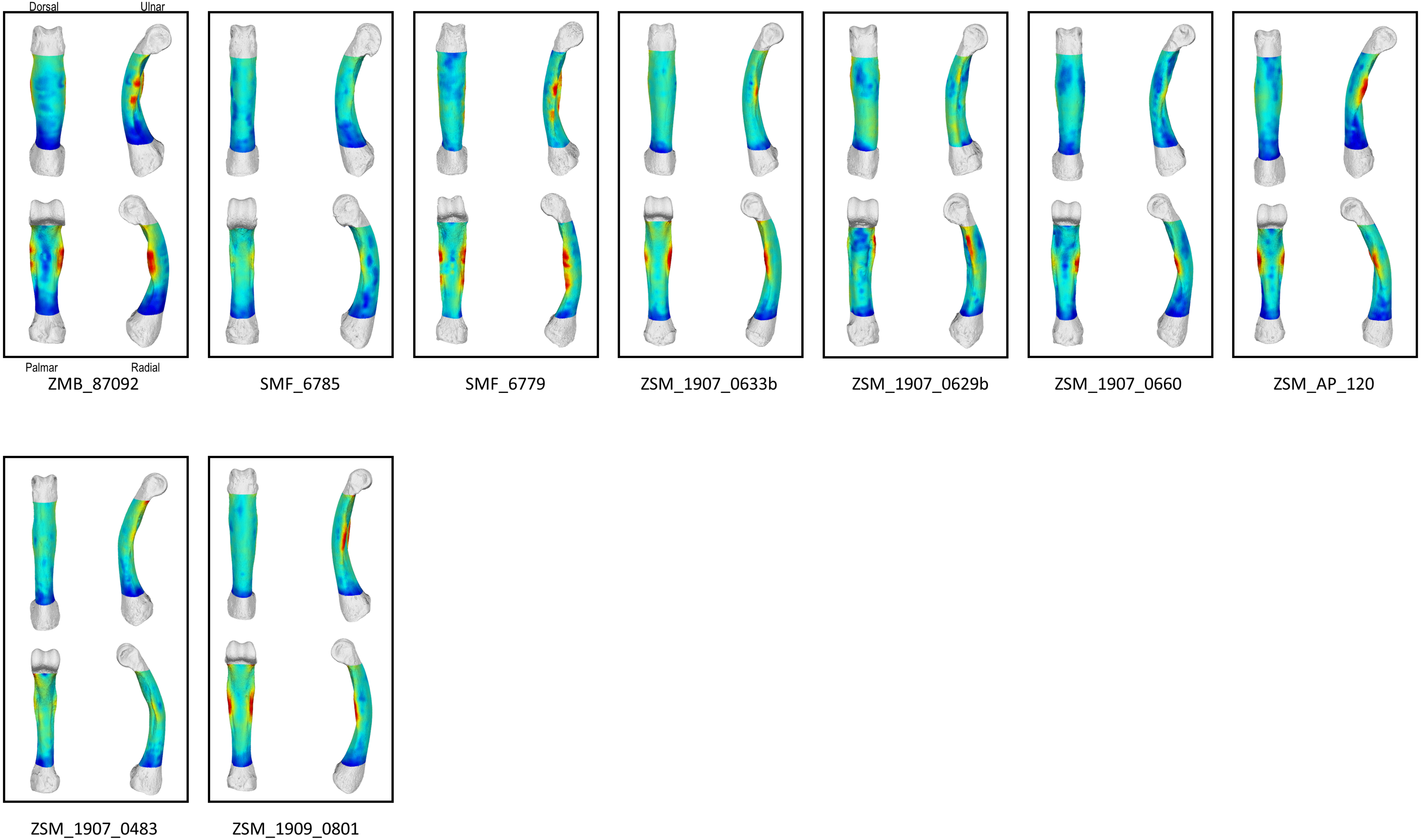
Dorsal
Ulnar
Palmar
Radial
ZMB_87092
SMF_6785
SMF_6779
ZSM_1907_0633b
ZSM_1907_0629b
ZSM_1907_0660
ZSM_AP_120
ZSM_1907_0483
ZSM_1909_0801

Pongo PP3

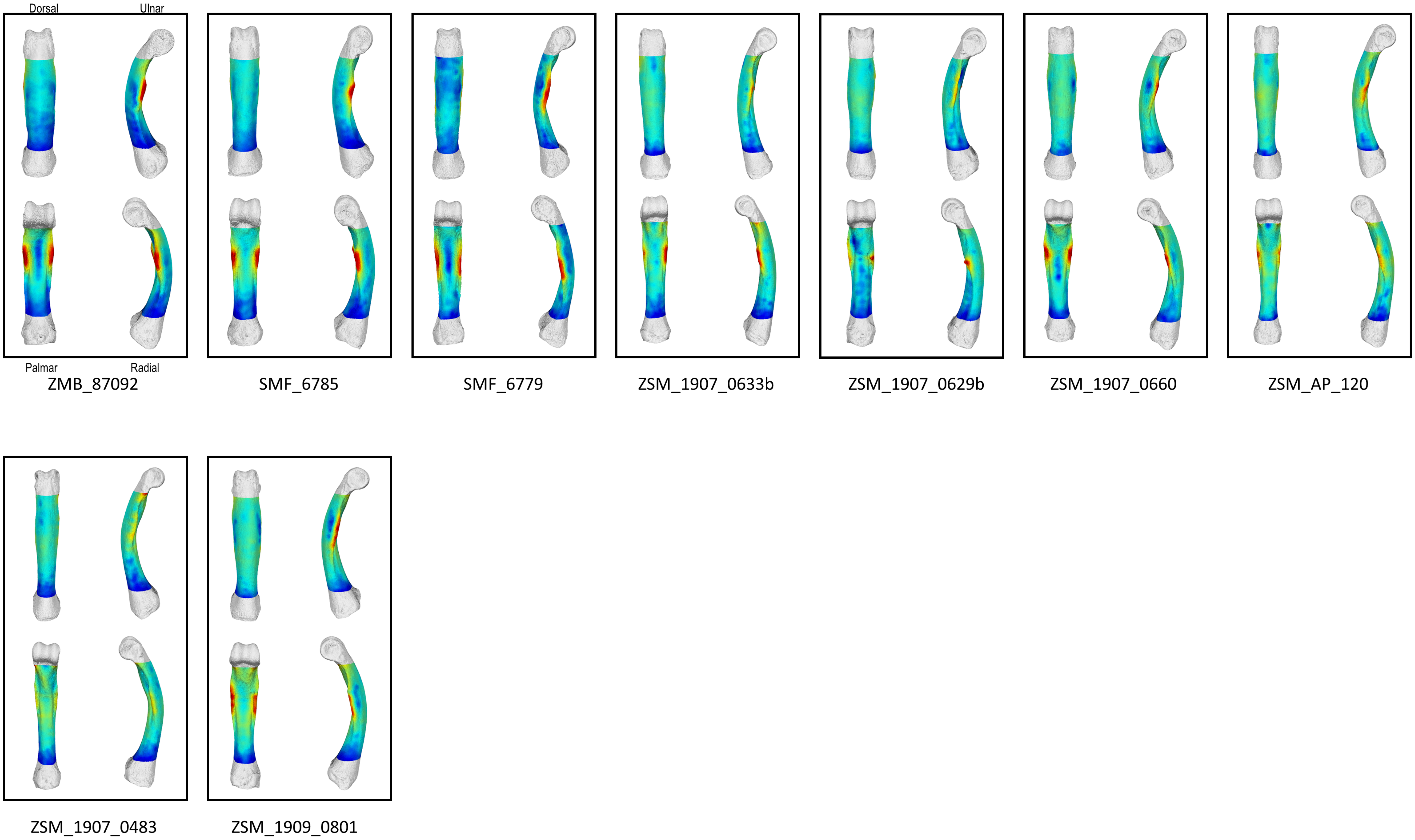
Dorsal
Ulnar
Palmar
Radial
ZMB_87092
SMF_6785
SMF_6779
ZSM_1907_0633b
ZSM_1907_0629b
ZSM_1907_0660
ZSM_AP_120
ZSM_1907_0483
ZSM_1909_0801

Pongo PP4

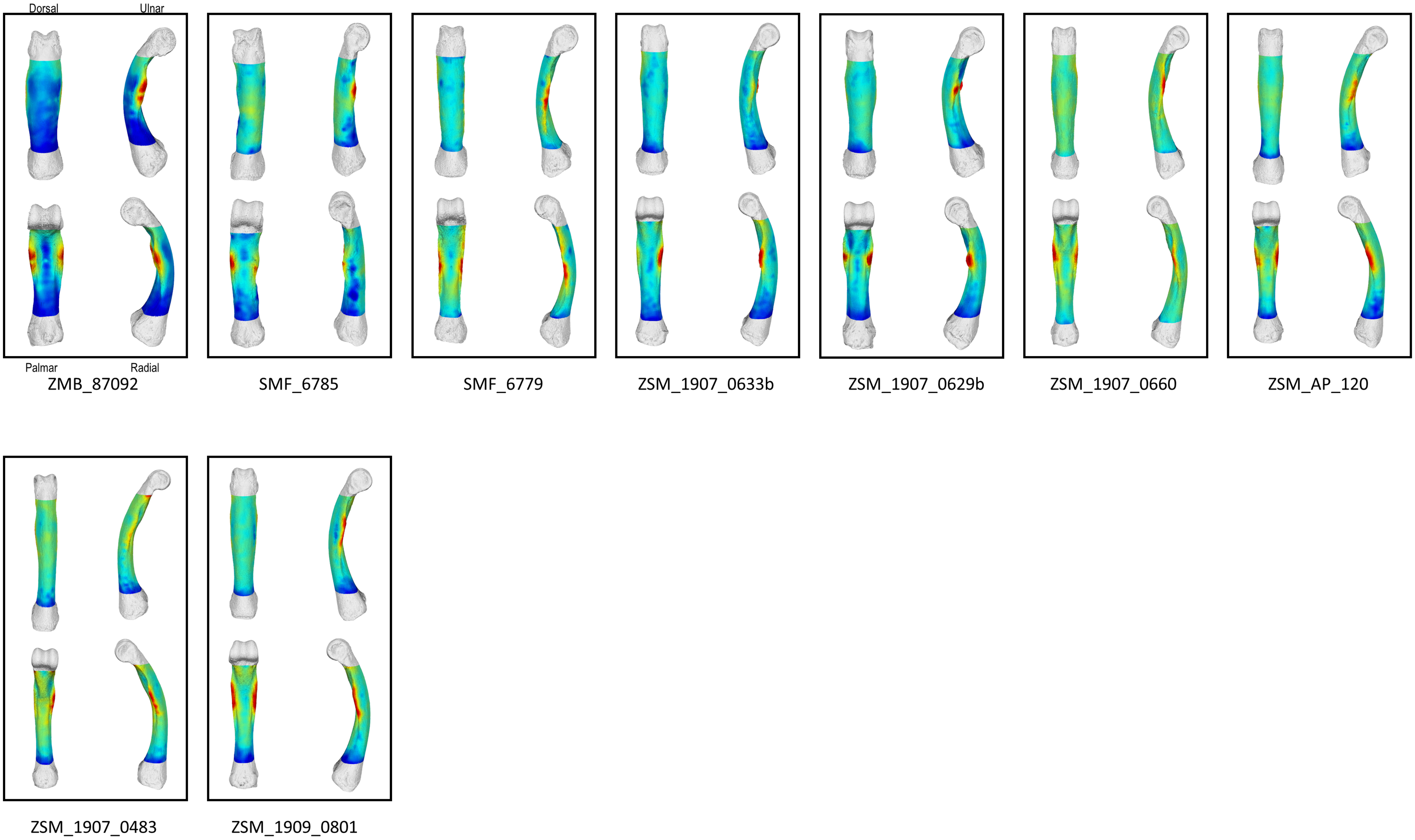
Dorsal
Ulnar
Palmar
Radial
ZMB_87092
SMF_6785
SMF_6779
ZSM_1907_0633b
ZSM_1907_0629b
ZSM_1907_0660
ZSM_AP_120
ZSM_1907_0483
ZSM_1909_0801

Pongo PP5

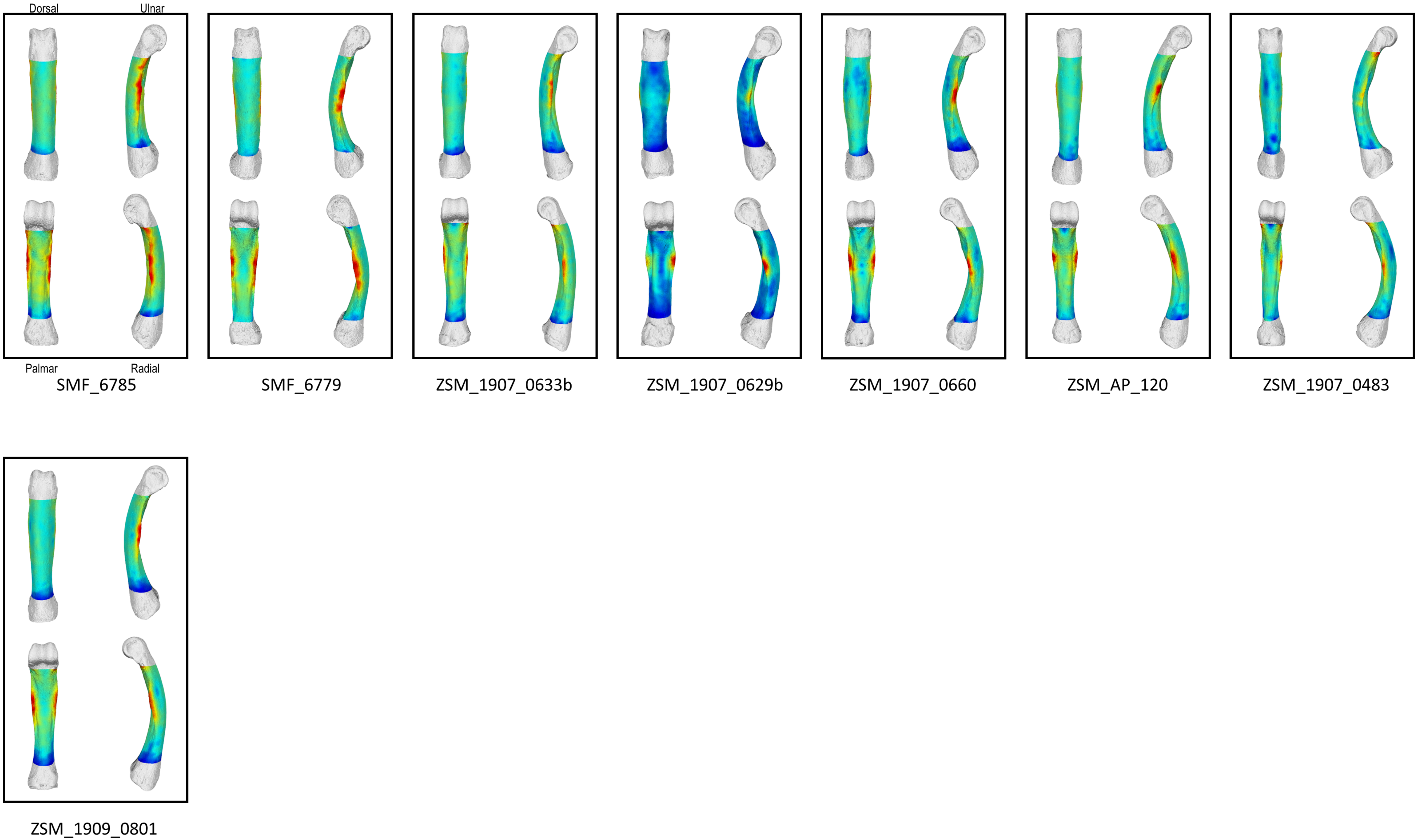
Dorsal
Ulnar
Palmar
Radial
SMF_6785
SMF_6779
ZSM_1907_0633b
ZSM_1907_0629b
ZSM_1907_0660
ZSM_AP_120
ZSM_1907_0483
ZSM_1909_0801

Gorilla PP2

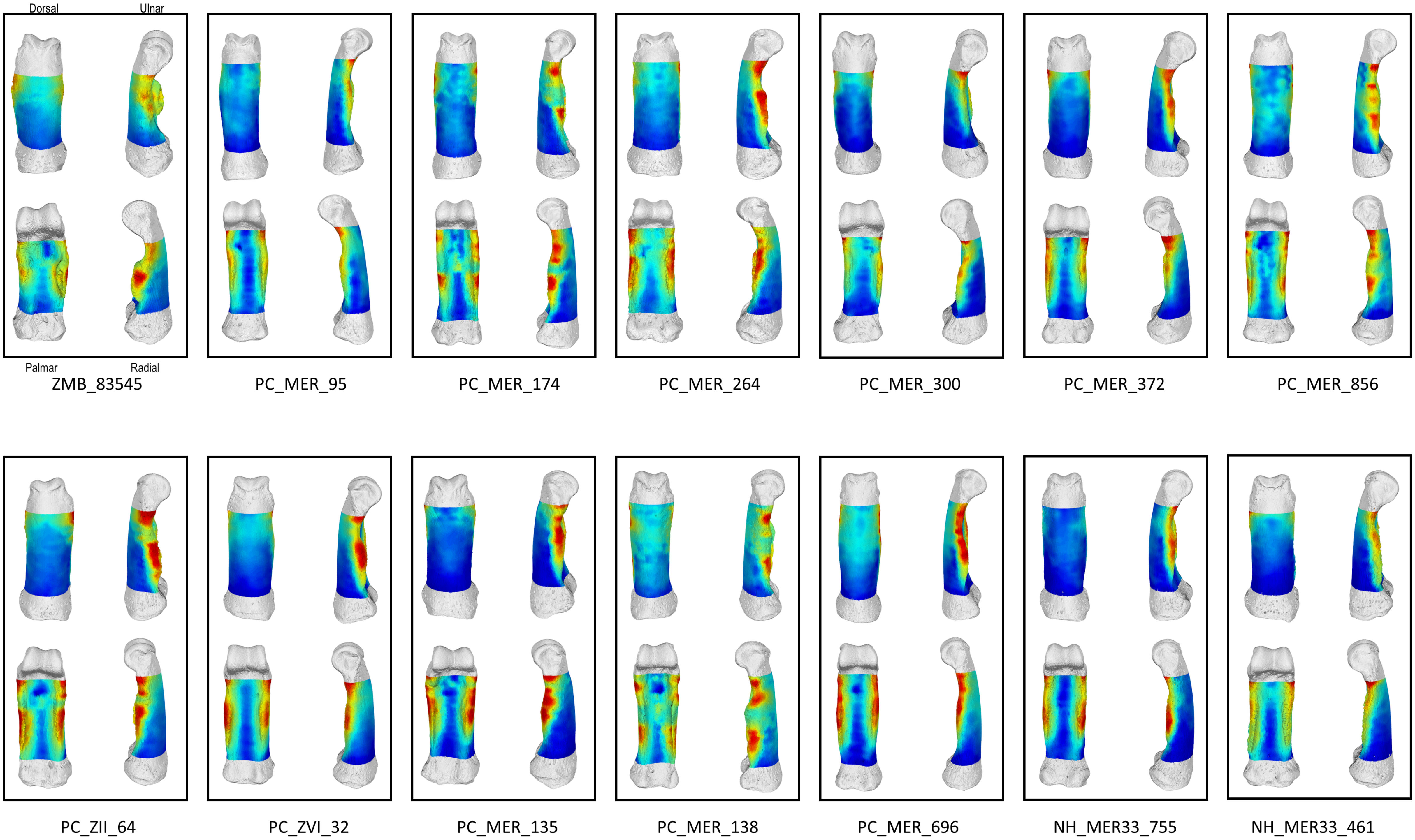

Dorsal
Ulnar
Palmar
Radial
ZMB_83545
PC_MER_95
PC_MER_174
PC_MER_264
PC_MER_300
PC_MER_372
PC_MER_856
PC_ZII_64
PC_ZVI_32
PC_MER_135
PC_MER_138
PC_MER_696
NH_MER33_755
NH_MER33_461

Gorilla PP2

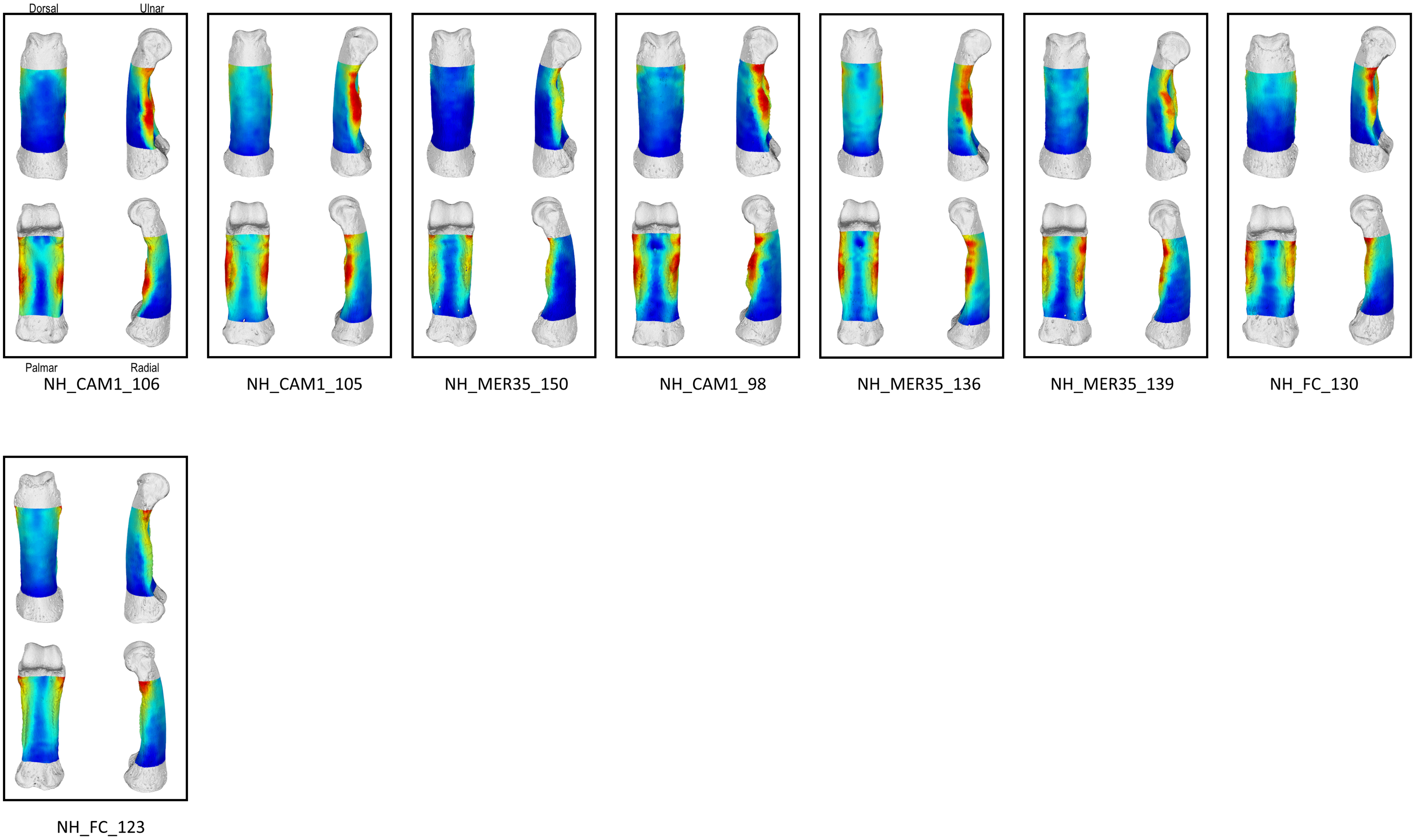
Dorsal
Ulnar
Palmar
Radial
NH_CAM1_106
NH_CAM1_105
NH_MER35_150
NH_CAM1_98
NH_MER35_136
NH_MER35_139
NH_FC_130
NH_FC_123

Gorilla PP3

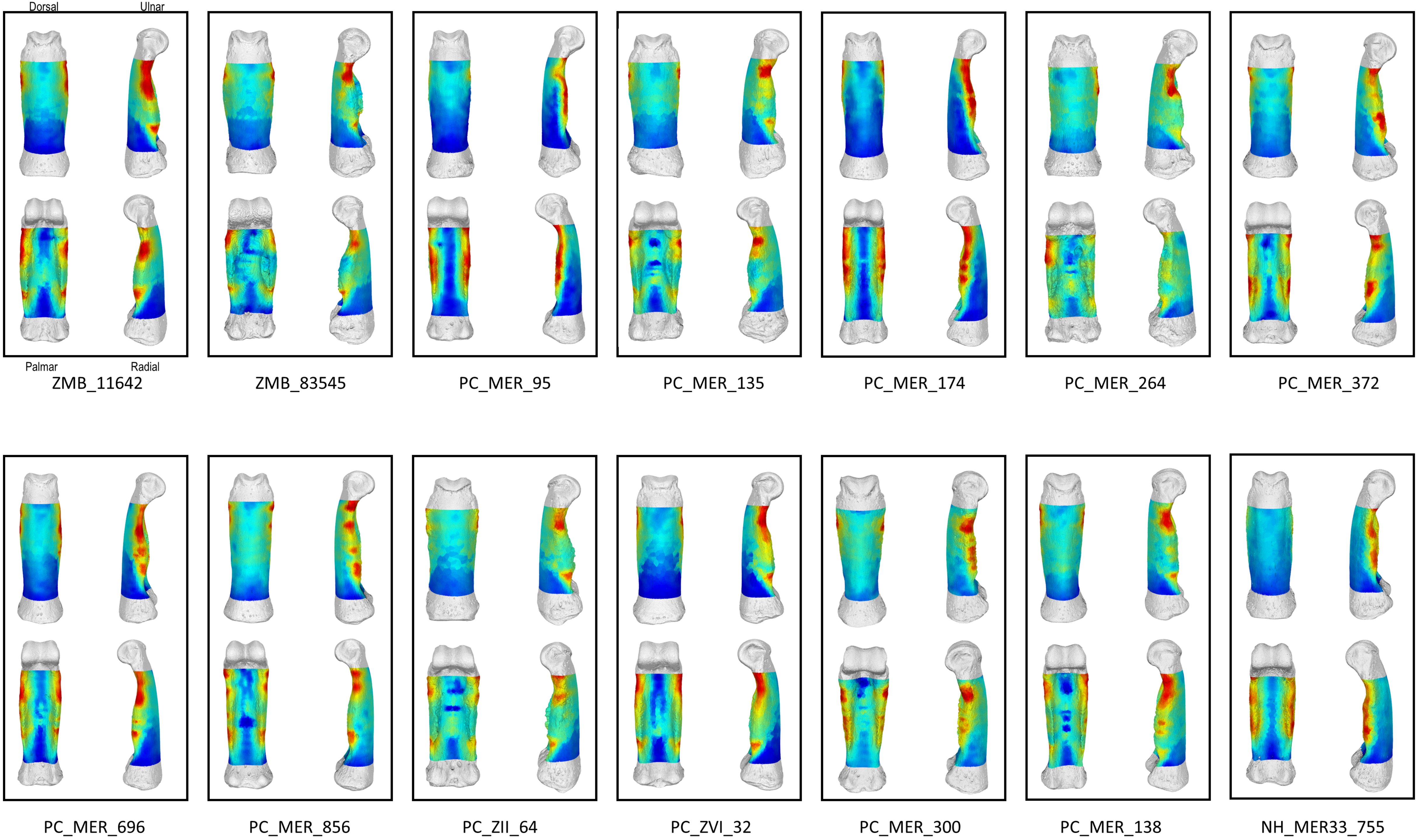
Dorsal
Ulnar
Palmar
Radial
ZMB_11642
ZMB_83545
PC_MER_95
PC_MER_135
PC_MER_174
PC_MER_264
PC_MER_372
PC_MER_696
PC_MER_856
PC_ZII_64
PC_ZVI_32
PC_MER_300
PC_MER_138
NH_MER33_755

Gorilla PP3

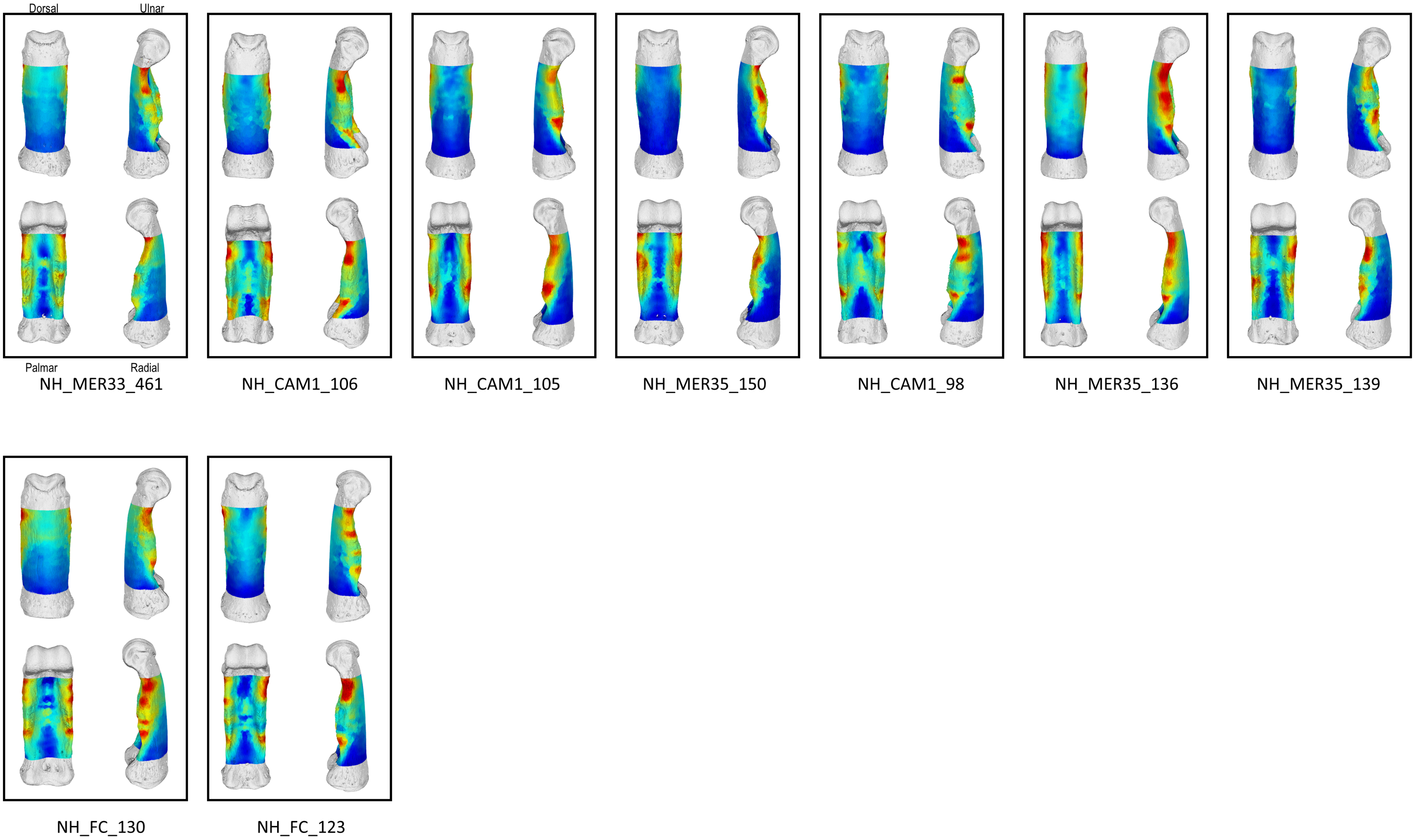

Dorsal
Ulnar
Palmar
Radial
NH_MER33_461
NH_CAM1_106
NH_CAM1_105
NH_MER35_150
NH_CAM1_98
NH_MER35_136
NH_MER35_139
NH_FC_130
NH_FC_123

Gorilla PP4

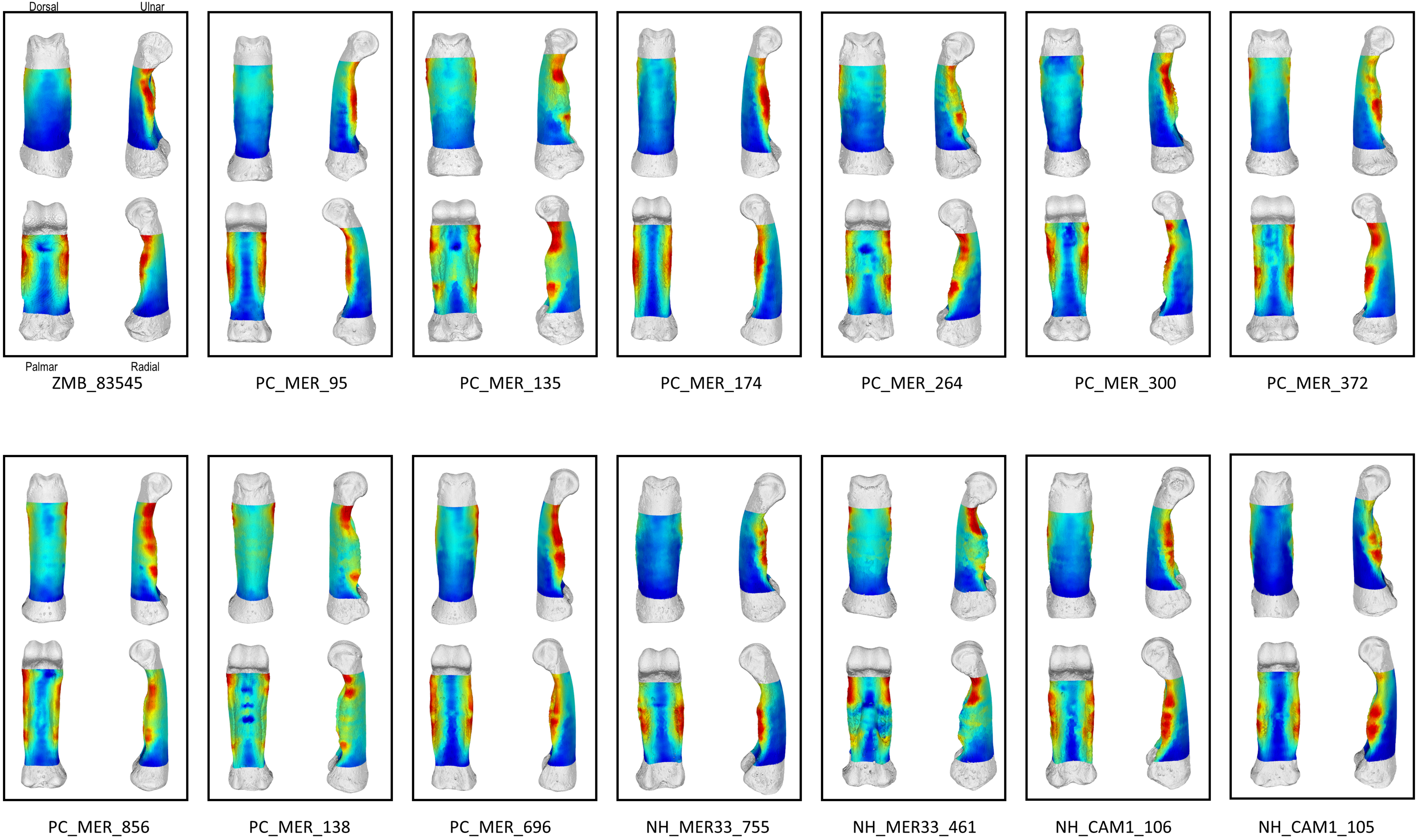
Dorsal
Ulnar
Palmar
Radial
ZMB_83545
PC_MER_95
PC_MER_135
PC_MER_174
PC_MER_264
PC_MER_300
PC_MER_372
PC_MER_856
PC_MER_138
PC_MER_696
NH_MER33_755
NH_MER33_461
NH_CAM1_106
NH_CAM1_105

Gorilla PP4

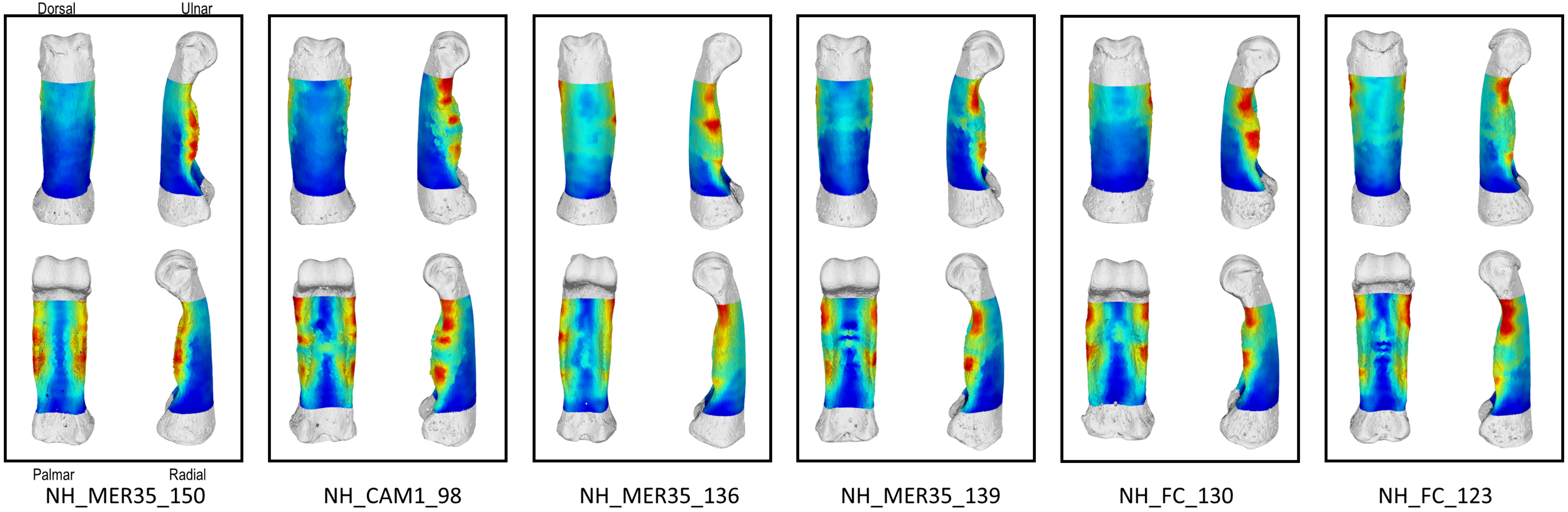

Dorsal
Ulnar
Palmar
Radial
NH_MER35_150
NH_CAM1_98
NH_MER35_136
NH_MER35_139
NH_FC_130
NH_FC_123

Gorilla PP5

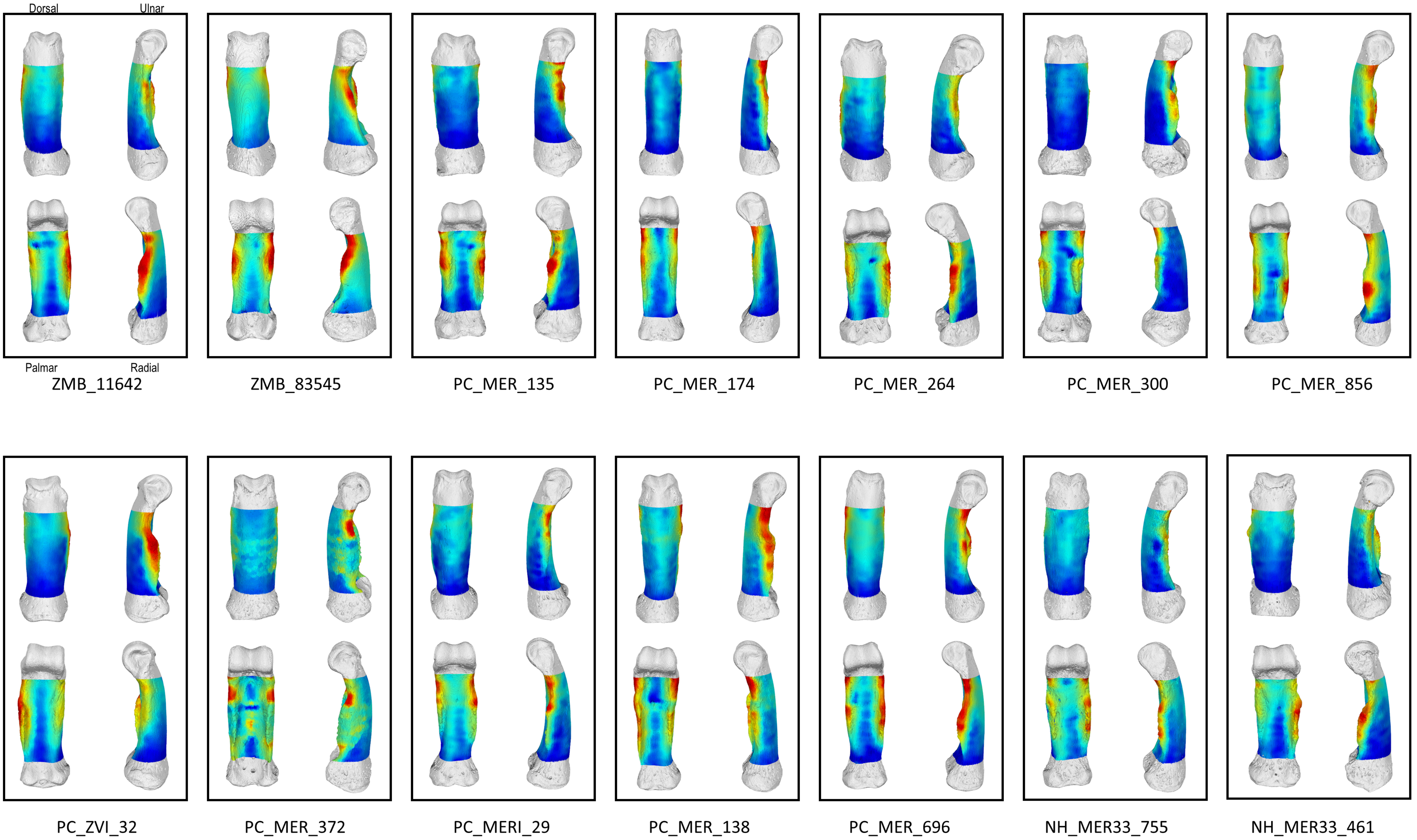
Dorsal
Ulnar
Palmar
Radial
ZMB_11642
ZMB_83545
PC_MER_135
PC_MER_174
PC_MER_264
PC_MER_300
PC_MER_856
PC_ZVI_32
PC_MER_372
PC_MERI_29
PC_MER_138
PC_MER_696
NH_MER33_755
NH_MER33_461

Gorilla PP5

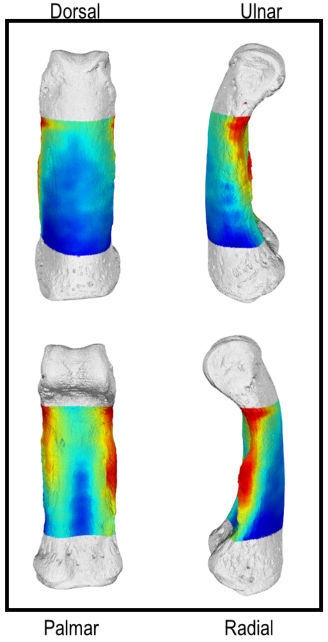

NH_CAM1_106

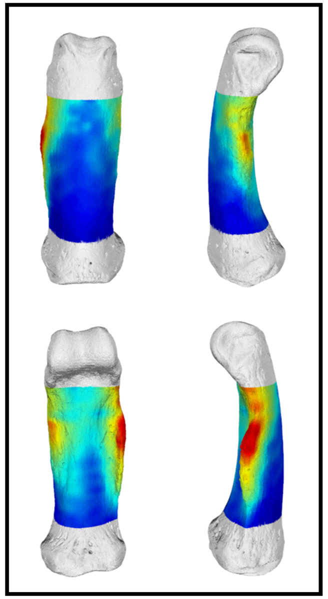

NH_CAM1_105

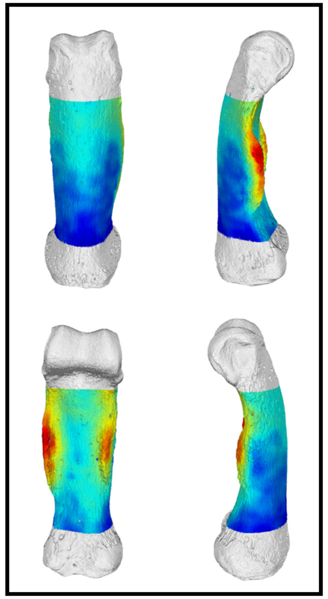

NH_MER35_150

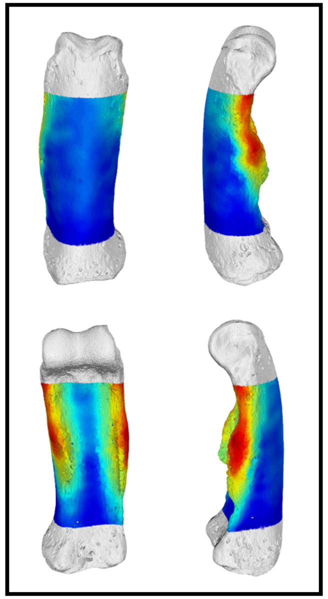

NH_CAM1_98

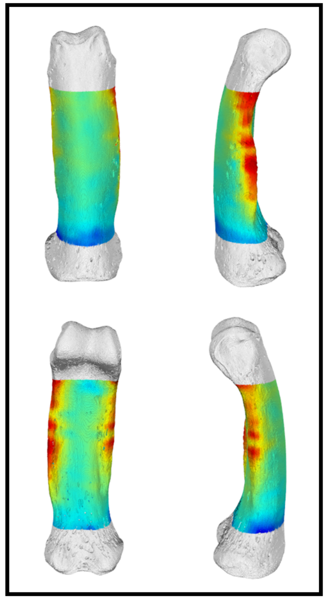

NH_MER35_136

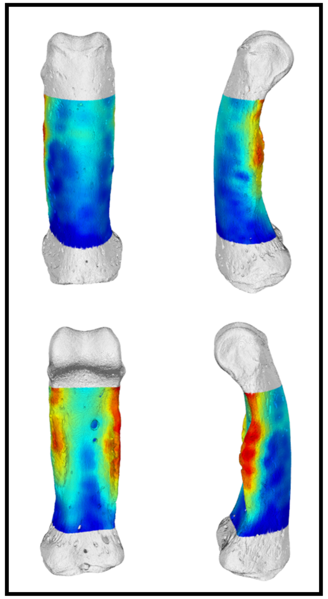

NH_MER35_139

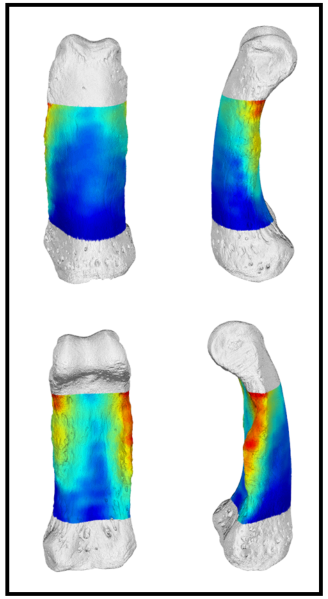

NH_FC_130

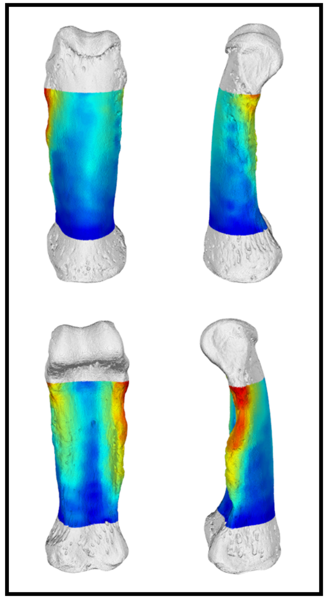

NH_FC_123

Pan PP2

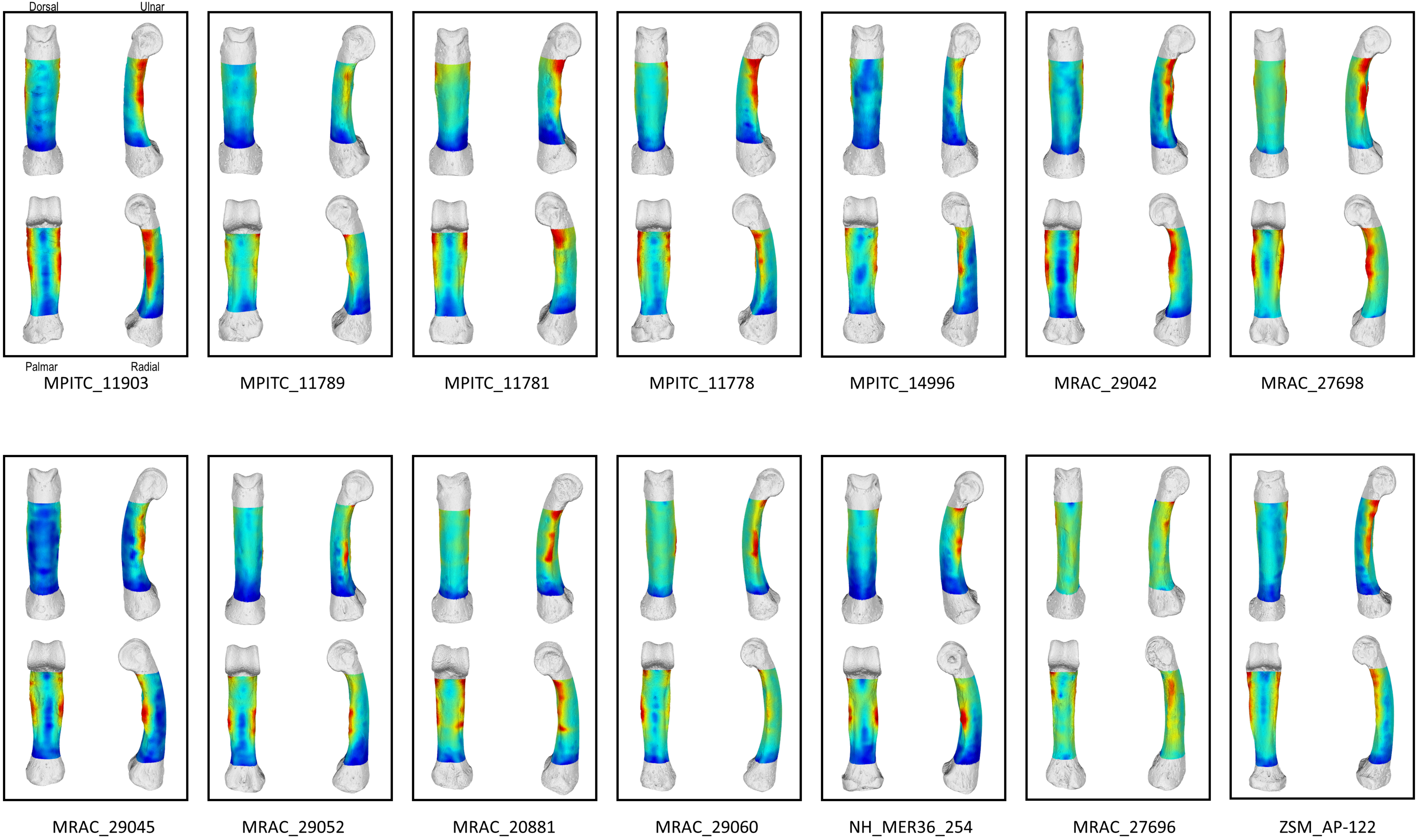
Dorsal
Ulnar
Palmar
Radial
MPITC_11903
MPITC_11789
MPITC_11781
MPITC_11778
MPITC_14996
MRAC_29042
MRAC_27698
MRAC_29045
MRAC_29052
MRAC_20881
MRAC_29060
NH_MER36_254
MRAC_27696
ZSM_AP-122

Pan PP2

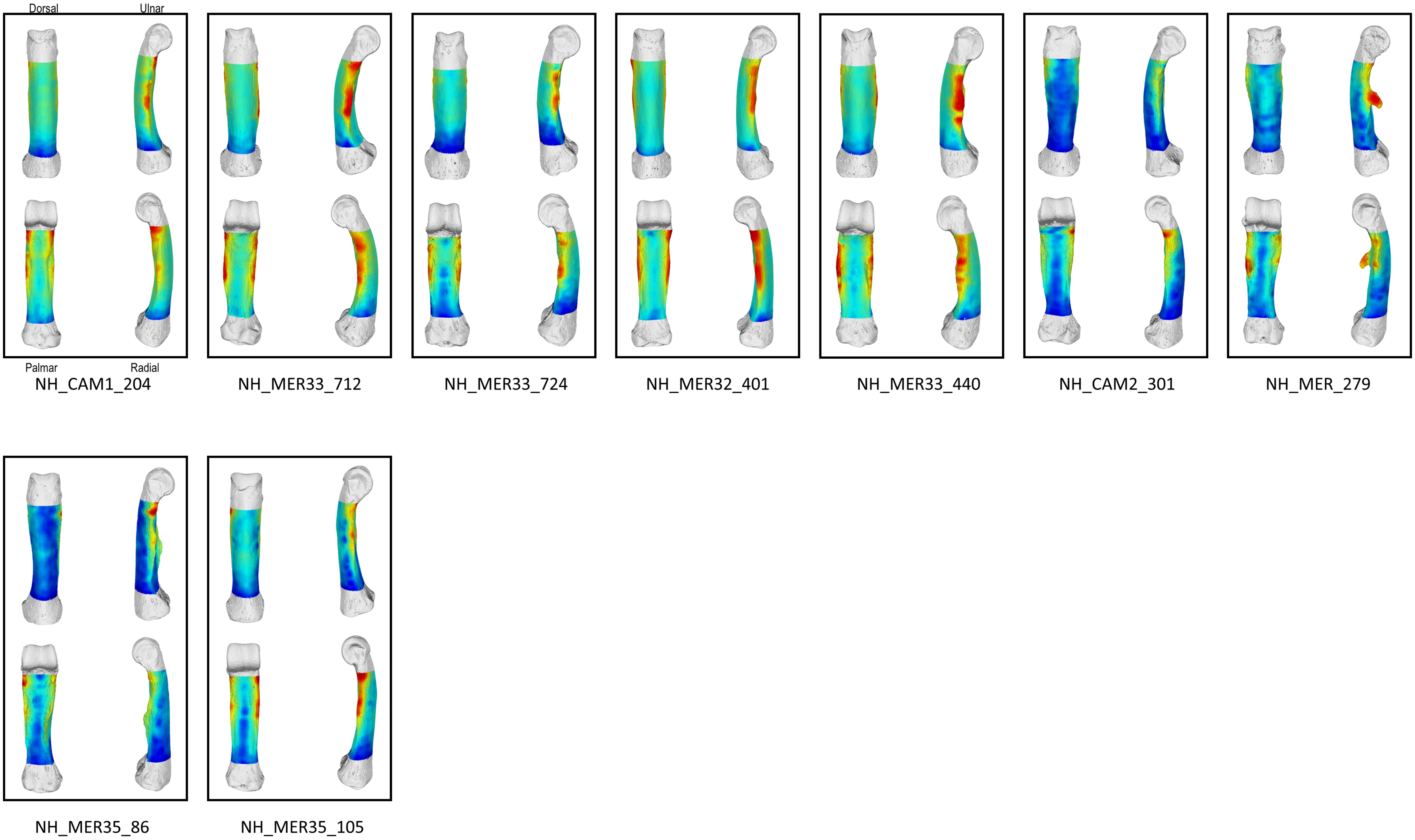

Dorsal
Ulnar
Palmar
Radial
NH_CAM1_204
NH_MER33_712
NH_MER33_724
NH_MER32_401
NH_MER33_440
NH_CAM2_301
NH_MER_279
NH_MER35_86
NH_MER35_105

Pan PP3

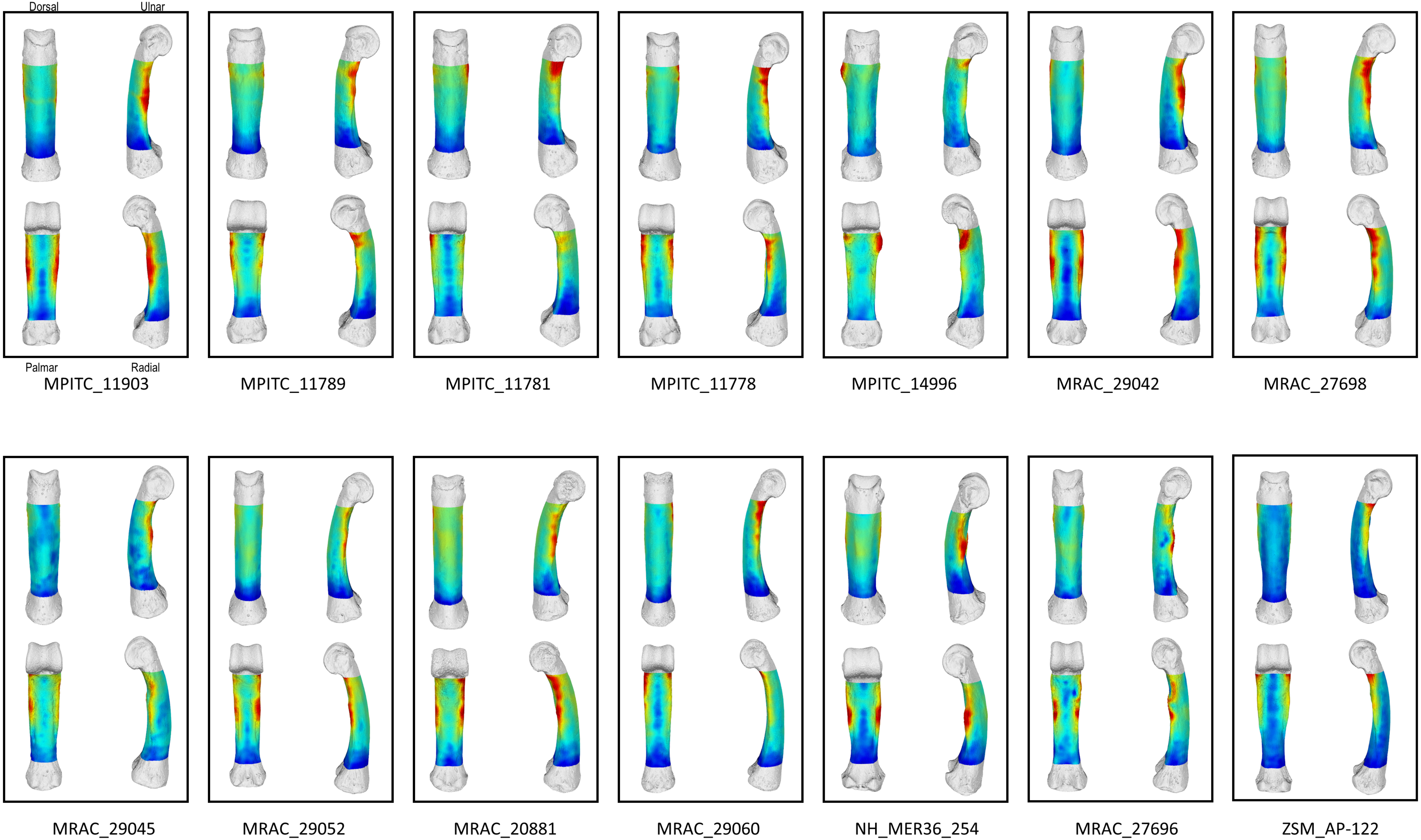
Dorsal
Ulnar
Palmar
Radial
MPITC_11903
MPITC_11789
MPITC_11781
MPITC_11778
MPITC_14996
MRAC_29042
MRAC_27698
MRAC_29045
MRAC_29052
MRAC_20881
MRAC_29060
NH_MER36_254
MRAC_27696
ZSM_AP-122

Pan PP3

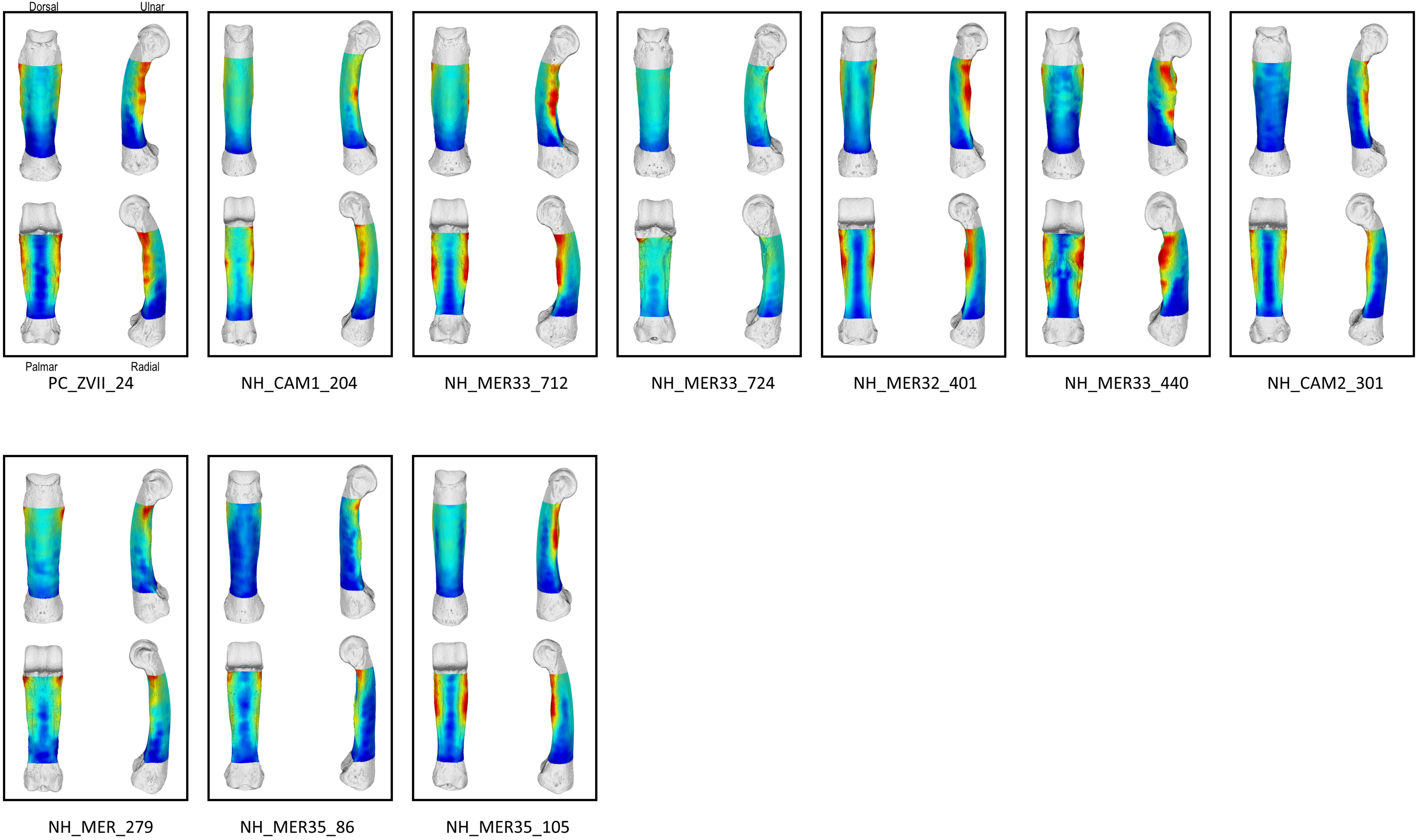
Dorsal
Ulnar
Palmar
Radial
PC_ZVII_24
NH_CAM1_204
NH_MER33_712
NH_MER33_724
NH_MER32_401
NH_MER33_440
NH_CAM2_301
NH_MER_279
NH_MER35_86
NH_MER35_105

Pan PP4

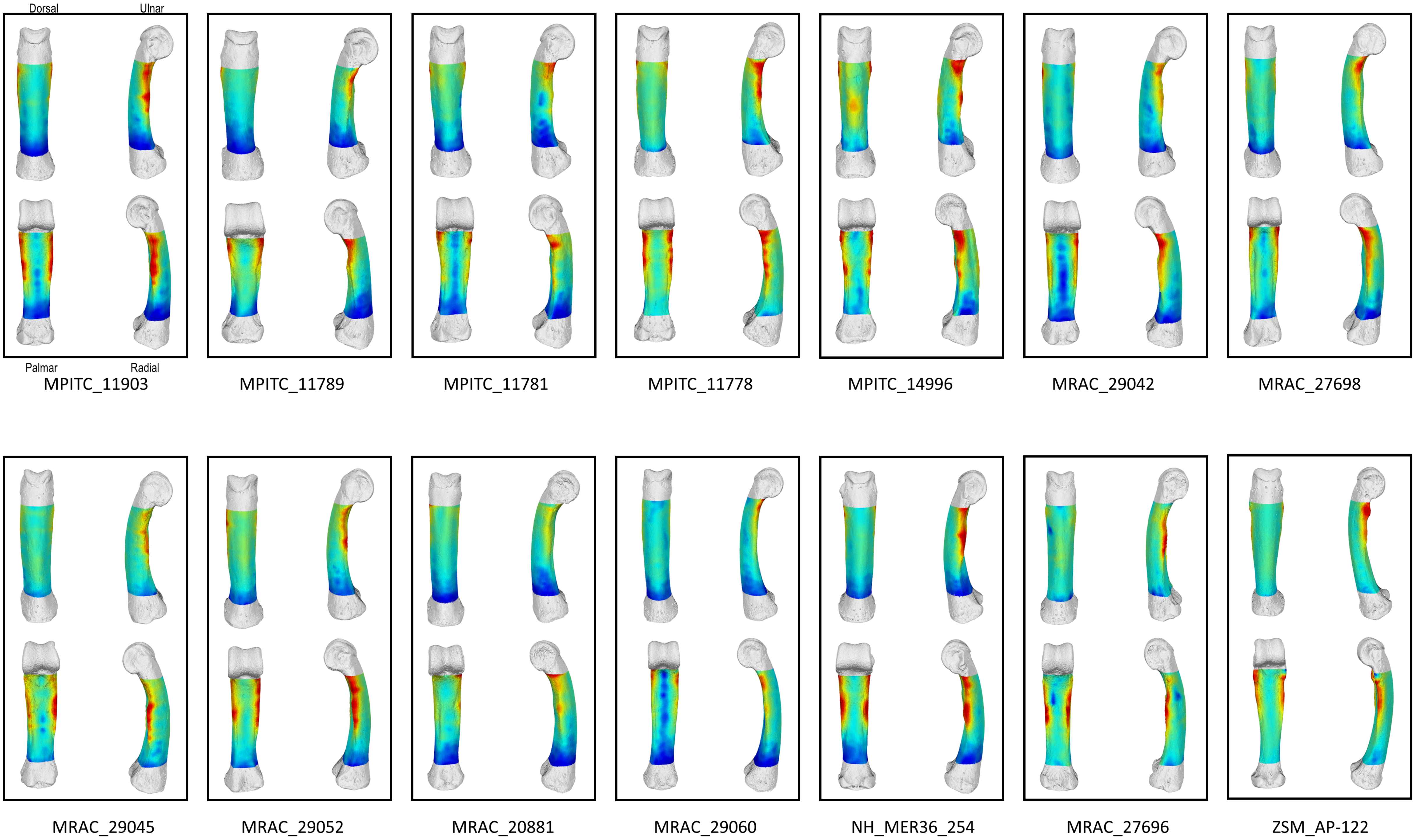
Dorsal
Ulnar
Palmar
Radial
MPITC_11903
MPITC_11789
MPITC_11781
MPITC_11778
MPITC_14996
MRAC_29042
MRAC_27698
MRAC_29045
MRAC_29052
MRAC_20881
MRAC_29060
NH_MER36_254
MRAC_27696
ZSM_AP-122

Pan PP4

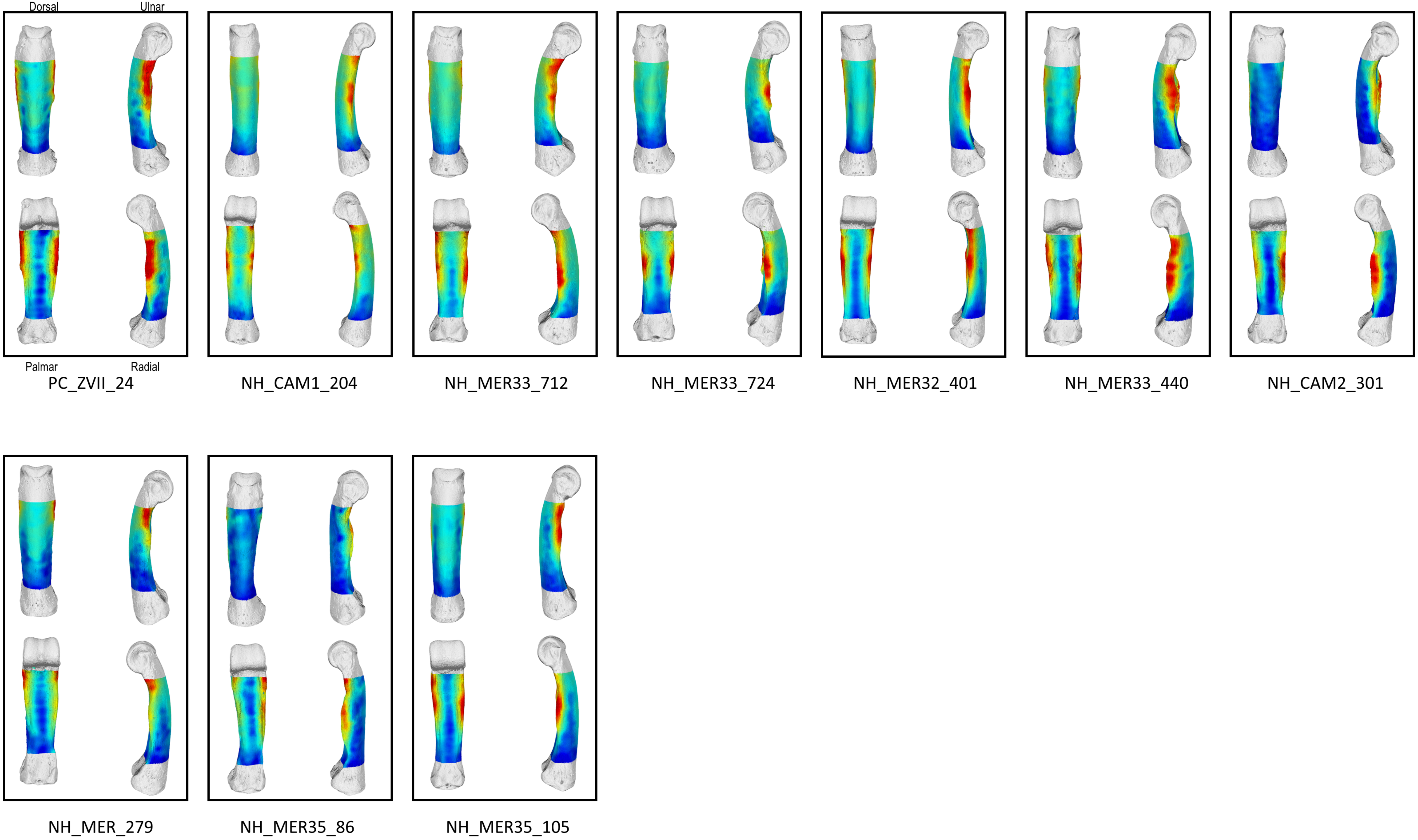
Dorsal
Ulnar
Palmar
Radial
PC_ZVII_24
NH_CAM1_204
NH_MER33_712
NH_MER33_724
NH_MER32_401
NH_MER33_440
NH_CAM2_301
NH_MER_279
NH_MER35_86
NH_MER35_105

Pan PP5

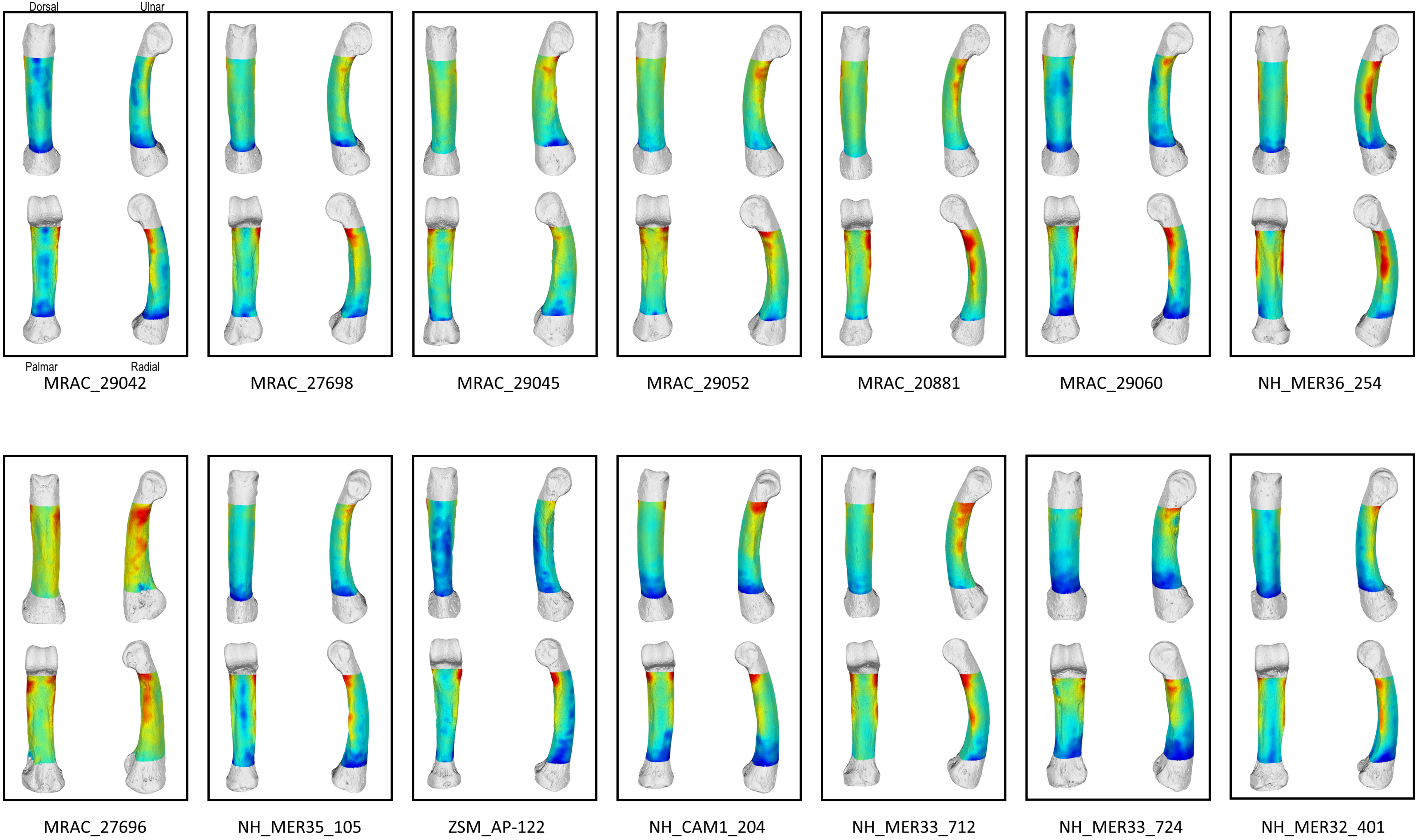

Dorsal
Ulnar
Palmar
Radial
MRAC_29042
MRAC_27698
MRAC_29045
MRAC_29052
MRAC_20881
MRAC_29060
NH_MER36_254
MRAC_27696
NH_MER35_105
ZSM_AP-122
NH_CAM1_204
NH_MER33_712
NH_MER33_724
NH_MER32_401

Pan PP5

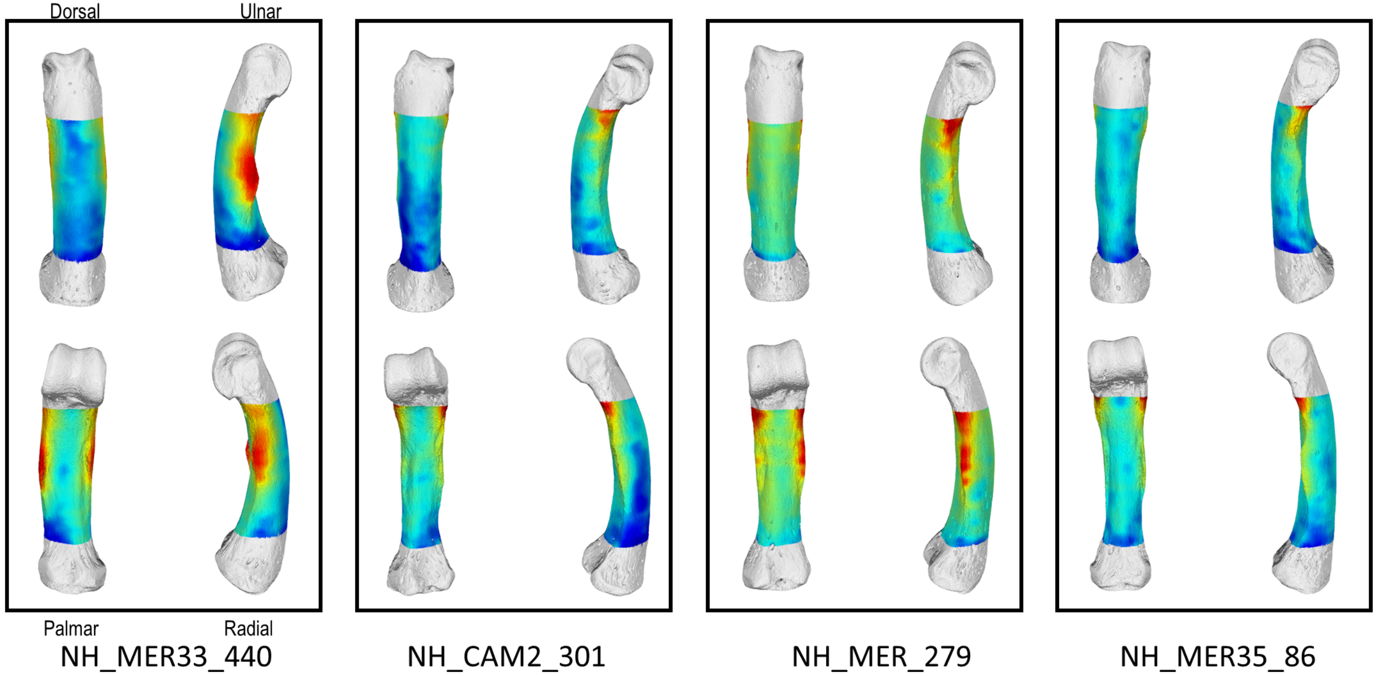
Dorsal
Ulnar
Palmar
Radial
NH_MER33_440
NH_CAM2_301
NH_MER_279
NH_MER35_86

H. sapiens PP2

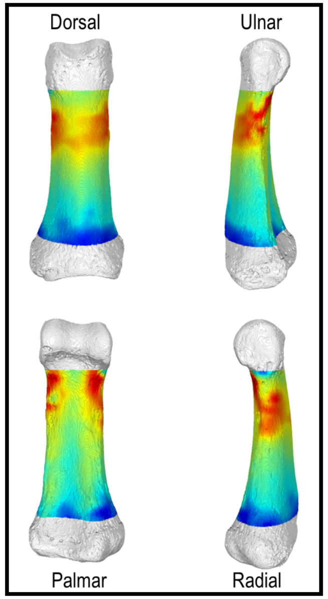

81_H1035

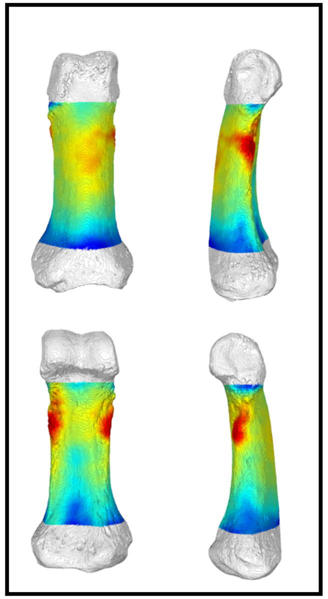

X36_FCS16

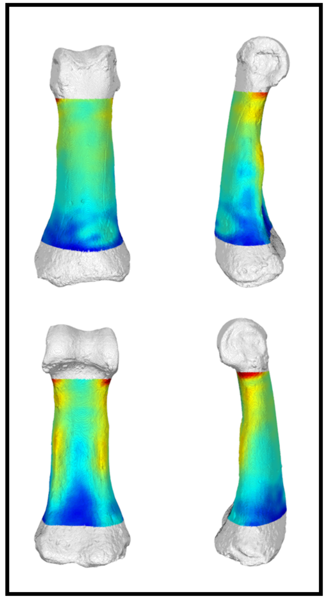

ARENE_CANDIDE_2

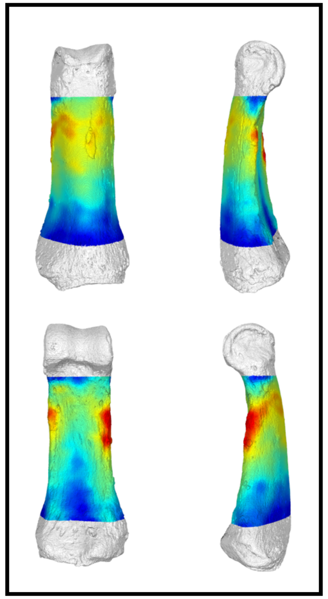

Barma_Grande_2

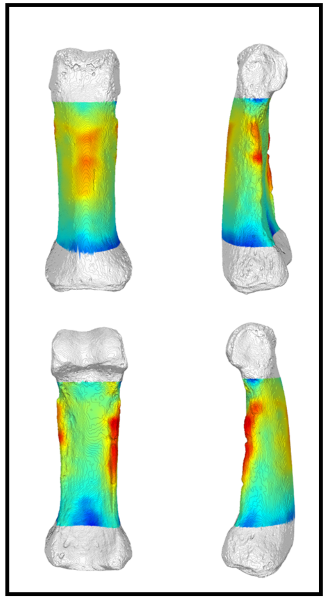

X37_FCS17

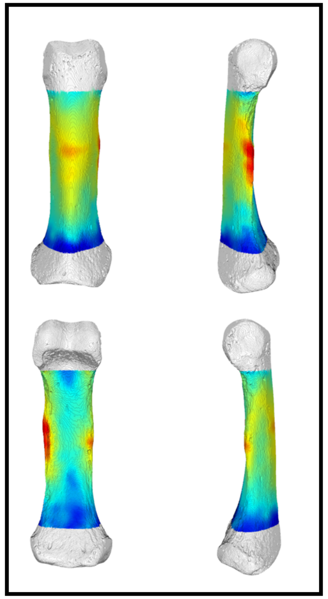

DCW_AM_3_0_2

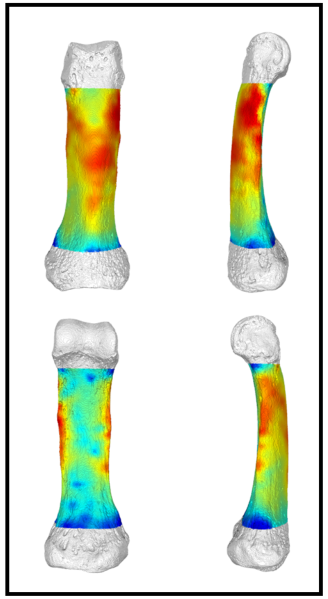

DCW_OC_1_0_141

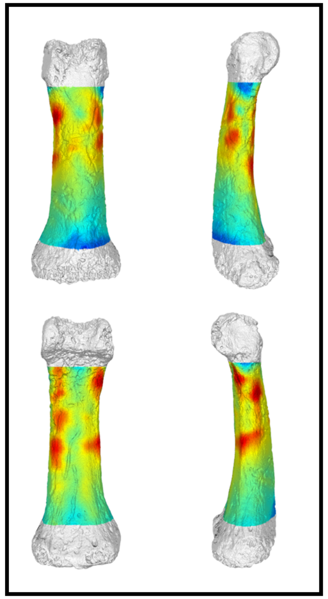

DV13_93

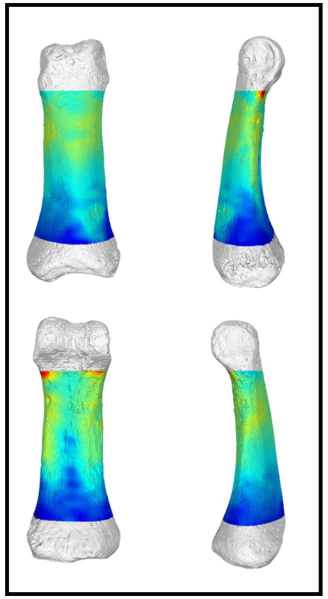

DV15_112

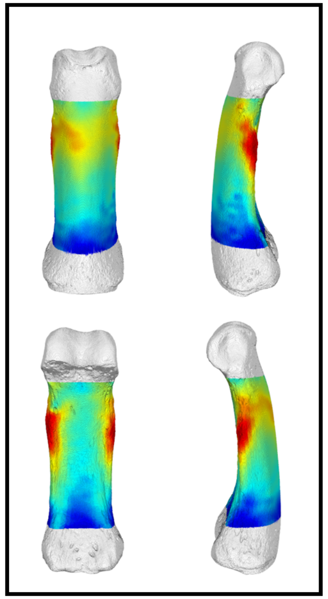

FCS8

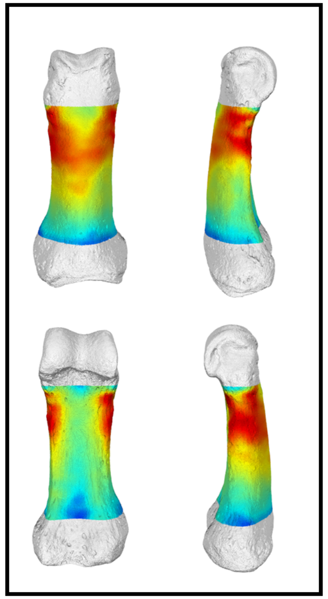

GAUG_Inden-113

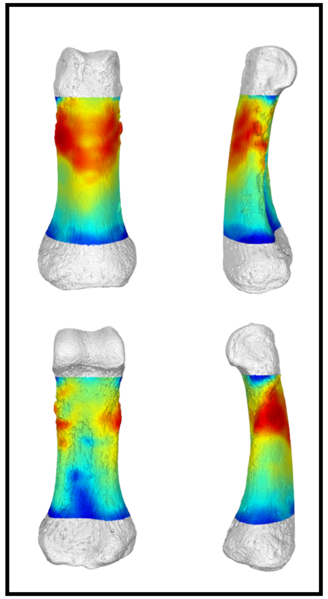

GAUG_Inden-119

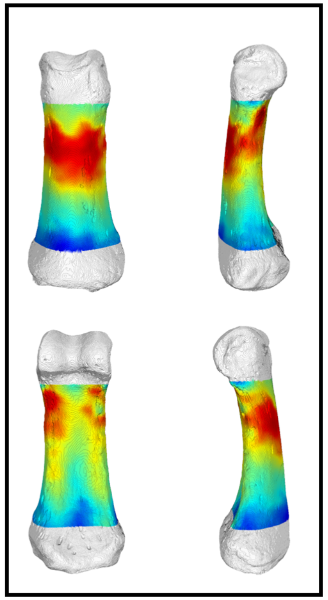

X38_FCS17

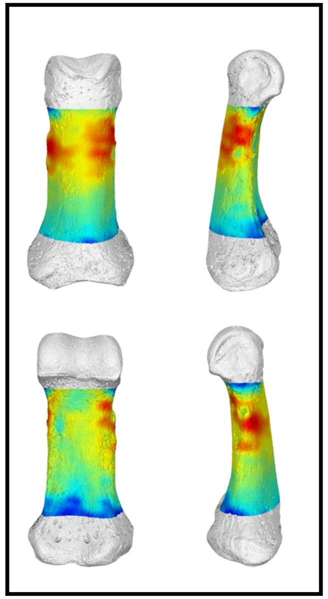

GAUG_Inden-243

H. sapiens PP2

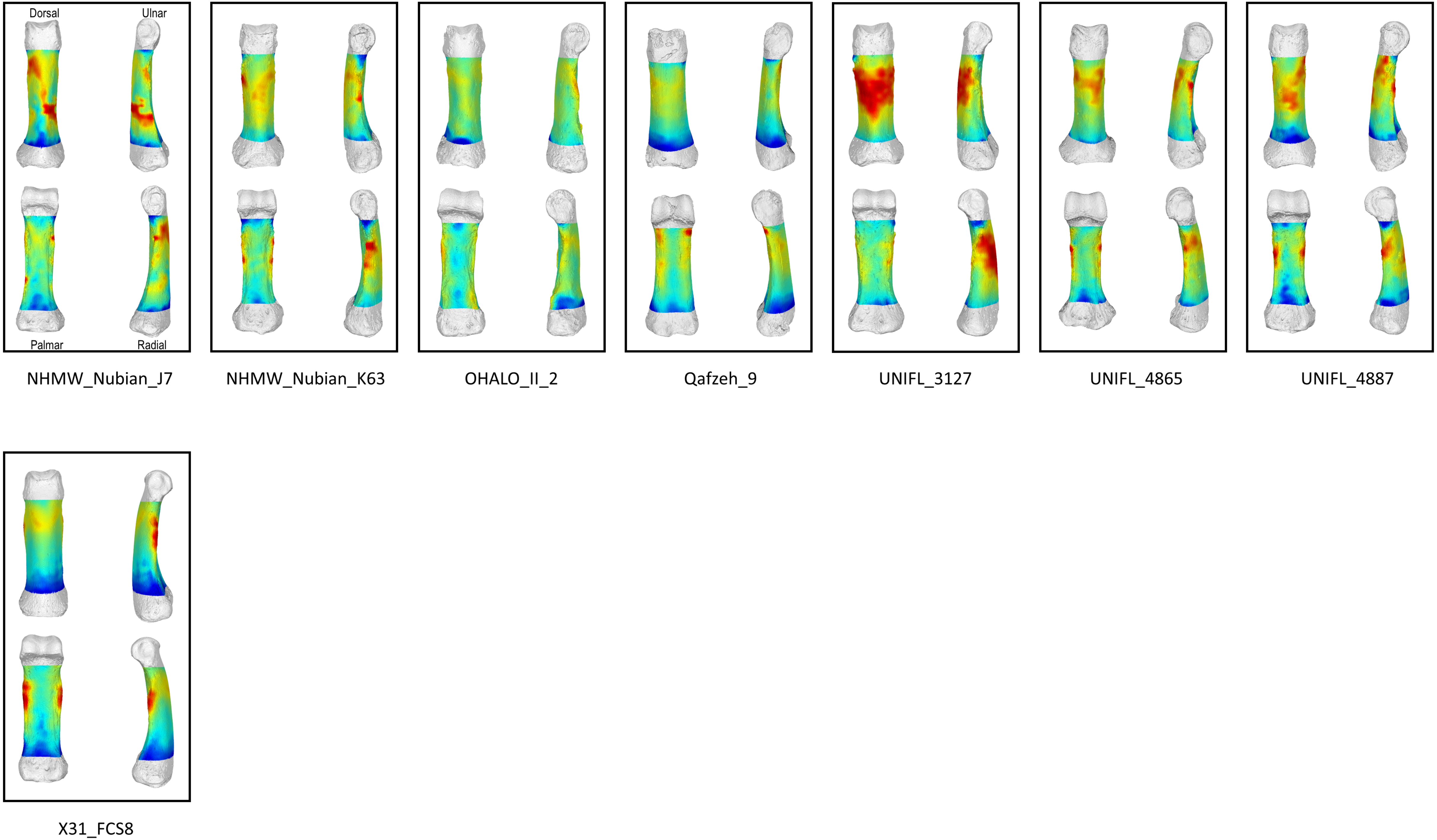

H. sapiens PP3

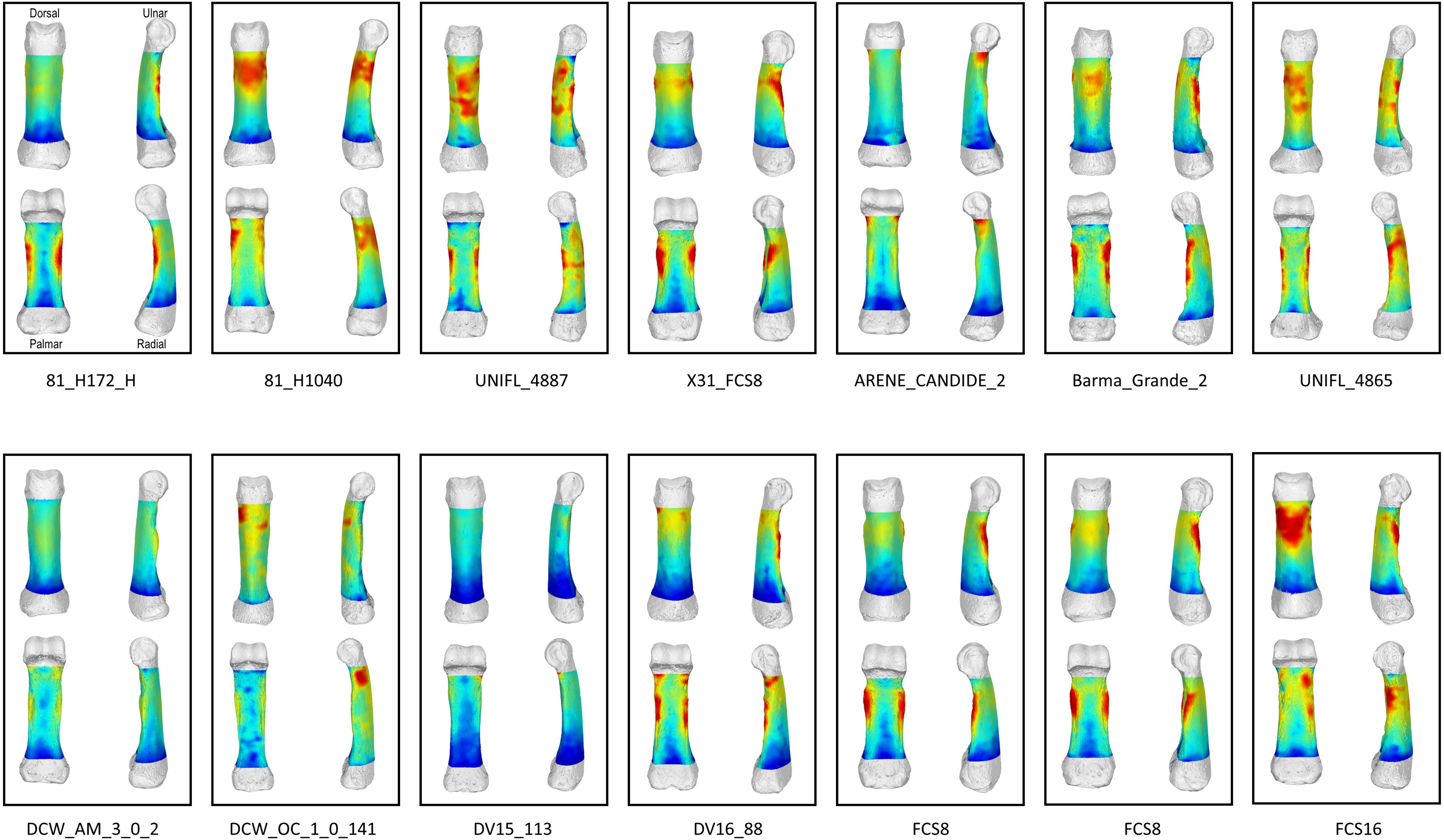

H. sapiens PP3

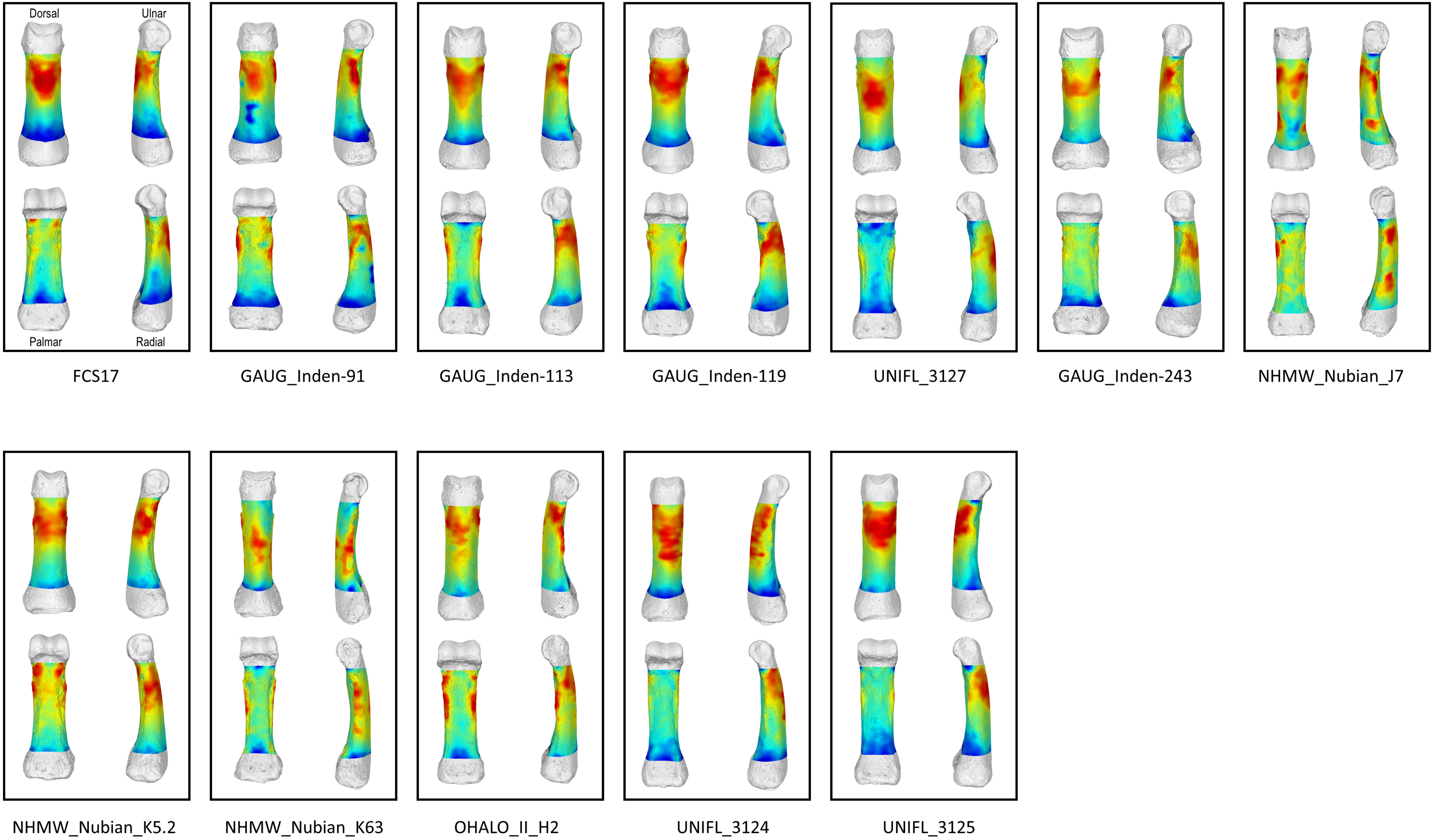

H. sapiens PP4

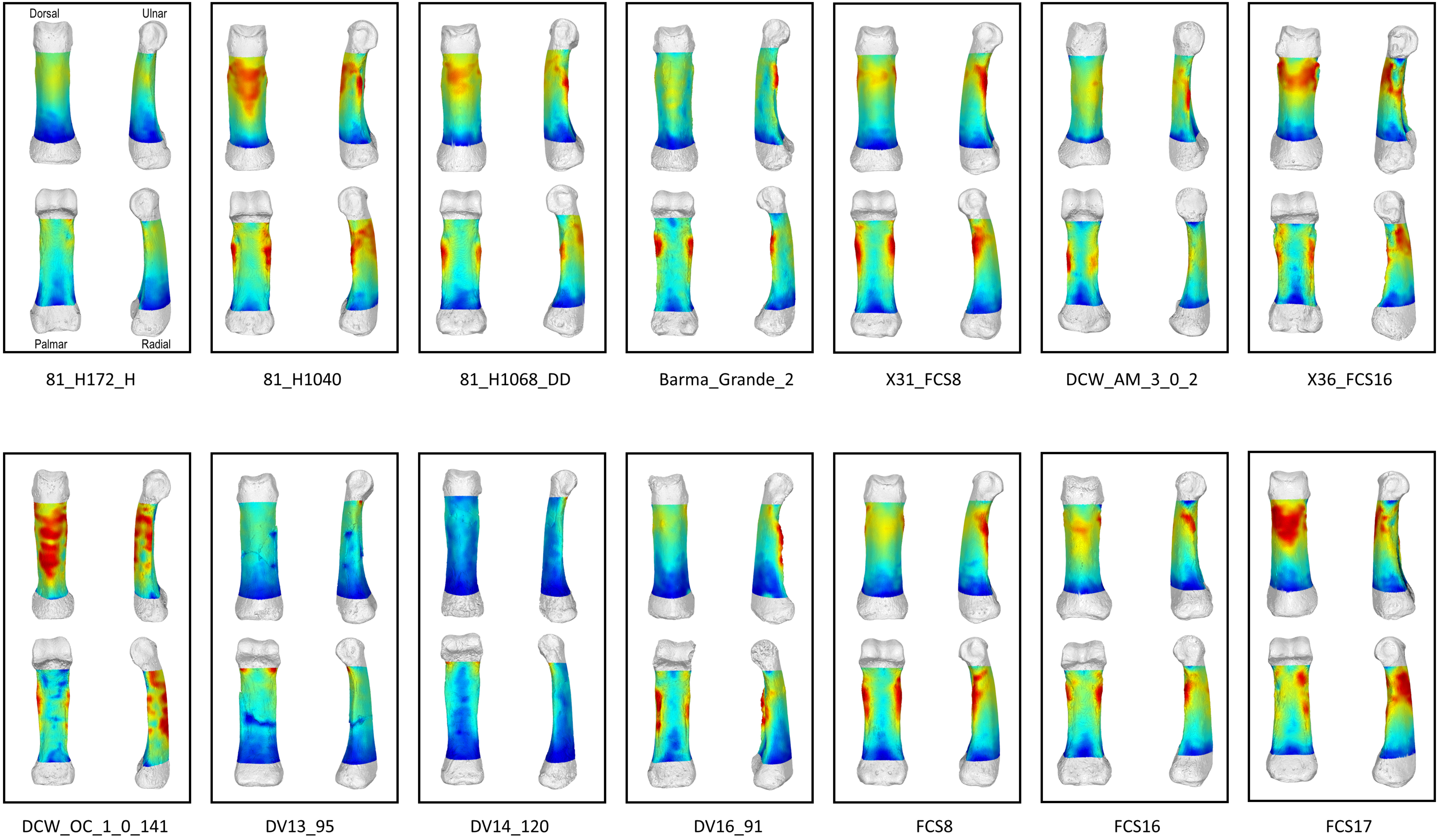

H. sapiens PP4

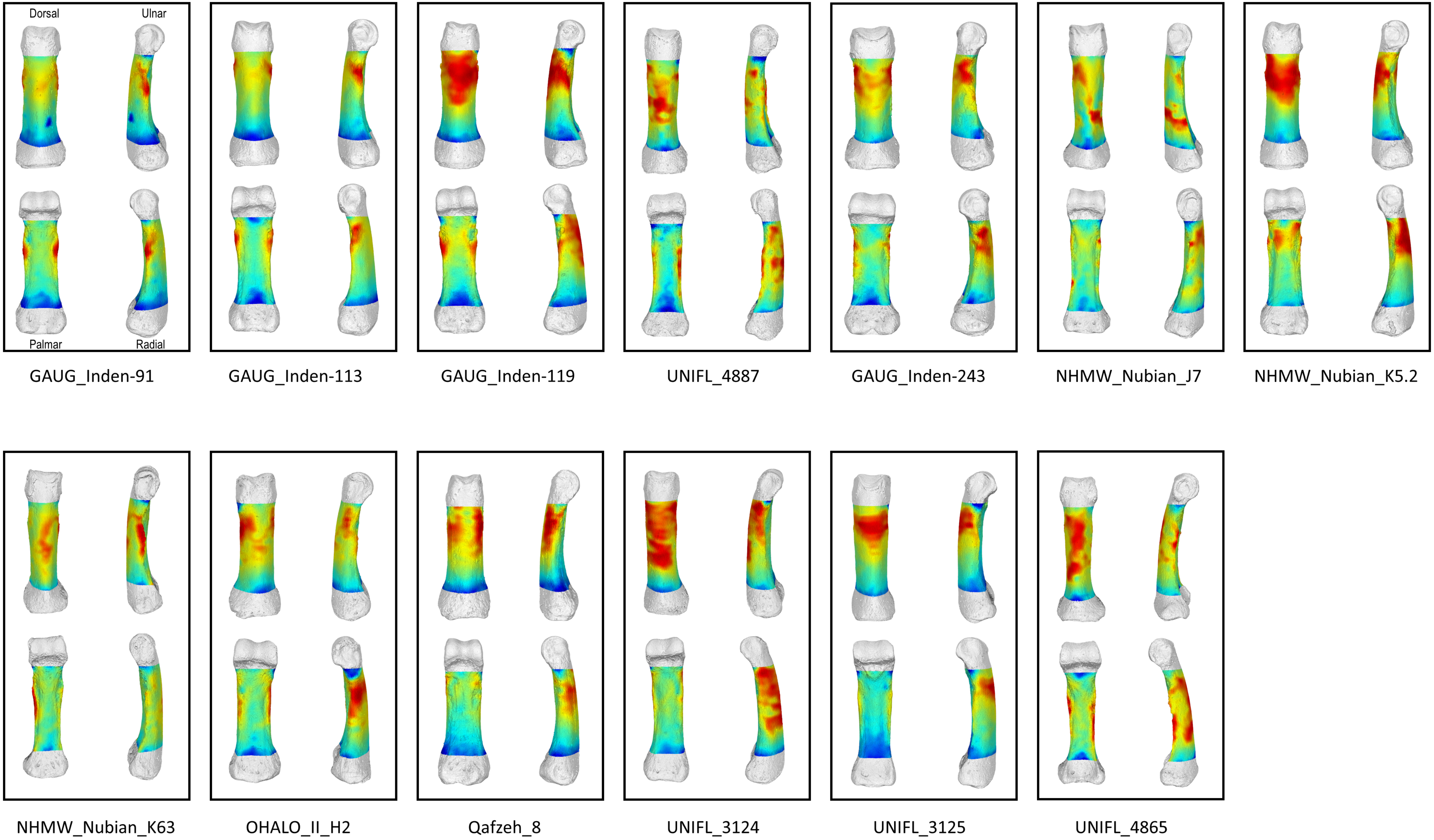

H. sapiens PP5

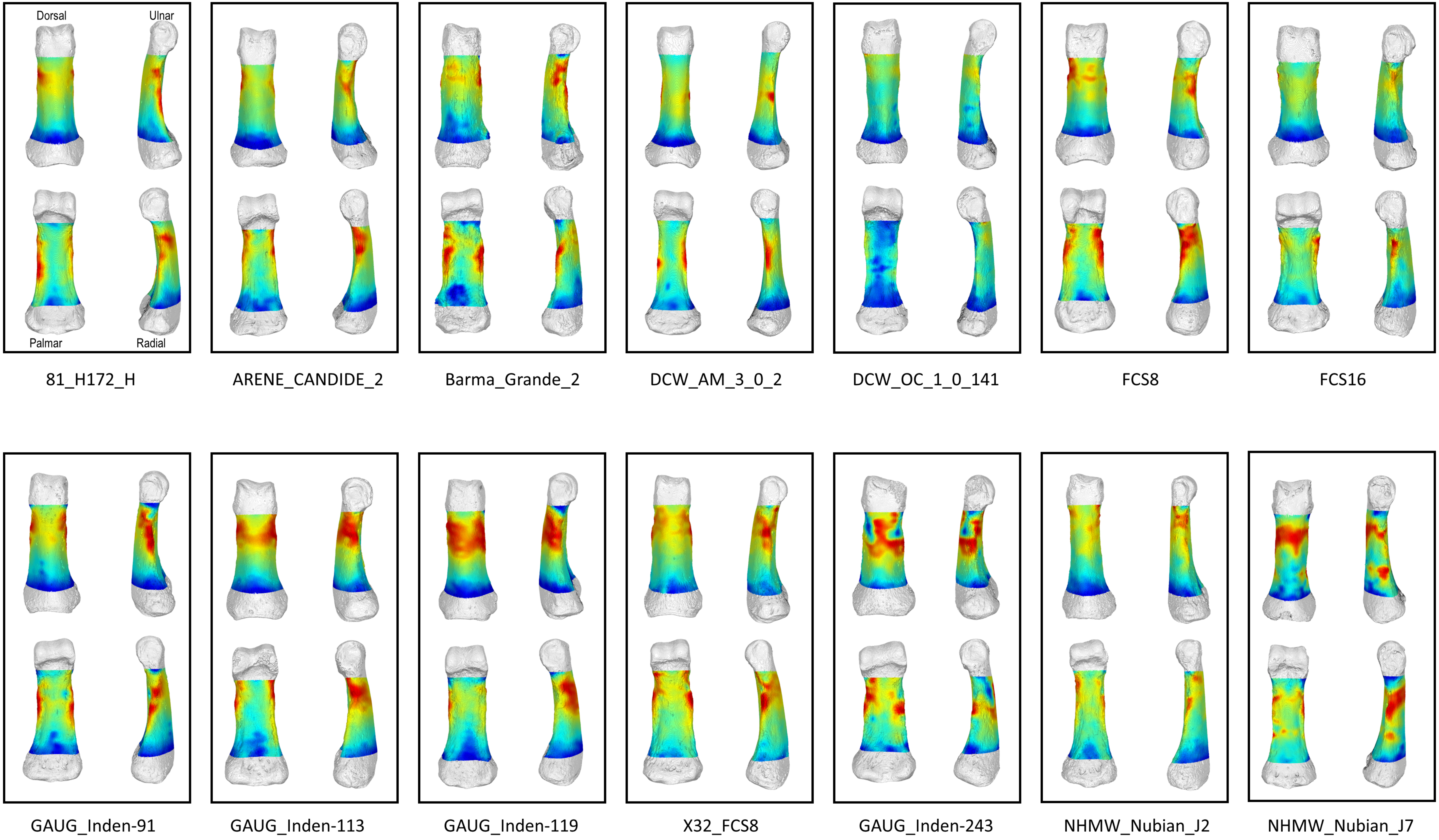

H. sapiens PP5

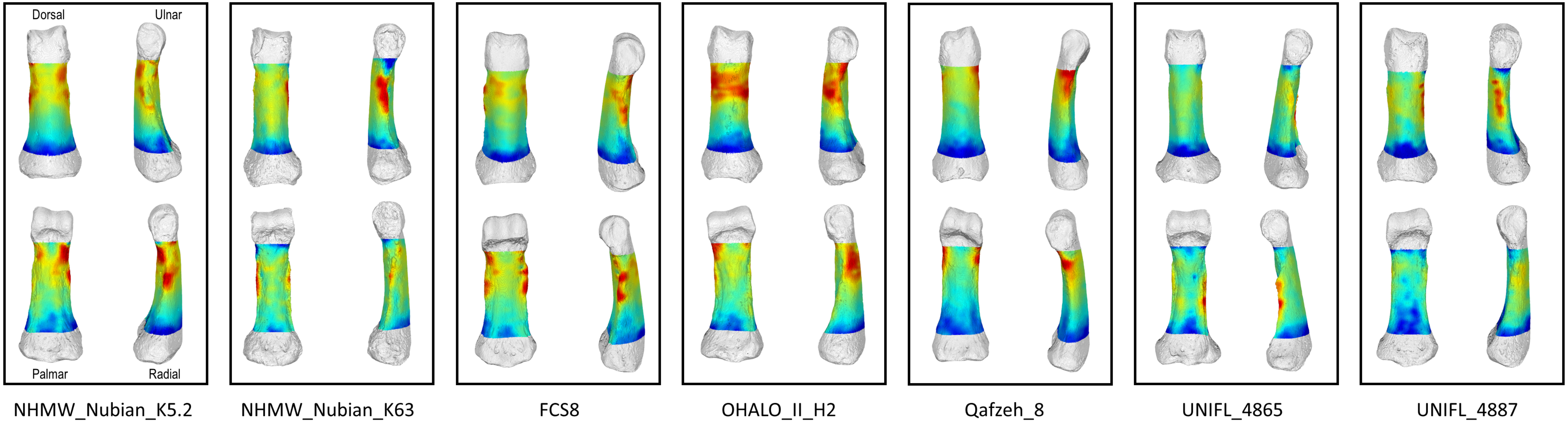

H. sapiens PP2 or PP4

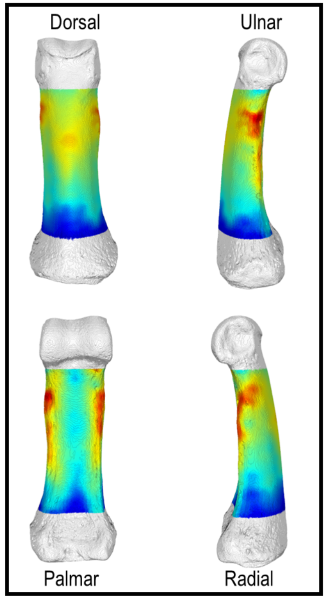

002_81_H172_H

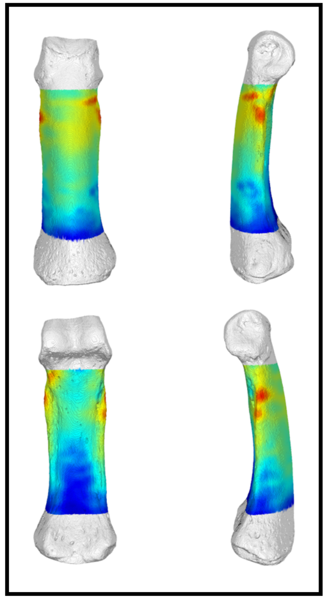

003_81_H172_H

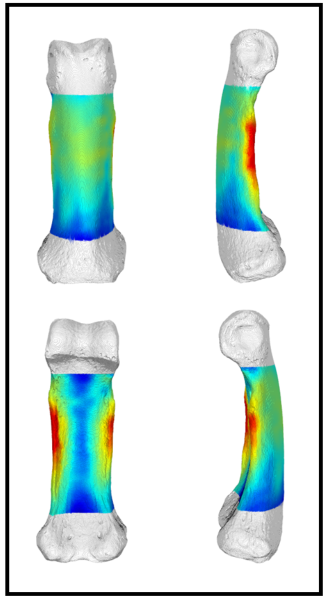

005_81_H172_H

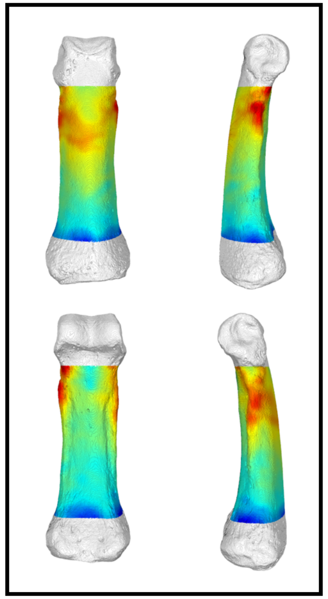

006_81_H172_H

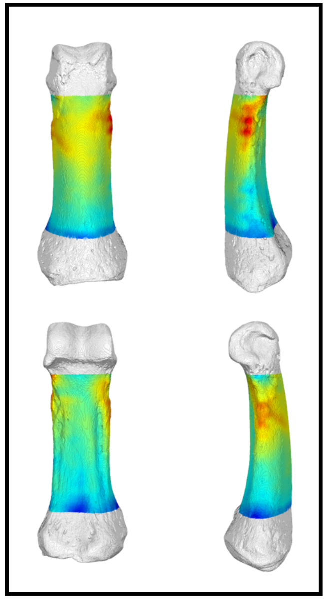

007_81_H172_H

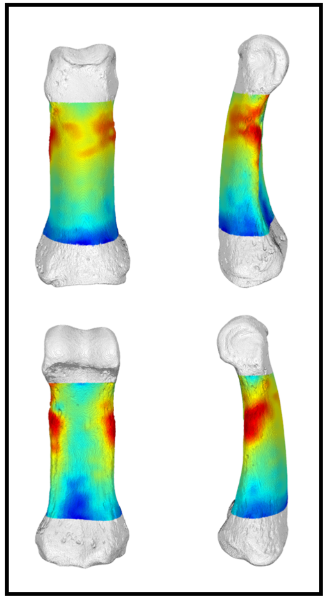

004_FCS8

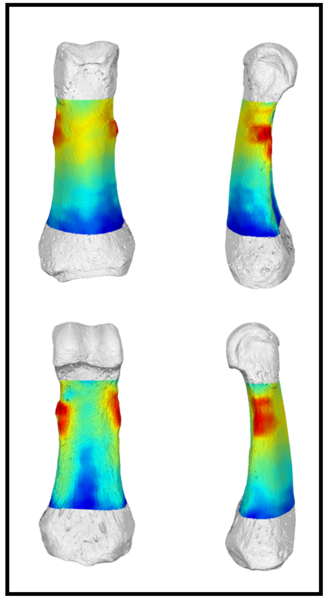

008_FCS8

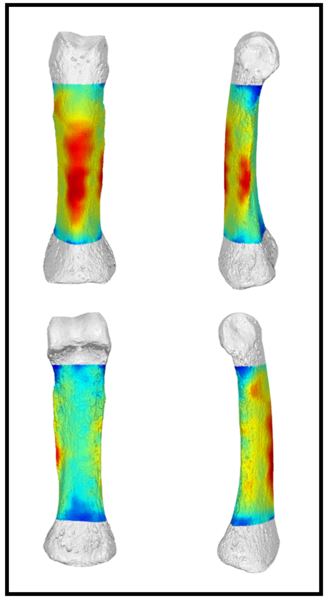

DCW_OC_1_0_26

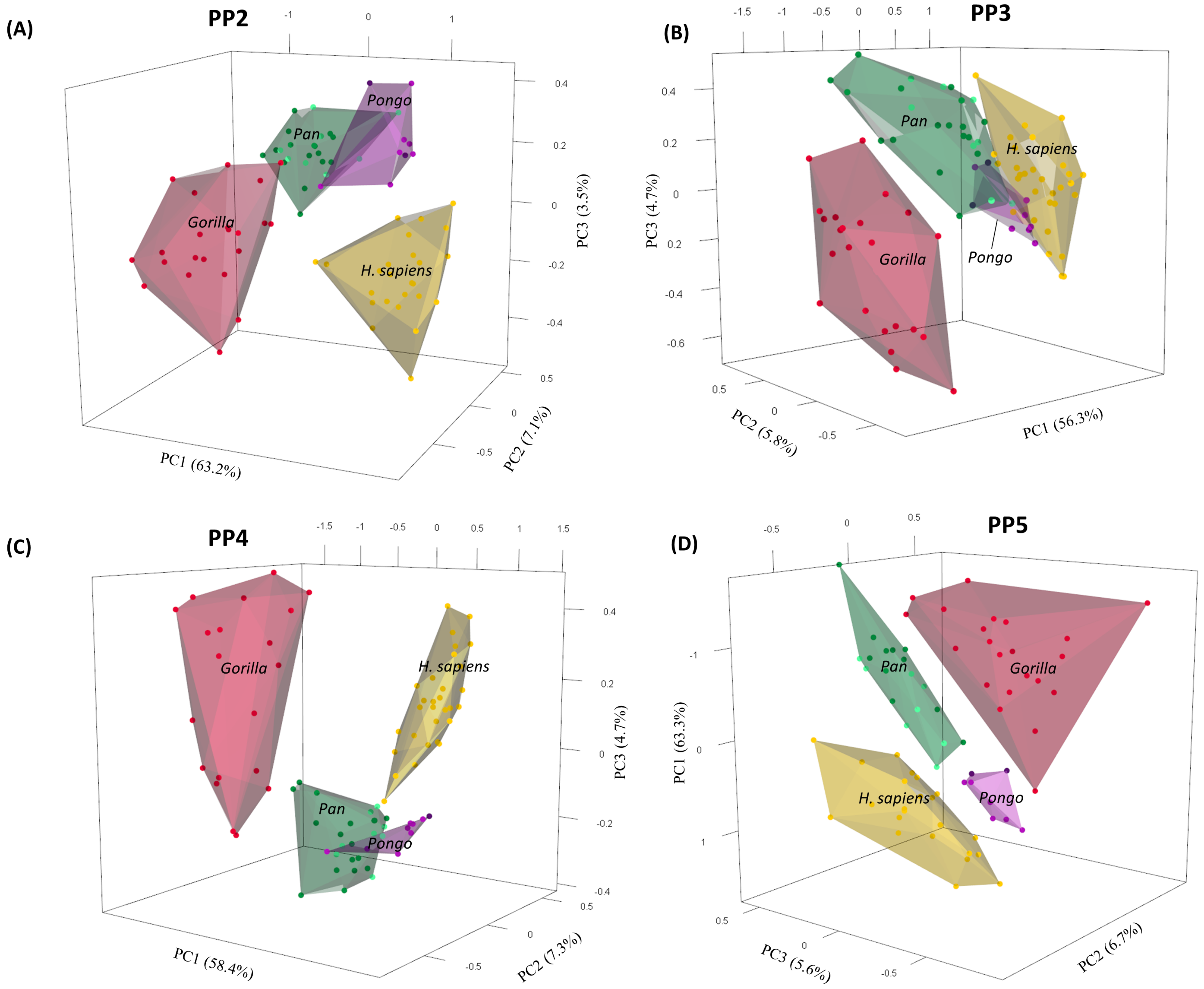

(A)
PP2
Pongo
Pan
Gorilla
H. sapiens
PC1 (63.2%)
PC2 (7.1%)
PC3 (3.5%)
(B)
PP3
Pan
H. sapiens
Gorilla
Pongo
PC1 (56.3%)
PC2 (5.8%)
PC3 (4.7%)
(C)
PP4
Gorilla
H. sapiens
Pan
Pongo
PC1 (58.4%)
PC2 (7.3%)
PC3 (4.7%)
(D)
PP5
Pan
Gorilla
H. sapiens
Pongo
PC1 (63.3%)
PC2 (6.7%)
PC3 (5.6%)

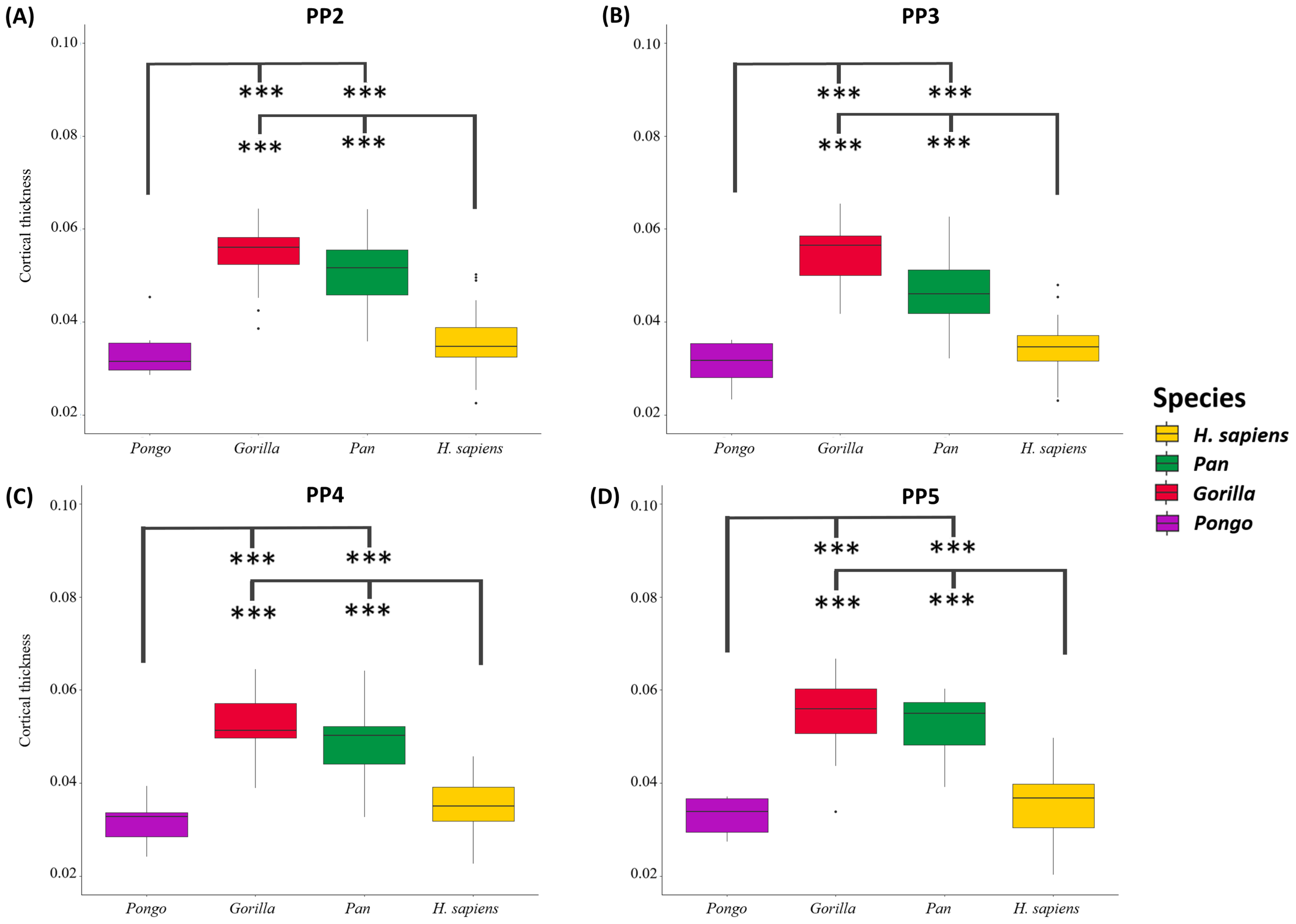

(A)
PP2
(B)
PP3
(C)
PP4
(D)
PP5
Cortical thickness
0.10
0.08
0.06
0.04
0.02
***
Pongo
Gorilla
Pan
H. sapiens
Species
H. sapiens
Pan
Gorilla
Pongo

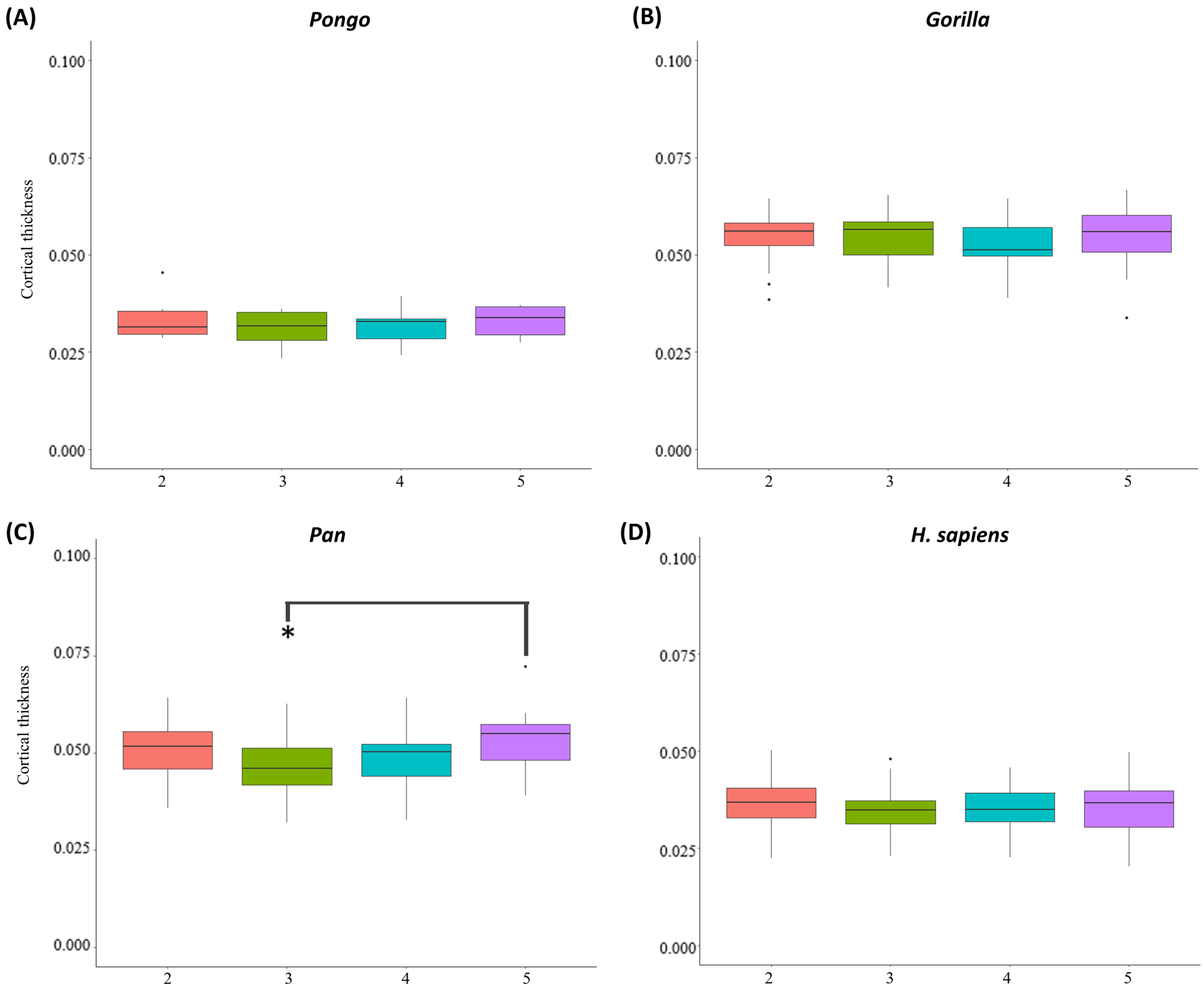
(A)
Pongo
(B)
Gorilla
(C)
Pan
(D)
H. sapiens
Cortical thickness
0.100
0.075
0.050
0.025
0.000
2
3
4
5
*

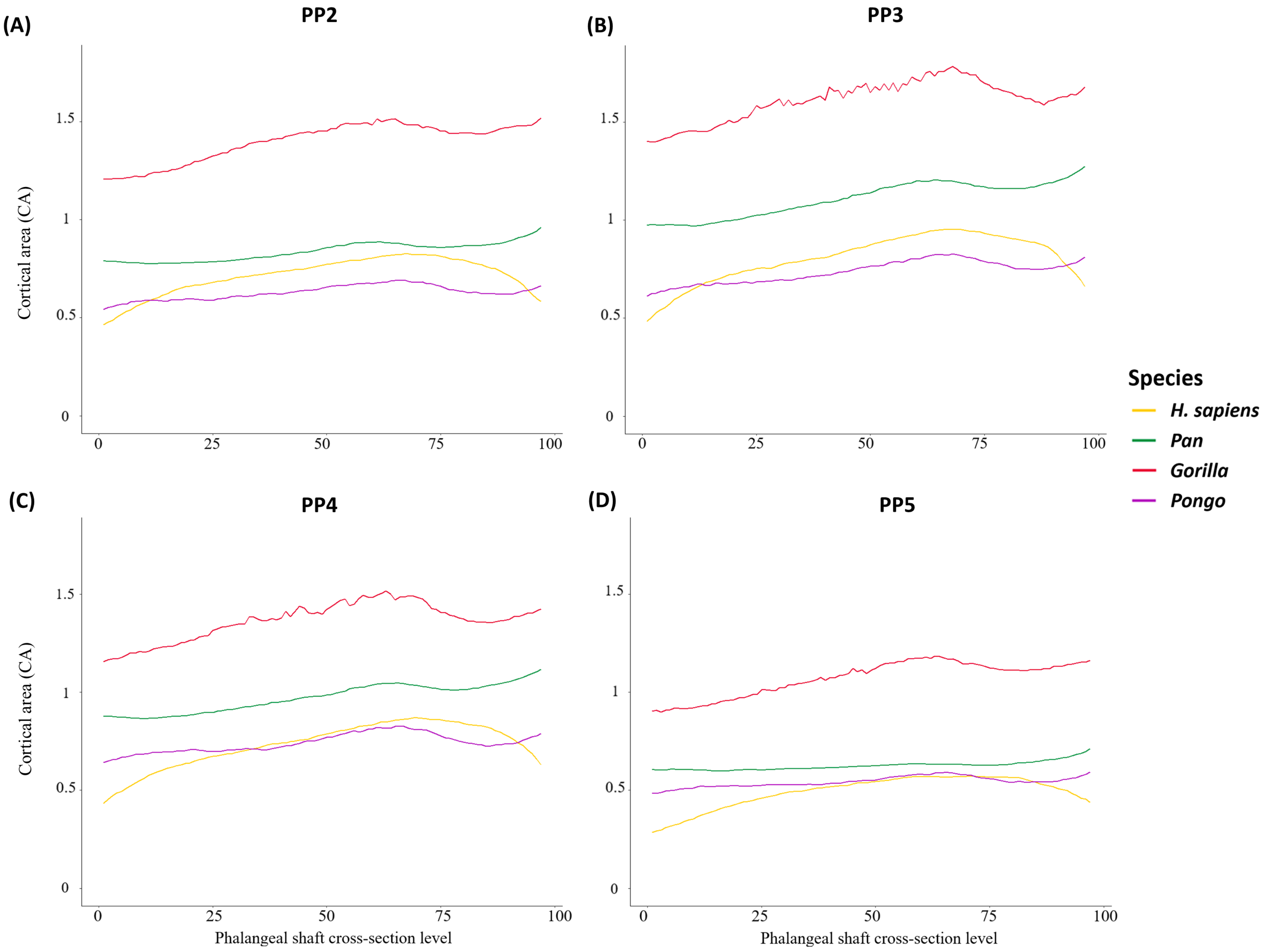
(A)
PP2
(B)
PP3
(C)
PP4
(D)
PP5
Cortical area (CA)
Phalangeal shaft cross-section level
0
0.5
1
1.5
0
25
50
75
100
Species
H. sapiens
Pan
Gorilla
Pongo

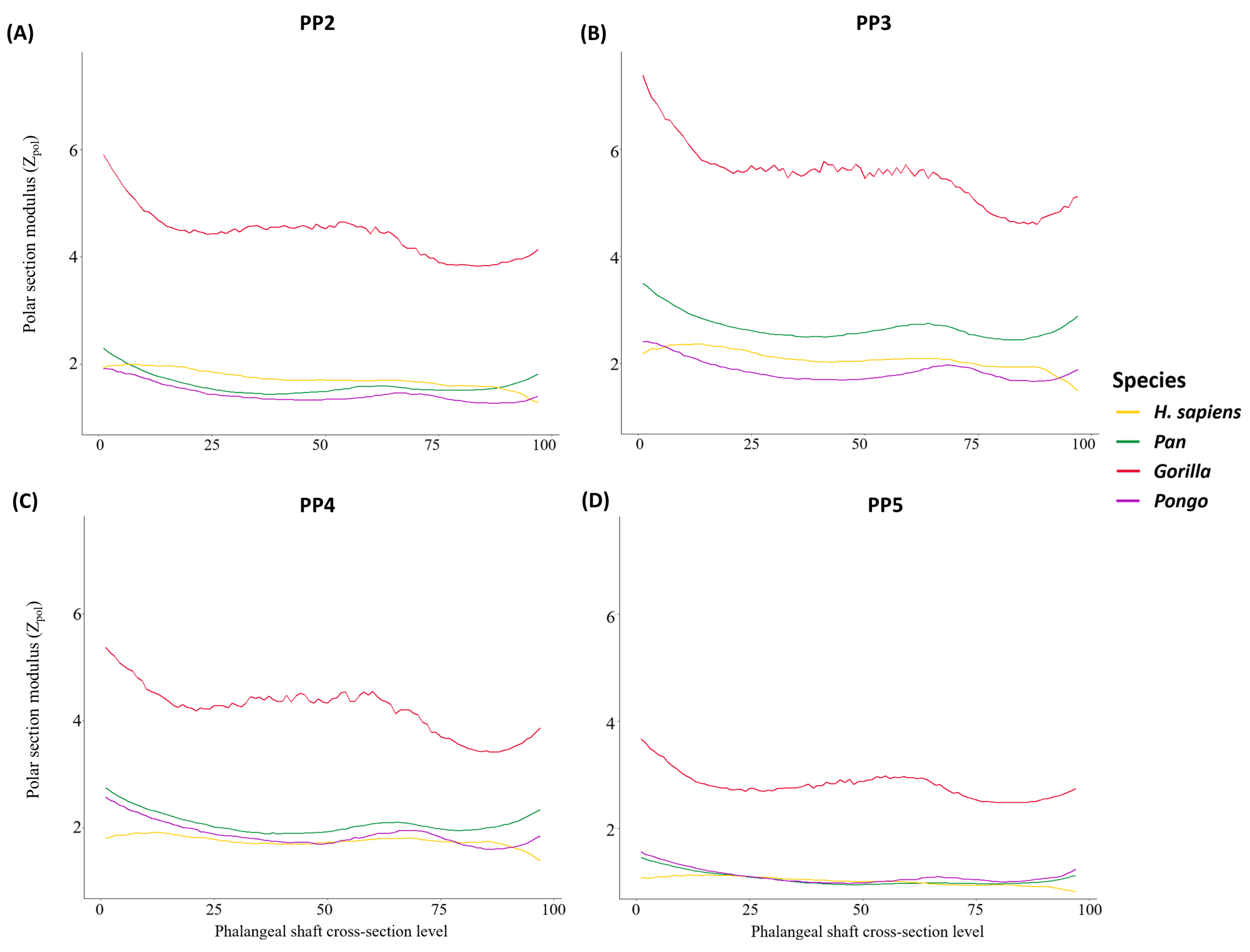

(A)
PP2
(B)
PP3
(C)
PP4
(D)
PP5
Polar section modulus (Z_{pol})
Phalangeal shaft cross-section level
0
25
50
75
100
2
4
6
Species
H. sapiens
Pan
Gorilla
Pongo

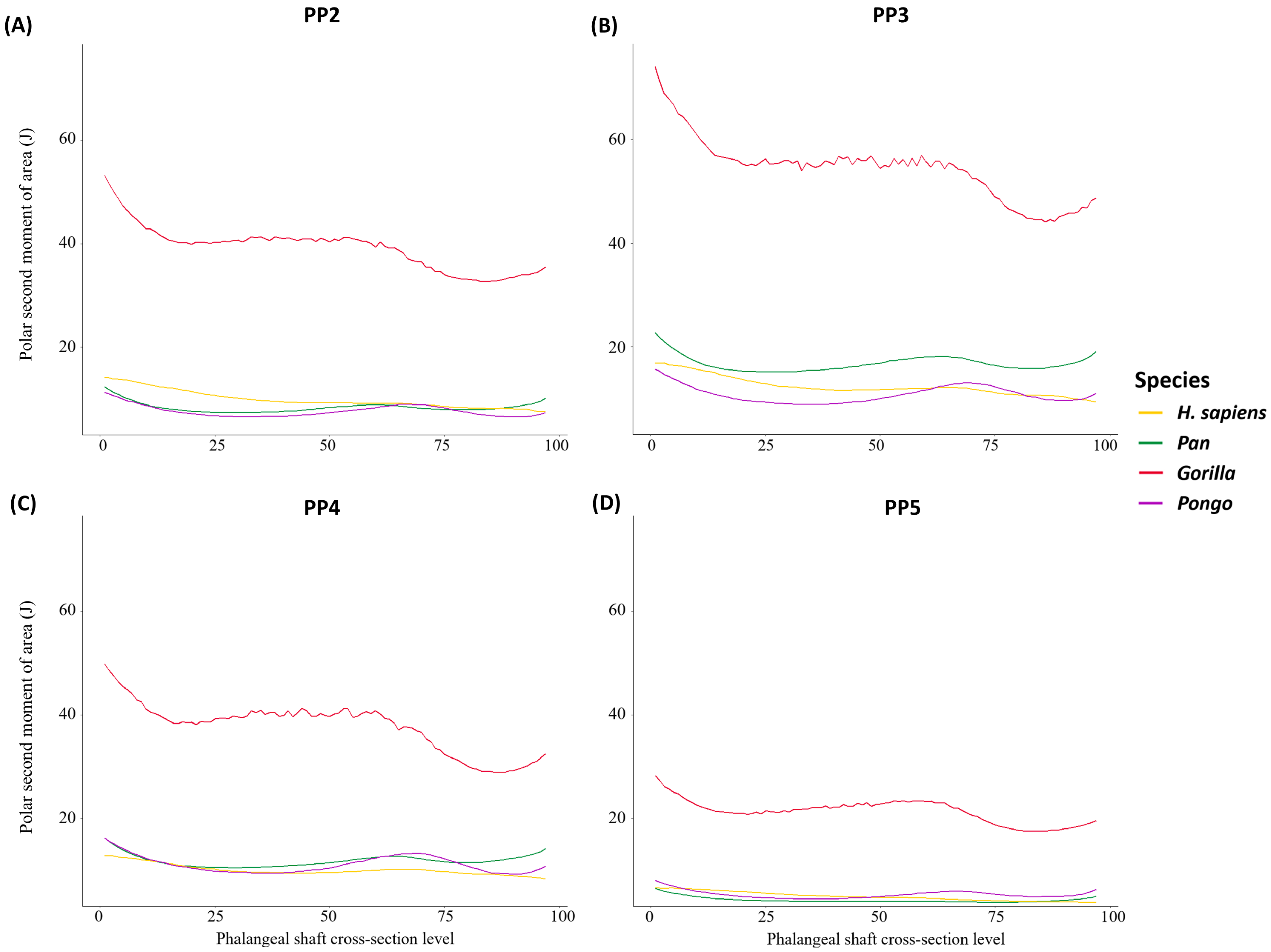
(A)
PP2
(B)
PP3
(C)
PP4
(D)
PP5
Polar second moment of area (J)
Phalangeal shaft cross-section level
0
25
50
75
100
20
40
60
Species
H. sapiens
Pan
Gorilla
Pongo

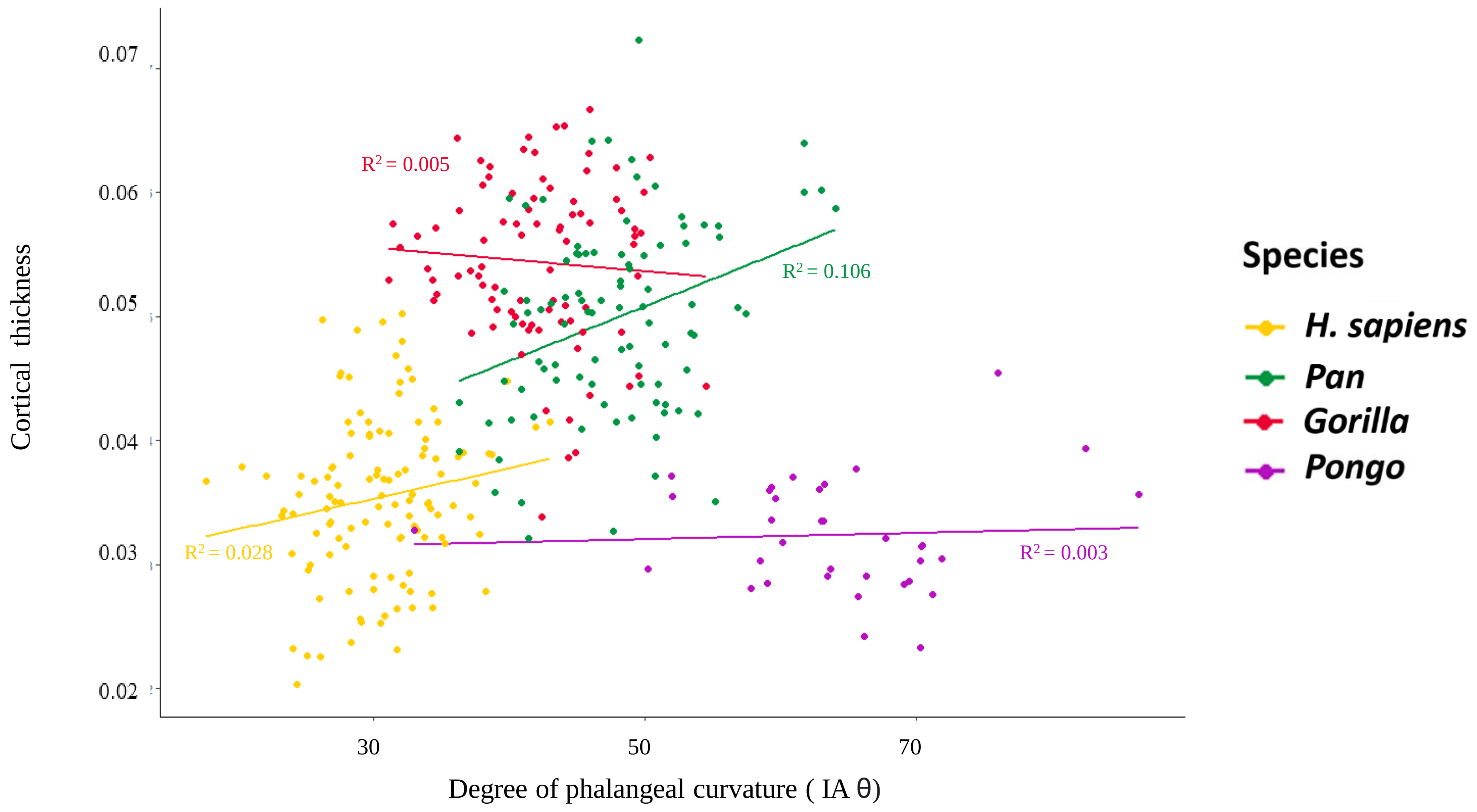

Cortical thickness
0.07
0.06
0.05
0.04
0.03
0.02
30
50
70
Degree of phalangeal curvature (IA θ)
R² = 0.005
R² = 0.106
R² = 0.028
R² = 0.003
Species
H. sapiens
Pan
Gorilla
Pongo
